# Expert Consensus on Morphofunctional Assessment in Disease-Related Malnutrition. Grade Review and Delphi Study

**DOI:** 10.3390/nu15030612

**Published:** 2023-01-25

**Authors:** José Manuel García-Almeida, Cristina García-García, María D. Ballesteros-Pomar, Gabriel Olveira, Juan J. Lopez-Gomez, Virginia Bellido, Irene Bretón Lesmes, Rosa Burgos, Alejandro Sanz-Paris, Pilar Matia-Martin, Francisco Botella Romero, Julia Ocon Breton, Ana Zugasti Murillo, Diego Bellido

**Affiliations:** 1 Department of Endocrinology and Nutrition, Hospital Clínico Universitario Virgen de la Victoria, 29010 Málaga, Spain; 2 IBIMA, Instituto de Investigación Biomédica y Plataforma en Nanomedicina, BIONAND, 29590 Málaga, Spain; 3 CIBEROBN, Centro de Investigación Biomédica en Red, Fisiopatología de la Obesidad y Nutrición, 28029 Madrid, Spain; 4 Department of Endocrinology and Nutrition, Hospital Quirónsalud, 29004 Málaga, Spain; 5 Facultad de Medicina, University of Málaga, 29010 Málaga, Spain; 6 PhD Program in Biomedicine, Translational Research and New Health Technologies, Faculty of Medicine, University of Málaga, 29071 Málaga, Spain; 7 Medical Director, Persan Farma, 35007 Las Palmas de Gran Canaria, Spain; 8 Department of Endocrinology and Nutrition, Complejo Asistencial Universitario de León, 24071 León, Spain; 9 Department of Endocrinology and Nutrition, Hospital Regional Universitario de Málaga, 29010 Málaga, Spain; 10 CIBERDEM, Centro de Investigación Biomédica en Red de Diabetes y Enfermedades Metabólicas Asociadas, Instituto de Salud Carlos III, 29010 Málaga, Spain; 11 Department of Medicine and Dermatology, University of Málaga, 29016 Málaga, Spain; 12 Department of Endocrinology and Nutrition, Hospital Clínico Universitario de Valladolid, 47003 Valladolid, Spain; 13 Department of Endocrinology and Nutrition, Hospital Universitario Virgen del Rocío, 41013 Sevilla, Spain; 14 Department of Endocrinology and Nutrition, Hospital General Universitario Gregorio Marañón, 28007 Madrid, Spain; 15 Nutritional Support Unit, Vall d’Hebron Barcelona Hospital Campus, 08035 Barcelona, Spain; 16 Department of Endocrinology and Nutrition, University Hospital Miguel Servet, 50009 Zaragoza, Spain; 17 Instituto de Investigación Sanitaria (IIS) Aragón, 50009 Zaragoza, Spain; 18 Department of Endocrinology and Nutrition, Hospital Clínico San Carlos, 28040 Madrid, Spain; 19 Instituto de Investigación Sanitaria del Hospital Clínico San Carlos (IdISSC), 28040 Madrid, Spain; 20 Medicine Department, Universidad Complutense, 28040 Madrid, Spain; 21 Department of Endocrinology and Nutrition, Complejo Hospitalario de Albacete, 02006 Albacete, Spain; 22 Department of Endocrinology and Nutrition, Hospital Clínico Universitario Lozano Blesa, 50009 Zaragoza, Spain; 23 Nutrition Department, Hospital Universitario de Navarra, 31008 Pamplona, Spain; 24 Department of Endocrinology and Nutrition, Complejo Hospitalario de Ferrol, 15405 Ferrol, Spain

**Keywords:** malnutrition, morphofunctional assessment, Delphi, GRADE, feasibility, clinical practice

## Abstract

Disease-related malnutrition (DRM) affects approximately a third of hospitalized patients and is associated with an increased risk of morbimortality. However, DRM is often underdiagnosed and undertreated. Our aim is to evaluate the prognostic value of morphofunctional tools and tests for nutritional assessment in clinical practice. A systematic literature review was conducted to identify studies relating to the morphofunctional assessment of nutritional status and mortality or complications. Evidence was evaluated using the GRADE (Grading of Recommendations, Assessment, Development, and Evaluations) method. Twelve GRADE recommendations were made and divided into seven topics: food intake and nutrient assimilation, anthropometry, biochemical analysis, hand grip strength, phase angle, muscle imaging, and functional status and quality of life. From these recommendations, 37 statements were developed and scored in a two-survey Delphi method by 183 experts. A consensus was reached on accepting 26/37 statements. Surveys had high internal consistency and high inter-rater reliability. In conclusion, evidence-based recommendations were made on the prognostic value of morphofunctional assessment tools and tests to assess malnutrition, most of which were found to be feasible in routine clinical practice, according to expert opinions.

## 1. Introduction

Malnutrition is a multifactorial disease that can be a result of starvation, disease, and/or advanced aging and is defined as “a state resulting from lack of intake or uptake of nutrition that leads to altered body composition (decreased fat free mass) and body cell mass leading to diminished physical and mental function and impaired clinical outcome from disease” [1]. Up to 31% of hospitalized patients are malnourished or at risk of malnutrition at admission [2,3,4,5], with the prevalence of malnutrition increasing with the length of stay [4]. Disease-related malnutrition (DRM), in particular, has been observed in 28–30% of hospitalized patients [6,7,8], and the prevalence is as high as 82% in hospitalized cancer patients [4,9].

Malnutrition increases the risk of complications, mortality, and infections in hospitalized patients; is associated with poor quality of life (QoL); and leads to longer hospital stays [3,6,7,9,10,11,12,13]. Although certain guidelines recommend approaches to assess malnutrition [1] and diets and protocols according to hospitalized patients’ needs [14], malnutrition is often underdiagnosed and undertreated [15,16], posing a serious health risk to patients. However, malnutrition is mostly treatable and; thus, it is of utmost importance to identify patients who are malnourished or at risk of malnutrition to provide them with effective support. 

There is no global consensus on the approach to malnutrition assessment; many parameters can be used, each with its own set of purposes and limitations [17,18,19,20]. Certain parameters, such as weight loss, body mass index (BMI), muscle mass, or food intake, are included in most malnutrition screening tools [18,19], while others, such as functional parameters and QoL, have gradually gained attention [17,18]. The criteria established with the Global Leadership Initiative on Malnutrition (GLIM) enabled a more comprehensive nutritional assessment by including the evaluation of muscle mass and disease burden/inflammation [19,21]. However, GLIM criteria do not provide an in-depth evaluation of body composition or functional status of patients, and there is a need for a set of parameters with prognostic values that go beyond nutritional assessment. This can be achieved with morphofunctional assessment, which provides a qualitative and quantitative evaluation of body composition and function using a series of tests that have prognostic and diagnostic values in DRM [22]. 

This study aims to develop evidence-based recommendations on the prognostic value of a series of morphofunctional tools and tests to assess malnutrition or the risk of malnutrition. In addition, the expert consensus was sought on the usefulness and feasibility of these tools and tests in routine clinical practice. 

## 2. Materials and Methods

### 2.1. Study Design

This study was endorsed by the Spanish Society of Endocrinology and Nutrition (SEEN). The study was coordinated by DBG, JMGA, and CGG and was developed and conducted by a scientific committee (MDBP, VBC, IBL, RBP, JJLG, PMM, GOF, ASP), a GRADE method coordination group (MDBP, GOF), a Delphi method coordination group (FBR, JOB, AZM), and the experts that participated in the Delphi method. The scientific committee comprised 8 endocrinologists who were experts in clinical nutrition. The clinical questions that guided the literature search and the recommendations were developed by the scientific committee over 8 meetings using the PICO (Patient, Intervention, Comparison, Outcome) framework. A systematic literature review was conducted, and the quality of the evidence was evaluated with the GRADE (Grading of Recommendations Assessment, Development, and Evaluation) method [23] to develop evidence-based recommendations. A series of statements were then developed by the scientific committee and evaluated with the Delphi method, an iterative process that enabled evaluations of issues by experts who provided feedback anonymously [24]. The Delphi method was conducted to find a consensus on the usefulness and feasibility of morphofunctional assessment tools in routine clinical practice. 

### 2.2. Literature Search

The literature search was conducted in agreement with the questions developed with the PICO framework for seven topics that cover several aspects of morphofunctional assessment: food intake and nutrient assimilation, anthropometry, biochemical analysis, hand grip strength (HGS), phase angle, muscle imaging, and functional status and QoL. PubMed and Embase databases were searched for studies published until May 2019 in English or Spanish; epidemiology and population studies were excluded. Of note, prior to initiating the Delphi method, a literature search was conducted, following the same strategy, to evaluate whether any relevant studies had been published since May 2019 so that they could be considered in the GRADE recommendations. The search terms used concerned malnutrition, mortality, complications, length of hospitalization, and QoL, together with other terms related to the seven topics: maldigestion, malabsorption, skinfold thickness, circumferences, albumin, pre-albumin, C-reactive protein, HGS, bioelectrical impedance, ultrasound, computed tomography, functional tests, and QoL (Supplementary Information S1).

### 2.3. GRADE Method 

The GRADE method is an approach that enables an explicit evaluation of evidence and provides a framework to develop recommendations [23]. GRADE was used to evaluate the evidence regarding the prognostic value of morphofunctional assessment tools in terms of mortality and complications. For each of the seven topics, an expert reviewed the literature, selected outcomes from the studies, rated their importance, and evaluated outcomes across studies; then, the evidence profile tables for each outcome were created, including a rating of the quality of the evidence, using GRADEpro GDT software (https://gradepro.org; accessed on 13 January 2020). The tables included outcomes, number of studies, study design, risk of bias, effect, quality of evidence, and importance. Another author from the scientific committee reviewed the evidence tables and conclusions drawn from the literature. The overall quality of evidence was graded across outcomes based on the lowest quality of critical outcomes. The scientific committee then made recommendations for each topic based on the literature findings and balancing consequences (e.g., benefits/harms, values and preferences, feasibility). 

### 2.4. Delphi Method

The scientific committee developed statements for the Delphi questionnaires; these regarded the usefulness and feasibility of morphofunctional assessment tests in routine clinical practice. A panel of 226 experts from the nutrition arm of SEEN was invited by email to participate, and their anonymity was maintained using a dedicated website for this study. Experts scored their agreement with each statement on a scale of 1 (strongly disagree) to 9 (strongly agree). After the first survey, the scientific committee received the results (median, 1st quartile–3rd quartile, degree of consensus) to evaluate which statements had to be included in the second survey and if any modification in the wording was required. In the second survey, experts were asked to score again the statements that did not reach consensus in the first survey. The Delphi method was conducted between November 2020 and April 2021. 

### 2.5. Statistical Analysis

Demographic values and Delphi responses were evaluated with descriptive statistics. The Delphi consensus was defined as at least two-thirds of the respondents selecting a score sub-category that encompassed the median score of the group: 1–3, reject statement; 4–6, undetermined; or 7–9, accept statement. The consistency of scoring was evaluated with Cronbach’s α (α > 0.7 was considered to indicate high reliability, and α > 0.9, very high reliability). Agreement between experts was evaluated with an intra-class correlation coefficient (r_i_). Correlation between the surveys was evaluated with the Spearman coefficient (r_s_) (negligible or poor: r_s_ = 0–0.25; weak: r_s_ = 0.26–0.50; moderate to strong: r_s_ = 0.51–0.75; and strong to very strong: r_s_ = 0.76–1) [25]. Qualitative agreement between surveys was evaluated with the Kappa index (*k*) by score sub-category taking into account the three response groups (1–3, 4–6, and 7–9) (slight agreement: *k* = 0–0.20; fair: *k* = 0.21–0.40; moderate: *k* = 0.41–0.60; substantial: *k* = 0.61–0.80); almost perfect: *k* = 0.81–1) [26]. The coefficient of variation (CV) was calculated for every survey, as well as the relative change in the second survey compared to the first (Second CV– First CV/First CV). A relative change in the CV of ≤10% was considered to indicate no large variability between surveys. Data were analyzed with SPSS 25.0 (IBM Corp. Released 2017. IBM SPSS Statistics for Windows, version 25.0. Armonk, NY, USA, IBM Corp.).

## 3. Results

### 3.1. Literature Review and GRADE Recommendations

The literature review yielded 1972 records; 284 articles covering the seven topics were selected for inclusion in this study (Supplementary Information S2). The evidence was evaluated following the GRADE method, which enabled the scientific committee to make 12 evidence-based recommendations based on the prognostic and clinical value of the tests and measures considered: one recommendation on food intake and nutrient assimilation, one on anthropometry, two on biochemical analysis, one on HGS, two on phase angle, three on muscle imaging, and two on functional tests and QoL (Table 1). 

The quality of the evidence to make these recommendations ranged from very low to moderate. There was insufficient evidence to make recommendations for the systematic use of food intake questionnaires alone or for the use of maldigestion or malabsorption tests in routine clinical practice. There was also insufficient evidence to make recommendations for the routine evaluation of serum prealbumin or C-reactive protein in patients with morbidity to evaluate the risk of morbidity and mortality. 

### 3.2. Delphi Method

#### 3.2.1. First Survey

Based on the evaluation of the evidence, the scientific committee developed 37 statements to be used in the Delphi method, divided among seven topics: five on food intake and nutrient assimilation, six on anthropometry, nine on biochemical analysis, two on HGS, two on phase angle, six on muscle imaging, and seven on functional status and QoL. Of the 226 experts invited to participate, 183 (80.9%) took part in the survey and scored the 37 statements. Respondents had a mean age of 42.8 years, were mostly female (65%), and had a median of 11 years of clinical experience. A consensus was reached on 24 of the 37 statements (64.8%), in all cases accepting them.

#### 3.2.2. Second Survey

Overall, 168 (91.8%) of the 183 experts that participated in the first survey submitted their responses in the second one (Table 2). 

This survey consisted of the 13 statements that had reached no or undetermined consensus in the first survey; statements S1, S5, S14, S34, S36, and S37 were presented with modifications. After this second round, two additional statements achieved consensus. Both surveys had a high internal consistency (first survey, Cronbach’s α = 0.862; second survey, Cronbach’s α = 0.840) and high inter-rater reliability (r_i_ = 0.860; second survey, r_i_ = 0.825). Spearman correlation values showed a moderate/strong to very strong quantitative agreement between surveys overall and by topic, except for statements relating to food intake and nutrient assimilation (Supplementary Table S1). The *k* index showed a moderate to good qualitative agreement between surveys overall and by topic, except for statements relating to food intake and nutrient assimilation, where the agreement was weak (Supplementary Table S1). The CVs of the first and second surveys were 0.293 ± 0.098 and 0.287 ± 0.083, respectively; the relative increase in CV was 2.05% and, given this low variability, a third survey was not conducted.

### 3.3. Alignment of Delphi Consensus with GRADE Recommendations

**Figure 1:**
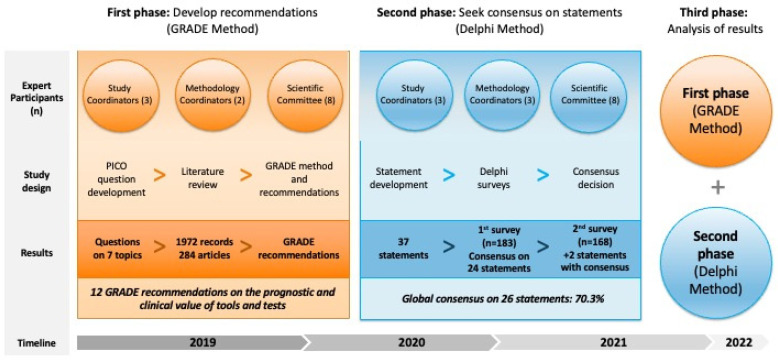
This study was developed and coordinated by several groups (study coordinators, methodology coordinators, and the scientific committee). PICO (Patient, Intervention, Comparison, Outcome) framework was used to develop clinical questions to guide the literature review, ultimately developing GRADE (Grading of Recommendations Assessment, Development and Evaluation) recommendations. A total of 37 statements were developed on 7 topics; these were evaluated in a Delphi method, achieving consensus on 26 statements.

Figure 1 summarizes the study design and main results.

**Figure 2:**
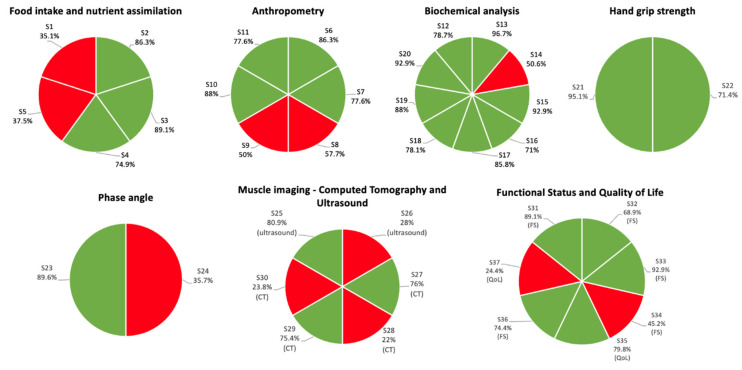
Consensus achieved on each statement is shown by topic. Green indicates consensus, red indicates no consensus. CT, computed tomography; FS, functional status; S, statement; QoL, quality of life.

The two Delphi surveys led to a consensus on 26 of the 37 statements (70.3%)—in all cases accepting them—regarding the usefulness and feasibility of morphofunctional tools and tests for assessing malnutrition (Table 3) (Figure 2). 

GRADE recommendations covered seven topics, and, with the Delphi method, a consensus was achieved on statements pertaining to each of these topics. The highest degree of consensus was achieved on the topics of HGS (2/2, 100%) (found useful and feasible), biochemical analysis (8/9, 88.8%) (found useful and feasible, except for the usefulness of one test), and functional status and QoL (5/7, 71.4%) (found useful, with lack of consensus concerning feasibility). A lower degree of consensus was achieved on anthropometry (4/6, 66.6%) (lack of consensus concerned only skinfold measurement, both its usefulness and feasibility), food intake and nutrient assimilation (3/5, 60%) (lack of consensus concerned usefulness in predicting prognosis in certain situations), phase angle (1/2, 50%) (found useful but not feasible), and muscle imaging (3/6, 50%) (found useful but not feasible). 

### 3.4. Subgroup Analysis of Statements with No Consensus

The 11 statements on which consensus was not reached were further reviewed to identify underlying explanations where possible. An analysis of scoring by respondent age (<37, 37–46, and >46 years) revealed a statistically significant difference for S14, with acceptance increasing with age but consensus on acceptance only being reached in the >46 age group (*p*-trend < 0.001). When evaluating responses by clinical experience (<7, 7–17, >17 years), increasing agreement with clinical experience was observed for S8 and S14 (*p* < 0.001). The type of hospital where respondents practiced (district, general or tertiary) had no significant impact on the level of acceptance. No statistically significant differences were found for the other statements.

## 4. Discussion

### 4.1. Insights from the Scientific Committee on the Delphi Results

In this study, the review of the literature on the association of morphofunctional tools and tests for malnutrition with mortality or complications across seven topics led to the development of 12 evidence-based recommendations (Table 1). 

These recommendations were the basis for a series of 37 statements that were used in a Delphi method to gather insights on the usefulness and feasibility of morphofunctional tools and tests in routine clinical practice by seeking consensus from a large group of experts. The topics of HGS, biochemical analysis, and functional status and QoL had the highest consensus. Overall, a consensus was achieved on 26 statements (Table 3), which ratified the findings from the published literature and also supported the recommendations made by the committee on: screening and nutritional assessment tools; circumferences; evaluation of serum albumin, prealbumin, and C-reactive protein; use of HGS; use of phase angle; imaging for muscle mass evaluation; functional status questionnaires and tests; and QoL questionnaires. The scientific committee reviewed the 11 statements on which consensus was not achieved and concluded that limited resources and/or time during patient consultation may explain why the Delphi respondents considered that skinfold measurement (S9), functional tests (S34), long quality-of-life questionnaires (S37), and computed tomography evaluation of muscle (S28, S30) were not feasible in routine clinical practice. These challenges are supported by the literature. For example, a systematic review found that, in most countries evaluated, patients spent less than 10 min in consultation with their primary care physician [27]. Moreover, computed tomography for muscle evaluation is an expensive approach that requires highly qualified personnel [28]. Muscle evaluation [29], quality of life questionnaires [30,31,32], and functional tests [33,34] are time-consuming, especially when considering the need for these tests and tools to be used together for an adequate overall morphofunctional analysis. The lack of consensus on accepting S8 may be due to the fact that it referred solely to skinfold measurements, whereas the GRADE recommendation made by the scientific committee referred to anthropometry in general, including both skinfolds and circumferences. Despite the lack of consensus on the feasibility of using the phase angle (S24) or using ultrasound for muscle evaluation (S26), the committee believed that they would rapidly be considered feasible in clinical practice, given the increasing evidence to support their value [35,36,37,38,39,40,41,42]. The lack of consensus on S1 and S5 suggested that further studies are needed to confirm the prognostic value of food intake assessment and malabsorption/maldigestion tests. The lack of consensus on S14 was surprising, considering the moderate quality of evidence supporting the prognostic value of albumin in patients with an acute disease [43,44]. Given that the Delphi experts did consider albumin evaluation in these patients to be feasible (S15), education for healthcare professionals is needed to highlight the prognostic value of albumin.

### 4.2. Implications for Clinical Practice 

A large study evaluating hospital units in 25 European countries found that only approximately half of them conducted nutritional screening and, overall, 27% of patients were classified as being “at nutritional risk” [2]. The integration of malnutrition screening in routine clinical practice has been found to be feasible and, in fact, increased considerably in a short time span [45]. Given the poor patient outcomes associated with malnutrition [5,46] and the fact that certain measures have prognostic values, integrating malnutrition assessment—not only screening—in routine care is of utmost importance. For example, a recent study found that HGS not only had prognostic values for mortality and risk of complications but also helped identify the hospitalized patients that would benefit most from nutritional support [47]. On this note, the results of the Delphi study we conducted based on GRADE recommendations indicated that experts in clinical nutrition and dietetics consider most of the approaches to be useful and, most importantly, feasible in clinical practice. 

A recent systematic review assessing malnutrition screening tools concluded that none of those evaluated tools had high validity, agreement, and reliability combined, according to the highest level of evidence [48]. Additionally, the validity and reliability of these tools ranged widely [48]. Most nutrition assessment tools also do not include parameters on prealbumin and albumin [49]. This supports the need for malnutrition evaluation that uses several tools and tests that are useful on a standalone basis—as concluded in the GRADE analysis and in the Delphi method—and that, in combination, provide a better picture of the patient’s nutritional status.

In this study, we identified a set of approaches that are feasible for morphofunctional assessment. These findings can guide the development of initiatives that (1) evaluate the degree to which these approaches are used in clinical practice; (2) evaluate the prognostic validity of these approaches integrated with GLIM criteria used for the diagnosis of malnutrition; (3) educate healthcare professionals on the use of these approaches; and (4) improve malnutrition assessment to, ultimately, improve patient health outcomes.

### 4.3. Strengths and Limitations

The main limitation of this study is that it only included the perspective of experts in Spain. Therefore, it would be interesting to replicate it with an international board of experts to view the cultural, geographical, and social differences represented at a larger level. However, all the experts were members of the Spanish Endocrinology and Nutrition Society (SEEN), the Spanish Society for Clinical Nutrition and Metabolism (SENPE), and the European Society for Clinical Nutrition and Metabolism (ESPEN) and were highly specialized in nutrition and dietetics; thus, the recommendations and consensus statements developed here may be applicable in other countries. 

One of the strengths of this study is the systematic methodology that was followed to formulate recommendations. The PICO framework was used to guide the literature search, and then the GRADE method was followed to assess the evidence. Moreover, the Delphi method enabled a systematic approach to finding consensus while maintaining the anonymity of responders and achieved a high level of participation from experts in clinical nutrition. Another strength of this study is the evaluation of the usefulness and feasibility of nutritional assessment tools as separate concepts. Most healthcare professionals involved in the routine care of patients with malnutrition or at risk of malnutrition are not generally as specialized in this topic as the experts who participated in this study. However, the group of Delphi respondents was heterogeneous in the type of healthcare professional represented, the hospital setting where they practiced, and their geographical location (representing 16 of the 17 autonomous regions of Spain), which increased the external validity of our findings. Additionally, the experts evaluated the feasibility of each tool and test considered here, which ensures that the recommendations are grounded in real-world clinical practice and do not merely reflect the literature.

## 5. Conclusions

In this study, we reviewed the literature to make recommendations on morphofunctional assessment approaches based on their prognostic value in patients who are malnourished or at risk of malnutrition. A large group of experts participating in a Delphi method deemed many of the tools and tests considered here to be useful and feasible in routine clinical practice. Thus, the implementation of these tools and tests is recommendable to improve diagnosis, therapeutic treatments, and patient outcomes.

## Figures and Tables

**Table 1:** Evidence-based recommendations following GRADE method for patients who are hospitalized or receiving outpatient care.

No.	Topic	Strength of Recommendation	Quality of Evidence	Recommendation
R1	Food intake and nutrient assimilation	Strong	Moderate	Oral food intake questionnaires, especially those included in MNA and SGA, should be used in routine nutritional assessment of malnourished patients or patients at risk of malnutrition.
R2	Anthropometry (skinfolds and circumference)	Strong	Moderate	Anthropometry—including skinfold and circumference measurements—should be conducted during nutritional assessment to predict the prognosis of patients who are malnourished or who have diseases that increase the risk of disease-related malnutrition.
R3	Biochemical analysis	Strong	Moderate	Serum albumin should be evaluated prior to a major surgery to predict complications and mortality.
R4	Biochemical analysis	Strong	Moderate	Serum albumin should be evaluated in patients with acute diseases and in the elderly to predict complications and mortality.
R5	Hand grip strength	Strong	Low–Moderate	Routine nutritional assessment of patients with acute or chronic diseases should include the hand-grip strength, given its prognostic value and ease of use (it is affordable and can be standardized).
R6	Phase angle	Strong	Low–Moderate	The phase angle, measured by bioelectrical impedance analysis, can be used for predicting mortality in patients with disease-related malnutrition.
R7	Phase angle	Strong	Low–Moderate	The phase angle, measured by bioelectrical impedance analysis, can be used for predicting complications in patients with disease-related malnutrition.
R8	Muscle imaging	Moderate	Very Low–Low	Evaluation of the quantity and quality of muscle mass with ultrasound is suggested for predicting clinical prognosis when other body composition measurement methods are not available.
R9	Muscle imaging	Weak	Low	Evaluation of quantity and quality of muscle mass (low attenuation/myosteatosis) with computed tomography is suggested for predicting clinical prognosis when this technique is routinely used.
R10	Muscle imaging	Strong	Moderate	Evaluation of change in muscle mass with computed tomography is recommended for predicting clinical prognosis when this technique is routinely used.
R11	Functional status and quality of life	Strong	Low–Moderate	Functional tests should be added to the routine nutritional assessment to predict mortality and complications in malnourished patients with acute or chronic diseases.
R12	Functional status and quality of life	Moderate	Very Low–Low	Quality of life test may be added to the routine nutritional assessment for predicting mortality and complications in malnourished patients with acute or chronic diseases.

MNA, mini nutritional assessment; SGA, subjective global assessment.

**Table 2:** Demographics of experts participating in the Delphi survey (N = 168).

Characteristic	N (%)
Age (mean ± SD), years	42.7 ± 10.2
Female	108 (64.3)
Professional experience (median [IQR]), years	11 (5–22)
Type of healthcare professional	
Medical doctor	165 (98.2)
Nurse	1 (0.6)
Nutritionist	2 (1.2)
Specialty	
Endocrinology and nutrition	164 (97.6)
Internal medicine	1 (0.6)
Not specified	3 (1.8)
Position	
Head of department	27 (16.1)
Consultant	130 (77.4)
Resident	5 (3)
Others	6 (3.6) *
Type of hospital (financing)	
Public	141 (83.9)
Private	2 (1.2)
Mixed	25 (14.9)
Type of hospital (level of healthcare)	
District	18 (10.7)
General	54 (32.1)
Tertiary	96 (57.1)
Works in a nutrition unit	149 (88.7)
Healthcare professional profiles working in the nutrition units	
Medical doctor	149 (100)
Nurse	106 (71.1)
Nutritionist	94 (63.1)

IQR, interquartile range. * Others: 1 Clinical nutrition and dietetics coordinator, 1 adjunct doctor, 1 adjunct university professor, 3 not specified.

**Table 3:** Results from Delphi method.

No.	Statement	Median (Q1–Q3)	Min–Max	Median Range	Accept Statement; n (%)	Reject Statement; n (%)	Consensus/Decision
S1	The assessment of food intake alone during anamnesis in patients with malnutrition or at risk of malnutrition has uncertain usefulness in predicting prognosis	5 (3–7)	1–9	4–6	59 (35.1%)	66 (39.3%)	No consensus
S2	Screening tools that include food intake assessment (MUST, NRS2002, SNAQ and MNA-SF) are the tools of choice for initial assessment of patients with malnutrition or at risk of malnutrition, given their clinical prognostic value	8 (7–9)	1–9	7–9	158 (86.3%)	7 (3.8%)	Consensus/Accept statement
S3	Nutritional assessment tools that include food intake evaluation (MNA and SGA) are the tools of choice for evaluating patients with malnutrition or at risk of malnutrition, given their clinical prognostic value	8 (7–9)	2–9	7–9	163 (89.1%)	6 (3.3%)	Consensus/Accept statement
S4	Malabsorption and maldigestion tests are useful for the diagnosis of diseases that deteriorate the nutritional status of patients and for adapting the nutritional treatment	7 (6–8)	1–9	7–9	137 (74.9%)	13 (7.1%)	Consensus/Accept statement
S5	Malabsorption and maldigestion tests in patients with malnutrition or at risk of malnutrition have uncertain usefulness in predicting prognosis	5 (3–7)	1–9	4–6	63 (37.5%)	55 (32.7%)	No consensus
S6	Height and weight measurements as part of the nutritional assessment of patients with malnutrition or at risk of malnutrition are useful for predicting prognosis	8 (7–9)	1–9	7–9	158 (86.3%)	3 (1.6%)	Consensus/Accept statement
S7	Height and weight measurements are feasible in routine clinical practice	8 (7–9)	1–9	7–9	142 (77.6%)	6 (3.3%)	Consensus/Accept statement
S8	Skinfold measurement as part of the nutritional assessment of patients with malnutrition or at risk of malnutrition is useful for predicting prognosis	7 (5–8)	1–9	7–9	97 (57.7%)	23 (13.7%)	No consensus
S9	Skinfold measurement is feasible in routine clinical practice	6.5 (4–8)	1–9	7–9	84 (50%)	32 (19%)	No consensus
S10	Arm and calf circumference measurements as part of the nutritional assessment of patients with malnutrition or at risk of malnutrition are useful for predicting prognosis	8 (7–9)	2–9	7–9	161 (88.0%)	2 (1.1%)	Consensus/Accept statement
S11	Arm and calf circumference measurements are feasible in routine clinical practice	8 (7–8)	1–9	7–9	142 (77.6%)	11 (6.0%)	Consensus/Accept statement
S12	Evaluation of preoperative serum albumin as part of the nutritional assessment of patients with malnutrition or at risk of malnutrition is useful for predicting prognosis	8 (7–9)	1–9	7–9	144 (78.7%)	13 (7.1%)	Consensus/Accept statement
S13	Evaluation of preoperative serum albumin is feasible in routine clinical practice	9 (8–9)	3–9	7–9	177 (96.7%)	1 (0.5%)	Consensus/Accept statement
S14	Evaluation of serum albumin when patients with malnutrition or at risk of malnutrition and an acute disease are hospitalized is useful for predicting prognosis	7 (4–8)	1–9	7–9	85 (50.6%)	41 (24.4%)	No consensus
S15	Evaluation of serum albumin when patients with malnutrition or at risk of malnutrition and an acute disease are hospitalized is feasible in routine clinical practice	9 (8–9)	1–9	7–9	170 (92.9%)	3 (1.6%)	Consensus/Accept statement
S16	Evaluation of serum albumin as part of the nutritional assessment of elderly patients with malnutrition or at risk of malnutrition is useful for predicting prognosis	7 (6–8)	1–9	7–9	130 (71.0%)	11 (6.0%)	Consensus/Accept statement
S17	Evaluation of serum albumin in elderly patients with malnutrition or at risk of malnutrition is feasible in routine clinical practice	8 (7–9)	3–9	7–9	157 (85.8%)	2 (1.1%)	Consensus/Accept statement
S18	Evaluation of prealbumin is feasible in routine clinical practice	8 (7–9)	2–9	7–9	143 (78.1%)	9 (4.9%)	Consensus/Accept statement
S19	Evaluation of C-reactive protein together with albumin as part of the nutritional assessment of patients with malnutrition or at risk of malnutrition is useful for predicting prognosis	9 (8–9)	1–9	7–9	161 (88.0%)	4 (2.2%)	Consensus/Accept statement
S20	Evaluation of C-reactive protein is feasible in routine clinical practice	9 (8–9)	2–9	7–9	170 (92.9%)	1 (0.5%)	Consensus/Accept statement
S21	Use of hand grip strength as part of the nutritional assessment of patients with malnutrition or at risk of malnutrition is useful for predicting prognosis	9 (8–9)	5–9	7–9	174 (95.1%)	0 (0%)	Consensus/Accept statement
S22	Use of hand grip strength is feasible in routine clinical practice	8 (6–9)	2–9	7–9	120 (71.4%)	12 (7.1%)	Consensus/Accept statement
S23	The phase angle measured by bioelectrical impedance assessment in patients with malnutrition or at risk of malnutrition is useful for predicting prognosis	8 (7–9)	3–9	7–9	164 (89.6%)	2 (1.1%)	Consensus/Accept statement
S24	Measurement of the phase angle by bioelectrical impedance assessment is feasible in routine clinical practice	6 (3–7)	1–9	4–6	60 (35.7%)	42 (25%)	No consensus
S25	Ultrasound evaluation of the quantity and quality of muscle as part of the nutritional assessment of patients with malnutrition or at risk of malnutrition is useful for predicting prognosis	8 (7–9)	1–9	7–9	148 (80.9%)	3 (1.6%)	Consensus/Accept statement
S26	Ultrasound evaluation of the quantity and quality of muscle is feasible in routine clinical practice	5 (3–7)	1–9	4–6	47 (28%)	53 (31.5%)	No consensus
S27	Computed tomography evaluation of the quantity and quality of muscle as part of the nutritional assessment of patients with malnutrition or at risk of malnutrition is useful for predicting prognosis	8 (7–9)	1–9	7–9	139 (76.0%)	14 (7.7%)	Consensus/Accept statement
S28	Computed tomography evaluation of the quantity and quality of muscle, when clinically indicated for follow-up, is feasible in routine clinical practice	5 (3–6)	1–9	4–6	37 (22%)	59 (35.1%)	No consensus
S29	Computed tomography evaluation of changes in muscle mass (when this technique is available for diagnosis/follow-up of the disease) as part of the nutritional assessment of patients is useful for predicting prognosis	8 (7–8)	1–9	7–9	138 (75.4%)	11 (6.0%)	Consensus/Accept statement
S30	When computed tomography is required for follow-up of patients, measuring changes in muscle mass is feasible in routine clinical practice	5 (3–6)	1–9	4–6	40 (23.8%)	59 (35.1%)	No consensus
S31	Functional status questionnaires (Barthel index, Katz index) as part of the nutritional assessment of patients with malnutrition or at risk of malnutrition are useful for predicting prognosis	8 (7–9)	2–9	7–9	163 (89.1%)	2 (1.1%)	Consensus/Accept statement
S32	The use of functional status questionnaires (Barthel index, Katz index) is feasible in routine clinical practice	7 (6–8)	2–9	7–9	126 (68.9%)	6 (3.3%)	Consensus/Accept statement
S33	The use of one or several functional tests (6-min walk test, 10-m walk test, short physical performance battery, timed up and go test, one-leg standing time) as part of the nutritional assessment of patients with malnutrition or at risk of malnutrition is useful for predicting prognosis	8 (7–9)	4–9	7–9	170 (92.9%)	0 (0%)	Consensus/Accept statement
S34	The use of one or several functional tests (6-min walk test, 10-m walk test, short physical performance battery, timed up and go test, one-leg standing time) is feasible in routine clinical practice	6 (4–7)	1–9	4–6	76 (45.2%)	30 (17.9%)	No consensus
S35	Quality of life questionnaires (ECOG performance status, Karnofsky scale, SF-36, KDQOL-SF, EQ-5D-5L) as part of the nutritional assessment of patients with malnutrition or at risk of malnutrition are useful for predicting prognosis	8 (7–9)	2–9	7–9	146 (79.8%)	3 (1.6%)	Consensus/Accept statement
S36	The use of short functional tests (ECOG performance status or Karnofsky scale) is feasible in routine clinical practice	8(6–8)	2–9	7–9	125 (74.4%)	3 (1.8%)	Consensus/Accept statement
S37	The use of long questionnaires of quality of life (SF-36, KDQOL-SF, EQ-5D-5L) is feasible in routine clinical practice	5.5 (3–6)	1–9	4–6	41 (24.4%)	42 (25%)	No consensus

ECOG, Eastern Cooperative Oncology Group; EQ-5D-5L, EuroQol 5-dimensions 5-levels questionnaire; KDQOL-SF, Kidney Disease Quality of Life-Short Form; MNA-SF, Mini nutritional assessment—short form; MUST, malnutrition universal screening tool; NRS2002, Nutrition risk screening 2002; SF-36, 36-item Short-Form Health Survey; SNAQ, Simplified nutritional appetite questionnaire.

## Data Availability

The data that support the findings of this study are available from the corresponding author upon reasonable request.

## Supplementary Materials

The following supporting information can be downloaded at: https://www.mdpi.com/article/10.3390/nu15030612/s1. Table S1: Analysis of quantitative and qualitative agreement between surveys by topic. Supplementary Information S1: Search terms used in literature review. Supplemental information S2: Definitions used in GRADE evidence profile tables. Supplemental information S3: List of Delphi participants
