# Supplementary material for: Expert Consensus on Morphofunctional Assessment in Disease-Related Malnutrition. Grade Review and Delphi Study
Source: Nutrients. 2023 Jan 25;15(3):612. doi: 10.3390/nu15030612 (PMC9920608; doi:10.3390/nu15030612)
Supplement: Supplementary file 1 [file nutrients-15-00612-s001.zip › Supplementary Information S1_22_05_2022.pdf]

SUPPLEMENTARY INFORMATION S1

Search terms used in literature review

Search terms for topic "food intake"

Prognosis/Narrow[filter] AND (Prognosis/Broad[filter] AND (("malnutrition"[MeSH Terms] OR "malnutrition"[All Fields]) AND ("nutritional assessment"[All Fields] OR "eating assessment"[All Fields] OR "intake"[All Fields]) AND (("mortality"[Subheading] OR "mortality"[All Fields] OR "mortality"[MeSH Terms]) OR "length of stay"[All Fields] OR "quality of life"[All Fields] OR ("complications"[Subheading] OR "complications"[All Fields]))) AND "humans"[MeSH Terms] AND (English[lang] OR Spanish[lang]) AND "adult"[MeSH Terms] AND ((Clinical Study[ptyp] OR Clinical Trial[ptyp] OR Comparative Study[ptyp] OR Controlled Clinical Trial[ptyp] OR Meta-Analysis[ptyp] OR Multicenter Study[ptyp] OR Observational Study[ptyp] OR Randomized Controlled Trial[ptyp] OR systematic[sb]) AND "humans"[MeSH Terms] AND "adult"[MeSH Terms])) AND ((Clinical Study[ptyp] OR Clinical Trial[ptyp] OR Comparative Study[ptyp] OR Controlled Clinical Trial[ptyp] OR Meta-Analysis[ptyp] OR Multicenter Study[ptyp] OR Observational Study[ptyp] OR Randomized Controlled Trial[ptyp] OR systematic[sb]) AND "humans"[MeSH Terms] AND "adult"[MeSH Terms])

Search terms for topic "nutrient assimilation"

Prognosis/Broad[filter] AND (("malnutrition"[MeSH Terms] OR "malnutrition"[All Fields]) AND ("intestinal absorption"[All Fields] OR "digestion"[All Fields]) AND (("mortality"[Subheading] OR "mortality"[All Fields] OR "mortality"[MeSH Terms]) OR "length of stay"[All Fields] OR "quality

of life"[All Fields] OR ("complications"[Subheading] OR "complications"[All Fields]))) AND ("humans"[MeSH Terms] AND (English[lang] OR Spanish[lang]) AND "adult"[MeSH Terms])

<u>Search terms for topic “anthropometry”</u>

(Prognosis/Broad[filter]) AND malnutrition AND (mid arm circumference OR skinfold thickness OR calf circumference) AND (mortality OR complications OR length of stay OR quality of life) NOT obesity AND Humans[Mesh] AND adult[MeSH]

<u>Search terms for topic “biochemical analysis”</u>

("malnutrition"[MeSH Terms] OR "malnutrition"[All Fields]) AND ("albumin"[All Fields] OR "prealbumin"[All Fields] OR "transthyretin"[All Fields] OR"c-reactive protein"[MeSH Terms] OR ("c-reactive"[All Fields] AND "protein"[All Fields]) OR "c-reactive protein"[All Fields] OR "c reactive protein"[All Fields]) AND (("mortality"[Subheading] OR "mortality"[All Fields] OR "mortality"[MeSH Terms]) OR ("complications"[Subheading] OR "complications"[All Fields]) OR ("postoperative period"[MeSH Terms] OR ("postoperative"[All Fields] AND "period"[All Fields]) OR "postoperative period"[All Fields] OR ("post"[All Fields] AND "operative"[All Fields]) OR "postoperative"[All Fields]) AND ("complications"[Subheading] OR "complications"[All Fields]))

<u>Search terms for topic “dynamometry”</u>

("malnutrition"[MeSH Terms] OR "malnutrition"[All Fields]) AND ("hand grip strength"[All Fields] OR "GRIP DYNAMOMETER"[All Fields] OR "HAND GRIP"[All Fields]) AND (("mortality"[Subheading] OR "mortality"[All Fields] OR "mortality"[MeSH Terms]) OR "lenght of

stay"[All Fields] OR "quality of life"[All Fields] OR ("complications"[Subheading] OR "complications"[All Fields])) AND ("humans"[MeSH Terms] AND "adult"[MeSH Terms]) AND (("0001/01/01"[PDAT] : "2019/02/05"[PDAT]) AND "humans"[MeSH Terms] AND "adult"[MeSH Terms])

Search terms for topic "phase angle"

("malnutrition"[MeSH Terms] OR "malnutrition"[All Fields]) AND (bioimpedance[All Fields] OR "bioelectrical impedance"[All Fields] OR "phase angle"[All Fields]) AND (("mortality"[Subheading] OR "mortality"[All Fields] OR "mortality"[MeSH Terms]) OR "length of stay"[All Fields] OR "quality of life"[All Fields] OR ("complications"[Subheading] OR "complications"[All Fields]))

Search terms for topic "muscle imaging"

Search terms used in PubMed: ("malnutrition" AND "tomography, x ray computed" AND ("mortality" OR "length of stay" OR "quality of life" OR "complications")) AND (("0001/01/01"[Date - Publication] : "2019/02/05"[Date - Publication]))

Search terms used in PubMed: ("malnutrition"[MeSH Terms] OR "malnutrition"[All Fields]) AND ("Ultrasonography"[All Fields] OR "Ultrasonography"[MeSH Terms]) AND ("mortality"[All Fields] OR "length of stay"[All Fields] OR "quality of life"[All Fields] OR ("complications"[Subheading] OR "complications"[All Fields])) AND ((clinicalstudy[Filter] OR clinicaltrial[Filter] OR controlledclinicaltrial[Filter] OR meta-analysis[Filter] OR multicenterstudy[Filter] OR

observationalstudy[Filter] OR randomizedcontrolledtrial[Filter] OR review[Filter] OR systematicreview[Filter]) AND ("0001/01/01"[PDAT] : "2019/02/05"[PDAT])

Search terms used in Embase: ('deficient nutrition'/exp OR 'deficient nutrition' OR 'malnourishment'/exp OR 'malnourishment' OR 'malnutrition'/exp OR 'malnutrition' OR 'severe acute malnutrition'/exp OR 'severe acute malnutrition' OR 'underfeeding'/exp OR 'underfeeding' OR 'undernourishment'/exp OR 'undernourishment' OR 'undernutrition'/exp OR 'undernutrition') AND ('elliptical tomography'/exp OR 'elliptical tomography' OR 'laminagraphy'/exp OR 'laminagraphy' OR 'laminography'/exp OR 'laminography' OR 'narrow angle zonography'/exp OR 'narrow angle zonography' OR 'planigraphy'/exp OR 'planigraphy' OR 'planography'/exp OR 'planography' OR 'planygraphy'/exp OR 'planygraphy' OR 'polytomography'/exp OR 'polytomography' OR 'radiotomography'/exp OR 'radiotomography' OR 'tomography'/exp OR 'tomography' OR 'transverse section imaging'/exp OR 'transverse section imaging' OR 'zonography'/exp OR 'zonography') AND ('muscle'/exp OR 'muscle' OR 'muscle fraction'/exp OR 'muscle fraction' OR 'muscle group'/exp OR 'muscle group' OR 'muscles'/exp OR 'muscles' OR 'muscular system'/exp OR 'muscular system' OR 'musculature'/exp OR 'musculature' OR 'musculi'/exp OR 'musculi' OR 'musculus'/exp OR 'musculus') AND ('excess mortality'/exp OR 'excess mortality' OR 'mortality'/exp OR 'mortality' OR 'mortality model'/exp OR 'mortality model' OR 'length of stay'/exp OR 'length of stay' OR 'complication'/exp OR 'complication' OR 'complications'/exp OR 'complications' OR 'quality of life'/exp OR 'quality of life') AND [humans]/lim AND [<1966-2019]/py

Search terms used in Embase: ('deficient nutrition'/exp OR 'deficient nutrition' OR 'malnourishment'/exp OR 'malnourishment' OR 'malnutrition'/exp OR 'malnutrition' OR 'severe

acute malnutrition'/exp OR 'severe acute malnutrition' OR 'underfeeding'/exp OR 'underfeeding' OR 'undernourishment'/exp OR 'undernourishment' OR 'undernutrition'/exp OR 'undernutrition') AND ('doptone'/exp OR 'doptone' OR 'duplex echography'/exp OR 'duplex echography' OR 'echogram'/exp OR 'echogram' OR 'echography'/exp OR 'echography' OR 'echoscopy'/exp OR 'echoscopy' OR 'echosound'/exp OR 'echosound' OR 'high resolution echography'/exp OR 'high resolution echography' OR 'scanning, ultrasonic'/exp OR 'scanning, ultrasonic' OR 'sonogram'/exp OR 'sonogram' OR 'sonography'/exp OR 'sonography' OR 'ultrasonic detection'/exp OR 'ultrasonic detection' OR 'ultrasonic diagnosis'/exp OR 'ultrasonic diagnosis' OR 'ultrasonic echo'/exp OR 'ultrasonic echo' OR 'ultrasonic examination'/exp OR 'ultrasonic examination' OR 'ultrasonic scanning'/exp OR 'ultrasonic scanning' OR 'ultrasonic scintillation'/exp OR 'ultrasonic scintillation' OR 'ultrasonography'/exp OR 'ultrasonography' OR 'ultrasound diagnosis'/exp OR 'ultrasound diagnosis' OR 'ultrasound scanning'/exp OR 'ultrasound scanning') AND ('muscle'/exp OR 'muscle' OR 'muscle fraction'/exp OR 'muscle fraction' OR 'muscle group'/exp OR 'muscle group' OR 'muscles'/exp OR 'muscles' OR 'muscular system'/exp OR 'muscular system' OR 'musculature'/exp OR 'musculature' OR 'musculi'/exp OR 'musculi' OR 'musculus'/exp OR 'musculus') AND ('excess mortality'/exp OR 'excess mortality' OR 'mortality'/exp OR 'mortality' OR 'mortality model'/exp OR 'mortality model' OR 'length of stay'/exp OR 'length of stay' OR 'hrql'/exp OR 'hrql' OR 'health related quality of life'/exp OR 'health related quality of life' OR 'life quality'/exp OR 'life quality' OR 'quality of life'/exp OR 'quality of life' OR 'complication'/exp OR 'complication' OR 'complications'/exp OR 'complications') AND [humans]/lim AND [<1966-2019]/py

Search terms for topic “functional status and quality of life”

("malnutrition"[MeSH Terms] OR "malnutrition"[All Fields]) AND ("Barthel"[All Fields] OR "timed up and go" OR "chair stand test" OR "SPPB" OR "Katz index"[All Fields] OR (Lawton[All Fields] AND Brody[All Fields]) OR "ECOG"[All Fields] OR "karnofski index"[All Fields] OR “KPS” [All Fields] OR "HRQOL"[All Fields] OR "IADL"[All Fields]) OR "walking test"[All Fields] AND (("mortality"[Subheading] OR "mortality"[All Fields] OR "mortality"[MeSH Terms]) OR "Lenght of stay"[All Fields] OR "complications"[All Fields]) AND ((Clinical Trial[ptyp] OR Meta-Analysis[ptyp] OR Observational Study[ptyp] OR systematic[sb]) AND "humans"[MeSH Terms] AND (English[lang] OR Spanish[lang]) AND "adult"[MeSH Terms])
