# Supplementary material for: Expert Consensus on Morphofunctional Assessment in Disease-Related Malnutrition. Grade Review and Delphi Study
Source: Nutrients. 2023 Jan 25;15(3):612. doi: 10.3390/nu15030612 (PMC9920608; doi:10.3390/nu15030612)
Supplement: Supplementary file 1 [file nutrients-15-00612-s001.zip › Supplementary Information S2_GRADE Tables_FINAL.pdf]

Supplemental information S2

Definitions used in GRADE evidence profile tables

Evidence tables are used to summarize the body of evidence, the judgments about the quality of the evidence, key results, and importance.

**No. of studies**	**Certainty assessment**						**Effect**			**Certainty**	**Importance**
	**Study design**	**Risk of bias**	**Inconsistency**	**Indirectness**	**Imprecision**	**Other considerations**	**No. of events**	**No. of individuals**	**HR (95% CI)**		
Outcome											
1 [1]	Study design plays a critical role in when judging the quality of evidence. With GRADE, randomized trials without important limitations provide high quality of evidence, and observational studies without special strengths or important limitations provide low quality of evidence.	Limitations in the study design and execution that may bias the estimated treatment effect. Some examples include lack of blinding, flawed measurement of exposure and outcome, and incomplete accounting of patients and outcome events.	Unexplained heterogeneity of results. The quality of the evidence is downgraded if the study does not identify a plausible explanation for heterogeneity of results.	Direct evidence is research that directly compares the interventions of interest, delivered to the populations of interest, and measures the outcomes that are important to patients.	Results are imprecise when studies include relatively few patients and few events, resulting in a wide confidence interval around the estimate of the effect. Optimal information size is also taken into account.	Publication bias is a systematic under-estimation or an over-estimation of the underlying beneficial or harmful effect due to the selective publication of studies.	The number of events plays a role in the estimated magnitude of the effect.	The number of individuals plays a role in the estimated magnitude of the effect.	HR measures the effect produced by a survival analysis, representing the increased risk with which one group is likely to experience the outcome of interest.	Certainty is an assessment of how good an indication the research provides of the likely effect. Ratings: Very low / Low / Moderate / High	Importance for decision-making is used to categorize outcomes intro three categories: Critical / Important but not critical / Of limited importance

Topic 1: Food intake and nutrient assimilation

Author(s): Juan José López Gómez

Question: Assessment of altered food intake in patients with disease-related malnutrition (or at risk of malnutrition) during follow-up.

Setting: General population is not included. Chronic or acute illnesses are included.

No. of studies	Certainty assessment						Effect			Certainty	Importance
	Study design	Risk of bias	Inconsistency	Indirectness	Imprecision	Other considerations	No. of events	No. of individuals	HR (95% CI)		
Mean length of stay in hospitalized elderly (evaluated with: mean days of stay)											
1 [1]	observational studies	serious [a]	not serious	serious [a]	serious [b]	Strong association. All possible residual confounding factors could reduce the observed effect	78	172	3.25 (1.16 to 9.13)	⊕⊕⊕◯ MODERATE	CRITICAL
Evaluation of mean length of stay and readmission (evaluated with: patient's medical history)											
1 [2]	observational studies	serious [c]	not serious	serious [d]	very serious [e]	all possible residual confounding factors could reduce the observed effect		57		⊕◯◯◯ VERY LOW	IMPORTANT
Mean length of stay (evaluated with: days of hospitalization)											
1 [3]	observational studies	serious [f]	not serious	serious [g]	not serious	all possible residual confounding factors could reduce the observed effect		505		⊕⊕⊕◯ MODERATE	IMPORTANT
Mean length of stay in elderly diabetic patients (evaluated with: mean length of stay)											
1 [4]	observational studies	serious [h]	not serious	serious [a]	not serious	all possible residual confounding factors could reduce the observed effect		1090	1.15 (1.084 to 1.219)	⊕⊕⊕◯ MODERATE	IMPORTANT
Mean length of stay in patients with colorectal surgery following enhanced recovery after surgery (ERAS) protocol (evaluated with: days of hospitalization).											
1 [5]	observational studies	serious [i]	not serious	not serious	serious [b]	strong association		115	4.4 (-6.8 to -2)	⊕⊕⊕◯ MODERATE	IMPORTANT
Mean length of stay greater than 7 days in middle-aged hospitalized patients (evaluated with: medical history).											
1 [6]	observational studies	not serious	not serious	not serious	not serious	none		799	1.56 (1.12 to 2.18)	⊕⊕⊕⊕ HIGH	CRITICAL
Major complications in surgical patients with pancreatic cancer (evaluated with: death registry).											
1 [7]	observational studies	serious [j]	serious [k]	serious [a]	serious [l]	all possible residual confounding factors could reduce the observed effect	61	279	2.64 (0.71 to 9.83)	⊕◯◯◯ VERY LOW	LIMITED IMPORTANCE

No. of studies	Certainty assessment						Effect			Certainty	Importance
	Study design	Risk of bias	Inconsistency	Indirectness	Imprecision	Other considerations	No. of events	No. of individuals	HR (95% CI)		
Postoperative complications in patients with colorectal cancer (evaluated with: death registry).											
1 [8]	observational studies	serious [m]	not serious	serious [g]	serious	all possible residual confounding factors could reduce the observed effect	18	70	66.7 per 100	⊕⊕◯◯ LOW	IMPORTANT
Complications in patients after scheduled cardiac surgery (evaluated with: medical history).											
1 [9]	observational studies	serious [a]	not serious	serious [a]	serious [n]	all possible residual confounding factors could reduce the observed effect		1192	1.37 (0.7 to 2.7)	⊕⊕◯◯ LOW	IMPORTANT
Functional capacity after hip fracture (assessed with: Functional Independent Measurement Instrument)											
1 [10]	observational studies	serious [o]	not serious	serious [p]	serious [q]	all possible residual confounding factors could reduce the observed effect		204	-0.86	⊕⊕◯◯ LOW	IMPORTANT
Overall mortality in hemodialysis patients (evaluated with: death registry)											
1 [11]	case series (uncontrolled single-arm studies)	serious [r]	not serious	serious [s]	serious [b]	strong association. All possible residual confounding factors could reduce the observed effect	26	75	2.90(1.17 to 7.16)	⊕⊕⊕◯ MODERATE	IMPORTANT
Mortality in hemodialysis patients (evaluated with: death registry).											
1 [12]	observational studies	serious [t]	not serious	serious [u]	serious [v]	all possible residual confounding factors could reduce the observed effect	183	489	1.56	⊕⊕◯◯ LOW	IMPORTANT
Postoperative mortality in patients with critical limb ischemia (evaluated with: clinical registry).											
1 [13]	observational studies	serious [w]	not serious	serious [a]	serious [b]	strong association. All possible residual confounding factors could reduce the observed effect	14	106	6.1 (1.6 to 23.7)	⊕⊕⊕◯ MODERATE	LIMITED IMPORTANCE
Overall mortality in patients with heart failure (evaluated with: death registry).											
11 [14]	observational studies	not serious	not serious	not serious	serious [x]	strong association		4300	4.06 (2.41 to 6.84)	⊕⊕⊕⊕ HIGH	CRITICAL
Mortality in patients after hip fracture (evaluated with: death registry).											
1 [15]	observational studies	serious [a]	not serious	serious [a]	serious [b]	strong association. All possible residual confounding factors could reduce the observed effect	333	594	7.66 (4.18 to 14)	⊕⊕⊕◯ MODERATE	IMPORTANT

No. of studies	Certainty assessment						Effect			Certainty	Importance
	Study design	Risk of bias	Inconsistency	Indirectness	Imprecision	Other considerations	No. of events	No. of individuals	HR (95% CI)		
Postoperative mortality in patients with scheduled cardiac surgery (evaluated with: death registry).											
1 [16]	observational studies	serious [a]	not serious	serious [a]	not serious	all possible residual confounding factors could reduce the observed effect	33	1193	1.6 (1.1 to 2.2)	⊕⊕⊕◯ MODERATE	IMPORTANT
Mortality at one year in elderly with home care (evaluated with: mortality registry).											
1 [17]	observational studies	serious [y]	not serious	serious [a]	serious [b]	strong association all possible residual confounding factors could reduce the observed effect	61	309	8.75 (2.45 to 31.18)	⊕⊕⊕◯ MODERATE	IMPORTANT
Mortality at 4 years in the elderly (evaluated with: mortality registry).											
1 [18]	observational studies	serious [z]	not serious	serious [a]	not serious	strong association. All possible residual confounding factors could reduce the observed effect	578	2892	3.26 (2.31 to 4.6)	⊕⊕⊕⊕ HIGH	CRITICAL
Mortality in the elderly after admission (evaluated with: nutritional status and mortality)											
1 [10]	observational studies	serious [aa]	not serious	serious [a]	not serious	all possible residual confounding factors could reduce the observed effect	82	204	1.85 (1.22 to 2.81)	⊕⊕⊕◯ MODERATE	CRITICAL
Mortality in elderly patients after admission (evaluated with: death registry).											
1 [1]	observational studies	serious [ab]	not serious	serious [a]	serious	all possible residual confounding factors could reduce the observed effect	28	172	3.25 (1.16 to 9.13)	⊕⊕◯◯ LOW	CRITICAL
Mortality in elderly hospitalized patients (evaluated with: death registry).											
1 [19]	observational studies	serious [ac]	serious [ad]	serious [a]	serious [ae]	all possible residual confounding factors could reduce the observed effect	41	131	5.49 (0.48 to 8.92)	⊕◯◯◯ VERY LOW	IMPORTANT
Mortality in elderly hospitalized patients (evaluated with: spdeath registry).											
1 [4]	observational studies	serious [i]	not serious	serious [a]	not serious [af]	all possible residual confounding factors could reduce the observed effect	44	1090	0.895 (0.814 to 0.985)	⊕⊕⊕◯ MODERATE	CRITICAL
Mortality in elderly patients after admission (evaluated with: death registry).											
1 [20]	observational studies	serious [ag]	not serious	serious [a]	not serious	all possible residual confounding factors could reduce the observed effect	90	225	1.85 (1.22 to 2.81)	⊕⊕⊕◯ MODERATE	CRITICAL

Explanations

a. Mini Nutritional Assessment (MNA) used to assess food intake.

b. Very wide 95% CI.

c. Small sample size. Use of body mass index (BMI) and ICD-10-AM as gold-standard.

d. Use of MNA and subjective global assessment (SGA) to assess food intake.

e. Measures sensitivity and specificity to detect admission–readmission, not hazard ratio (HR).

f. Evaluation of SGA as a whole. Limited to a given population (not multicenter).

g. Use of SGA.

h. Lack of use of a specific food intake assessment tool. Very specific patient sample (elderly with diabetes).

i. Assessment of food intake compared to a protocol, not to routine intake. The main objective is to compare two interventions.

j. Only one center. 25% patient loss (refusal to participate).

k. Not comparable with similar studies.

l. Not significant HR.

m. Small sample size. No separate assessment of oral food intake.

n. Wide range.

o. MNA evaluates food intake integrated with other parameters. Significant differences in some confounding factors between malnourished and well nourished patients. Study limited to the acute phase.

p. Use of the MNA-short form (MNA-SF).

q. No 95% CI for OR.

r. Small sample size. Low reproducibility outside of dialysis patients. The nutritional method does not assess food intake separately.

s. Uses Malnutrition-Inflammation Score (MIS) (food intake is one of its 4 items).

t. Very specific population (hemodialysis patients). Confounding factors related to mortality, albumin, and inflammatory parameters within MIS. Non-prospective study and use of calculated parameters.

u. Use of MIS and SGA nutritional assessment tests.

v. Nutritional assessment test

w. MNA was not used in all patients.

x. Use of MNA (and, therefore, indirect food intake assessment). The main objective was not the evaluation of food intake tools.

y. Variability in the use of functionality scales and MNA, given the study was multicentric.

z. No separate assessment of food intake. Non-homogeneous patient sample with 2/3 of the total aged 60–75 years.

aa. Population with many illnesses. Possible confounding factors between deceased and non-deceased.

ab. Small sample size. Patients with language barriers and cognitive impairment were excluded.

ac. Design. No separate food intake assessment was conducted, only in the context of MNA. Small sample size.

ad. The result is not consistent with similar studies.

ae. Result was not significant.

af. MNA is used quantitatively.

ag. Population with many illnesses. Possible confounding factors between deceased and non-deceased patients.

References

1. Performance of nutritional screening tools in predicting poor six-month outcome in hospitalised older patients. Asia Pac J Clin Nutr; 2014.

2. Malnutrition in Geriatric Rehabilitation: Prevalence, Patient Outcomes, and Criterion Validity of the Scored Patient-Generated Subjective Global Assessment and the Mini Nutritional Assessment. J Acad Nutr Diet; 2016.

3. Validation of the Chinese version of the Subjective Global Assessment scale of nutritional status in a sample of patients with gastrointestinal cancer. Int J Nurs Stud; 2010.

4. Malnutrition prevalence in hospitalized elderly diabetic patients. Nutr Hosp; 2013.

5. Protein intakes are associated with reduced length of stay: a comparison between Enhanced Recovery After Surgery (ERAS) and conventional care after elective colorectal surgery. Am J Clin Nutr; 2017.

6. Nutritional assessment: comparison of clinical assessment and objective variables for the prediction of length of hospital stay and readmission. Am J Clin Nutr; 2015.

7. Prospective trial to evaluate the prognostic value of different nutritional assessment scores in pancreatic surgery (NURIMAS Pancreas). Br J Surg; 2017.

8. Relationship between nutritional status and the Glasgow Prognostic Score in patients with colorectal cancer. Nutrition; 2013.

9. Evaluation of nutritional screening tools for patients scheduled for cardiac surgery. Nutrition; 2013.

10. Pre-fracture nutritional status is predictive of functional status at discharge during the acute phase with hip fracture patients: A multicenter prospective cohort study. Clin Nutr; 2017.

11. Comparison analysis of nutritional scores for serial monitoring of nutritional status in hemodyalisis patients. Clin J Am Soc Nephrol; 2013.

12. A Comparison of 8 Nutrition-Related Tests to predict Mortality in Hemodyalisis Patients. J Ren Nutr; 2015.

13. Impact of Nutritional State on Critical Limb Ischemia Early Outcomes (DENUCRITICC Study). Ann Vasc Surg; 2017.

14. Review of nutritional screening and assessment tools and clinical outcomes in heart failure. Eur J Clin Invest; 2016.

15. Comparison of the Mini-Nutritional Assessment short and long form and serum albumin as prognostic indicators of hip fracture outcomes. Injury; 2017.

16. Prognostic value of nutritional screening tools for patients scheduled for cardiac surgery. Interact Cardiovasc Thorac Surg; 2013.

17. Prognostic differences of the Mini Nutritional Assessment short form and long form in relation to 1-year functional decline and mortality in community-dwelling older adults receiving home care. J Am Geriatr Soc; 2014.

18. The short-form mini-nutritional assessment is as effective as the full-mini nutritional assessment in predicting follow-up 4-year mortality in elderly Taiwanese. J Nutr Health Aging; 2013.

19. The validity of Geriatric Nutrition Risk Index: simple tool for prediction of nutritional-related complication of hospitalized elderly patients. Comparison with Mini Nutritional Assessment. Clin Nutr; 2014.

20. Nutritional predictors of mortality after discharge in elderly patients on a medical ward. Eur J Clin Invest; 2016.

Topic 2: Anthropometry

Author(s): Irene Breton

Question: Progression of acute or chronic diseases, in terms of mortality, mean length of stay, complications, or quality of life in patients with short-, medium- and long-term altered anthropometry.

Setting: General population is not included.

No. of studies	Certainty assessment						Effect			Certainty	Importance
	Study design	Risk of bias	Inconsistency	Indirectness	Imprecision	Other considerations	No. of events	No. of individuals	Rate (95% CI)		
Overall mortality in hospitalized patients with liver cirrhosis (follow-up: mean 2 years; evaluated with: triceps skinfold (TSF) and mid-arm muscle circumference [MAMC])											
4 [1,2,3,4]	observational studies	not serious	serious [a]	not serious	serious [b]	all possible residual confounding factors could reduce the observed effect	1. 212 hospitalized patients with cirrhosis, with follow-up up to 2 years. Severe (TSF or MAMC <5th percentile) or moderate (<10th percentile) malnutrition were independent predictors of mortality, assessed by Cox multivariate regression analysis, and both improved Child–Pugh prediction. MAMC had a stronger prognostic power than TSF.<br><br>2. 130 outpatients/hospitalized patients, 19% overall mortality at 2 years. Malnutrition diagnosed by TSF is associated with higher mortality (OR 15.2 p<0.001).<br><br>3. Evaluates 184 patients with cirrhosis, does not define whether they are hospitalized. Defines malnutrition as TSF<30–50% of the reference value. Child–Pugh B/C patients with malnutrition presented higher mortality at 1 and 2 years (p<0.001).<br><br>4. 120 hospitalized patients with cirrhosis. Patients with MAMC and TSF <P5 have significantly higher mortality at 3, 6, 12 and 24 months (Kaplan-Meier).			⨁⨁⨁◯ MODERATE	CRITICAL
Overall mortality in hemodialysis patients (follow-up: 1–5 years; evaluated with: mid-arm circumference [MAC], MAMC, TSF).											
6 [5,6,7,8,9,10]	observational studies	not serious	not serious	not serious	not serious	none	5. 100 patients, 7% mortality at one year. Patients who died had a lower TSF: median (CI range), Mann-Whitney test: median 9 (7–11) vs 14 (10–19), p=0.021. MIS is a strong predictor of survival, with a cut-off value of 6.5.			⨁⨁⨁⨁ HIGH	CRITICAL

No. of studies	Certainty assessment						Effect			Certainty	Importance
	Study design	Risk of bias	Inconsistency	Indirectness	Imprecision	Other considerations	No. of events	No. of individuals	Rate (95% CI)		
							6. 242 patients, follow-up 1–132 months. Overall mortality risk factors (Cox univariable proportional hazard model) HR(CI): TSF 0.95 (0.92–0.98) p<0.01; MAC 0.91 (0.87–0.96) p<0.01; MAMC 0.089 (0.83–0.95) p<0.01.<br><br>7. 792 patients. MAMC in the lower two quartiles had lower survival (Kaplan-Meier p<0.01). Maintained after adjusting for case-mix, inflammatory markers and malnutrition–inflammation–cachexia syndrome.<br><br>8. 128 patients. Patients who died had lower MAMC. MAMC was a predictor of mortality (Kaplan–Meier).<br><br>9. 761 patients (446 followed-up at one year), 15% mortality. MAMC was significantly lower in patients who died 21.5 (3.0) vs 22.2 (3.4). p=0.041. No differences were observed in TSF (Student's t-test).<br><br>10. 28 patients, mortality at one year 39.3%. CMB was not related to mortality (<cut-off value 61.1% higher than cut-off value 60.0 p=0.311 chi-squared).				
Overall mortality in hospitalized patients (follow-up: 1– 5 years; evaluated with: MAMC and TSF).											
2 [11,12]	observational studies	serious [c]	not serious	not serious	serious [c]	all possible residual confounding factors could reduce the observed effect	11. 445 patients >65 years admitted with acute medical or surgical illness. MAMC quartiles at admission were associated to mortality, HR 0.73 (0.56–0.97) p=0.028.<br><br>12. 644 patients admitted for gastroenterological disease or liver disease. The decrease in MAMC and TSF was related to lower survival at 5 years (p<0.001 and p<005, respectively).			⊕⊕⊕◯ MODERATE	CRITICAL
Overall mortality in patients admitted to a geriatric rehabilitation unit (follow-up: 1–4.5 years; evaluated with: MAMC, TSF).											
2 [13,14]	observational studies	not serious	serious [d]	not serious	not serious	none	13. 219 patients. MAMC and TSF were associated with higher mortality at 4.5 years. MAMC: prevalence 35.9% HR 2.1; 95% CI			⊕⊕⊕◯ MODERATE	CRITICAL

No. of studies	Certainty assessment						Effect			Certainty	Importance
	Study design	Risk of bias	Inconsistency	Indirectness	Imprecision	Other considerations	No. of events	No. of individuals	Rate (95% CI)		
							(1.5–2.9) p<0.0001. TSF prevalence 13.7% HR 2.0; 95% CI (1.4–3.1) p<0.001.<br><br>14. 110 patients. MAMC and TSF were associated with mortality at 1 year in univariate analysis (p<0.08) but not in multivariate analysis.				
Overall mortality in patients with chronic obstructive pulmonary disease (COPD) (evaluated with: MAC, calf circumference [CC], arm muscle area)											
2 [15,16]	observational studies	serious [e]	not serious	not serious	not serious	none	15. 96 male patients with mean age 69±9 years. Mid-arm muscle area ≤25th percentile was associated with higher mortality at 3 years in multivariate analysis.<br><br>16. 104 patients, 94.2% male, mean age 74.2± 6.9. MAC <23.5 cm and CC <30 cm were associated with higher mortality: HR 3.09 (95% CI 1.30–7.38; 0<0.001 and HR 4.40 (95% CI 1.82–10.63); p<0.001 respectively.			⨁⨁⨁◯ MODERATE	CRITICAL
Overall mortality in cancer patients (follow-up: 1–255 weeks; evaluated with: TSF, MAC, arm muscle area, CC)											
3 [17,18,19]	observational studies	not serious	serious [d]	not serious	not serious	none	17. 334 patients (58.4% women) undergoing palliative treatment for cancer. Decreased mid-arm muscle area (<32 cm^2 in men and <18 cm^2 in women) was associated with poorer survival: HR 1.57 (95% CI 1.12–2.18; p=0.007); Decreased CC (<34 cm in men and <33 in women) was associated with poorer survival: HR 2.00 (95% CI 1.45–2.76) p<0.001.<br><br>18. 252 patients with diffuse large B-cell lymphoma treated with R-CHOP. MAMC is associated with overall survival, but not with disease-free survival. In multivariate analysis, no relationship between anthropometric parameters and survival was observed.<br><br>19. 338 patients with non-small cell lung cancer. Kaplan-Meier survival analysis: TSF >10 mm (1st quartile), median survival was 46 weeks (95% CI 38–58); ≤5 mm (4th quartile), 22 weeks			⨁⨁⨁◯ MODERATE	CRITICAL

No. of studies	Certainty assessment						Effect			Certainty	Importance
	Study design	Risk of bias	Inconsistency	Indirectness	Imprecision	Other considerations	No. of events	No. of individuals	Rate (95% CI)		
							(95% CI 11–34) p<0.01. MAC >28 cm, (1st quartile), 44 weeks (95% CI 33–56); ≤23 cm (4th quartile) 30 weeks (23–37) p<0.01.				
Overall mortality in patients with HIV infection (follow-up: mean 6 months; evaluated with: MAC)											
1 [20]	observational studies	serious [f]	not serious	serious [g]	not serious	none	812 patients were evaluated, 41% male, age 32 years (28–40).<br>MAC was associated with mortality at 6 months. Continuous variable: with each 1-cm increase, multivariate analysis, HR 0.82 (95% CI 0.71–0.94).			⊕⊕◯◯ LOW	CRITICAL
Overall mortality in patients with traumatic and non-traumatic brain injury (follow-up: mean 5 months; evaluated with: TSF and MAMC)											
1 [21]	observational studies	serious [h]	not serious	not serious	serious	all possible residual confounding factors could reduce the observed effect	30 patients. No association was found between anthropometric measures and mortality at 5 months.			⊕⊕⊕◯ MODERATE	CRITICAL
Mortality at hospital discharge in critically ill patients (evaluated with: MAC, MAMC, TSF)											
2 [22,23]	observational studies	not serious	serious [i]	not serious	not serious	none	22. 1363 patients from 31 ICUs. Hospital stay 21.1 days (25.5). 21.4% mortality at discharge. MAMC was associated with mortality OR 0.95 95% CI (0.93–0.98) p<0.001. No association was observed with TSF: OR 1.01; 95% CI (0.99–1.02) p=0.3218.<br>23. 44 patients. 51% mortality. MAC <5th percentile was associated with higher mortality p=0.003. In multivariate logistic regression, MAC <15th percentile showed a significant association with mortality, P=0.03.			⊕⊕⊕◯ MODERATE	IMPORTANT
In-hospital mortality in patients with liver cirrhosis (evaluated with: MAC)											
1 [24]	observational studies	not serious	not serious [j]	not serious	not serious [h]	none	prospective study of 60 patients. Malnutrition defined by anthropometry (MAC <5th percentile) was not associated			⊕⊕⊕⊕ HIGH	IMPORTANT

No. of studies	Certainty assessment						Effect			Certainty	Importance
	Study design	Risk of bias	Inconsistency	Indirectness	Imprecision	Other considerations	No. of events	No. of individuals	Rate (95% CI)		
							with increased mortality; relative risk (RR) 1.27 (0.51–3.2) p=0.417.				
In-hospital mortality in acute patients (follow-up: 15–60 days; evaluated with: MAMC, TSF)											
4 [25,26,27,28]	observational studies	not serious	serious [d]	not serious	not serious	none	25. 1561 hospitalized patients with acute illness. MAMC <25 cm is related to higher in-hospital, age-adjusted mortality > 60 years; OR 4.18 (2.33–7.47) p<0.001.<br><br>26. 396 hospitalized patients with acute illness. Malnutrition was diagnosed with MAMC. Poorer outcome was. associated with malnutrition: significant differences in the distribution of patients into malnutrition categories (deceased or alive at discharge), Chi-square test, p=0.03.<br><br>27. 318 patients. MAC <20 cm in men and <19 cm in women was associated with in-hospital mortality, adjusted analysis HR 1.8 (95% CI 0.98–3.4; p=0.06) and increased 30-day mortality: HR 1.6 (95% CI 1.0–2.3 p=0.048).<br><br>28. 324 patients >70 years admitted for medical illness. MAC was lower in patients who died within 15 days: 26.9 (3.8) cm vs 23.5 (4.0) cm p<0.01, Student's t-test.			⨁⨁⨁◯ MODERATE	IMPORTANT
In-hospital mortality in patients with obstructive jaundice (assessed with: TSF, MAMC).											
1 [29]	observational studies	serious	serious [k]	not serious	serious [l]	all possible residual confounding factors could reduce the observed effect	39 patients with obstructive jaundice of different etiology. No relationship was found between anthropometry and hospital mortality. Mortality in patients with TSF <15th percentile 0/10 vs 5/28 in patients with TSF > 15th percentile p=0.298. Mortality in patients with MAMC <15th percentile 2/12 vs 3/26 when higher, p=0.643.			⨁⨁◯◯ LOW	IMPORTANT
Major complications in critically ill patients (evaluated with: MAC, MAMC, TSF)											
1 [23]	observational studies	not serious	not serious	serious [m]	serious [m]	all possible residual confounding	44 critically ill patients. 51% mortality. MAC <15th percentile was associated with an increased			⨁⨁⨁◯ MODERATE	IMPORTANT

No. of studies	Certainty assessment						Effect			Certainty	Importance
	Study design	Risk of bias	Inconsistency	Indirectness	Imprecision	Other considerations	No. of events	No. of individuals	Rate (95% CI)		
						factors could reduce the observed effect	risk of complications (sepsis, multiorgan failure); multivariate logistic regression, p=0.03.				
Severe complications in hospitalized geriatric patients (medical/surgical) (evaluated with: MAC, suprailiac skinfold)											
1 [30]	observational studies	not serious	not serious	not serious	not serious	none	586 hospitalized geriatric patients, 6.3% presented a serious complication. MAC tertile was associated with risk of complications: 13.8% (lower tertile), 2.4% (middle tertile), 3.2% upper tertile, p<0.001. Suprailiac skinfold tertile was associated with risk of complications: 13.7%, 4.3%, 1.6% p<0.001. Other results: weight loss > 5% and BMI <22 kg/m^2 were related to increased risk of complications.			⊕⊕⊕⊕ HIGH	IMPORTANT
Pressure sores in patients with traumatic and non-traumatic brain injury (follow-up: mean 5 months; assessed with: TSF, MAMC).											
1 [21]	observational studies	serious	serious [n]	not serious	serious [h,o]	all possible residual confounding factors could reduce the observed effect	5 patients. Absolute values of CMMB were correlated to the degree of pressure sores; r=-0.511 p=0.005. No correlation was found with TSF: r=-0.316 p=0.095.			⊕⊕◯◯ LOW	IMPORTANT
Peritonitis in patients with continuous ambulatory peritoneal dialysis (CAPD) (follow-up: mean 6 months; assessed with: TSF, MAMC)											
1 [31]	observational studies	not serious	serious [h]	not serious	serious [h,o]	none	31 patients with CAPD. Absolute values of TSF were related to the number of cases of peritonitis within 6 months r=0.329, p<0.05. No relationship was observed with MAMC.			⊕⊕◯◯ LOW	IMPORTANT
Mean hospital stay in patients with obstructive jaundice (evaluated with: TSF, MAMC)											
1 [29]	observational studies	serious [h]	serious [k,p]	not serious	serious [q]	all possible residual confounding factors could reduce the observed effect	38 patients with obstructive jaundice of different etiology (neoplastic and non-neoplastic). Malnutrition defined as TSF or MAMC <15th percentile. Patients with malnutrition had a significantly higher mean length of stay: median 10 days vs 3 p=0.048			⊕⊕◯◯ LOW	IMPORTANT

No. of studies	Certainty assessment						Effect			Certainty	Importance
	Study design	Risk of bias	Inconsistency	Indirectness	Imprecision	Other considerations	No. of events	No. of individuals	Rate (95% CI)		
Mean hospital stay in patients with an acute illness (evaluated with: MAC, MAMC, TSF, CC)											
5 [25,26,32,33,34,35]	observational studies	not serious	serious [d,n]	serious [d]	not serious	all possible residual confounding factors could reduce the observed effect	25. 1561 hospitalized patients with acute illness. MAMC was not evaluated in 78 of patients. MAMC <25 cm predicted a prolonged mean stay, truncated at 21 days (R2=0.7%; p<0.01). BMI did not significantly predict mean length of stay.<br><br>26. 396 hospitalized patients with acute illness. MACM and TSF did not significantly predict mean length of stay (Kruskal-Wallis, multiple category comparison and mean length of stay, p=0.12 and 0.34, respectively).<br><br>32. 278 patients. Mean length of stay was longer in patients with MAC <25 cm: 17.29±13.23 days vs 14.16±12.52 days with MAC > 25 cm; p<0.05 (Mann-Whitney test). In women, MAC predicted mean stay > 12 days: OR 2.8 95% CI (1.3–6.1), p<0.05. TSF <70% predicted mean stay > 12 days: OR 3.1 (1.6–6.24), p<0.001.<br><br>34. 1033 medical and surgical patients, evaluable data for 733. Logistic regression. MAC was not related to an increased risk of hospitalization at 7 days: OR 0.98 (95% CI 0.96–1.91) p=0.1. CC showed a nearly significant relationship: OR 0.97 (95% CI 9.55–1.0) p=0.005.<br><br>35. 295 patients of Nutrition Day. MAC <21 cm and CC <31 cm were associated with an increased risk of hospitalization > 7 days: OR 6.52 (95% CI 2.91–18.55) p<0.001) and OR 2.51 (1.51–4.3).			⊕⊕⊕◯ MODERATE	IMPORTANT
Mean length of stay in hospitalized surgical patients (evaluated with: MAC, MAMC, TSF)											
1 [33]	observational studies	serious [r]	not serious	not serious	not serious	none	298 surgical patients. MAC and MAMC <15th percentile and <50th percentile predicted longer mean length of stay. Wilcoxon nonparametric test, p<0.001. No relationship was observed with TSF.			⊕⊕⊕◯ MODERATE	IMPORTANT

No. of studies	Certainty assessment						Effect			Certainty	Importance
	Study design	Risk of bias	Inconsistency	Indirectness	Imprecision	Other considerations	No. of events	No. of individuals	Rate (95% CI)		
Hospital readmission (follow-up: mean 30 days; evaluated with: CC, MAC)											
2 [34,36]	observational studies	not serious	serious [d]	not serious	serious [d]	none	34. 661 patients. MAC and CC were not associated with 30-day readmission. Univariate analysis. MAC: OR 0.98 (95% CI 0.95–1.01; p=0.2). CC: OR 0.99 (95% CI 0.96–1.02 p=0.6.<br>36. 161 patients admitted to internal medicine, 44% with decreased CC (<34 cm in men and <33 cm in women), was associated with a higher admission rate. Multivariate analysis adjusted for sex and age: OR 3.89 (95% CI 3.34–11.31; p=0.008.			⊕⊕◯◯ LOW	IMPORTANT
Quality of life in chronic hemodialysis patients (evaluated with: MAMC)											
1 [7]	observational studies	not serious	not serious	not serious	serious [s]	none	792 patients. MAMC was associated with the mental health scale of SF-36, p<0.01.			⊕⊕⊕◯ MODERATE	IMPORTANT

CC, calf circumference; MAC, mid-arm circumference; MAMC, mid-arm muscle circumference; TSF, triceps skinfold; R-CHOP, rituximab, cyclophosphamide, doxorubicin, vincristine, and prednisone.

Explanations

a. Studies conducted in different types of populations (hospitalized, outpatients) and in different clinical settings that do not rule out malnutrition that is not disease-related.

b. The cause of death (due to hepatocellular carcinoma or liver failure) has not been evaluated, which may be differently affected by malnutrition.

c. Patients with severe illness were excluded.

d. Results are inconsistent with those of other studies.

e. Most patients were male.

f. Patients for whom there was no tuberculosis data were excluded.

g. High percentage of patients lost to follow-up.

h. Small sample size.

i. Results are inconsistent results between the two studies with regard to the role of MAMC.

j. Results are variable in different studies; results are not always adequately described.

k. The effect of the etiology of jaundice on the mean length of stay was not evaluated.

l. The effect of the etiology on the mean length of stay was not evaluated.

m. Confounding factors are not defined; small sample size.

n. The mean length of stay was evaluated up to 21 days.

o. Gender was not taken into account when considering absolute anthropometry measures.

p. Data were not adequately described, only median length of stay was reported, without a range.

q. Imprecise data, only median values.

r. Bedridden patients were excluded.

s. Positive results only in one dimension of quality of life.

References:

1. Alberino F, Gatta A,Amodio P Merkel C,Pascoli L,Boffo G, L, Caregaro. Nutrition and Survival in Patients with Liver Cirrhosis. Nutrition; 2001.

2. Nunes G, Santos CA,Barosa R,Fonseca C,Barata AT,Fonseca J. Outcome and nutritional assessment of chronic liver disease patients using anthropometry and subjective global assessment. Arq Gastroenterol ; 2017.

3. Loguercio C, Sava E,Sicolo P,Castellano L,Narciso O. Nutritional Status and survival in patients with liver Cirrhosis: anthropometric evaluation . Minerva Gastroenterol Dietol; 1996.

4. Caregaro L Alberino F, Amodio P ,Merkel C, Bolognesi M, Angeli P,Gatta A:. Malnutrition in alcoholic and virus-related cirrhosis. Am J Clin Nutr; 1995.

5. Kara E, Sahutoglu T,Ahbap E,Sakaci T,Koc Y,Basturk T,Sevinc M,Akgol C,Unsal A. The predictive value of malnutrition – inflammation score on 1-year mortality in Turkish maintenance hemodialysis patients. Clinical Nephrology; 2016.

6. Stosovic M, Stanojevic M,Simic-Ogrizovic S,Jovanovic D,Djukanovic L. The predictive value of anthropometric parameters on mortality in haemodialysis patients. Nephrol Dial Transplant; 2011.

7. Noori N, Kopple JD,Kovesdy CP,Feroze U,Sim JJ, Murali SB,Luna A,Gomez M,Luna C,Bross R,Nissenson AR,Kalantar-Zadeh K. Mid-Arm Muscle Circumference and Quality of Life and Survival in Maintenance Hemodialysis Patients. Clin J Am Soc Nephrol; 2010.

8. Rashid Qureshi A, Alvestrand A, Divino-Filho Jc,Gutierrez A,Heimbu¨ Rger O,Blindholm B,Bergstrom J. Inflammation, Malnutrition, and Cardiac Disease as Predictors of Mortality in Hemodialysis Patients. J Am Soc Nephrol; 2002.

9. Margen R, Teruel JL,de la Cal A,Gámez C. the impact of malnutrition in morbidity and mortality in stable haemodialisis patients. Nephrol Dial Transplant; 1997.

10. Ordóñez Pérez V, Barranco Hernández E, Guerra Bustillo G, , Barreto Penié J, Santana Porbén S, Espinosa Borrás A,Martínez González C, Anías Martínez A. Estado nutricional de los pacientes con insuficiencia renal crónica atendidos en el programa de Hemodiálisis del Hospital Clínico-Quirúrgico "Hermanos Ameijeiras". nutricion hospitalaria; 2007.

11. Gariballa S, Forster S. Malnutrition is an independent predictor of 1-year mortality following acute illness. Salah Gariballa1,2* and Sarah Forster; 2007.

12. Barbora Knappe-Drzikova, Sebastian Maasberg,Dorothee Vonderbeck,Thomas A. Krafft,Sven Knüppel,Andreas Sturm,Jaqueline Müller-Nordhorn,Bertram Wiedenmann,Ulrich-Frank Pape. Malnutrition predicts long-term survival in hospitalized patients with gastroenterological and hepatological diseases. Clinical Nutrition ESPEN; 2019.

13. MUHLETHALER R, STUCK AE,MINDER CE, FREY BM. The Prognostic Significance of Protein-energy Malnutrition in Geriatric Patients. Age and Aging; 1995.

14. Sullivan DH, Walls RC,Lipschitz DA. protein-energy undernutrition and the risk of mortality within one year of hospital discharge in a select population of geriatric rehabilitation patients. Am J CLin Nutr; 1991.

15. Soler-Catalula JJ, Sanchez-Sanchez L,Martínez Garcia MA, Roman Sanchez P, Salcedo E,Navarro M. Mid-Arm Muscle Area Is a Better Predictor of Mortality Than Body Mass Index in COPD. CHEST; 2005.

16. SC, Ho, Wang JY, Kuo CD,Lee KY,Feng PH,Chen TT,Hsu MF. Mid-arm and calf circumferences are stronger mortality predictors than body mass index for patients with chronic obstructive pulmonary disease. Internacional Journal of COPD; 2016.

17. Jaqueline Rodrigues da Silva, Emanuelly Varea Maria Wiegert,Livia Oliveira,Larissa Calixto-Lima. Different methods for diagnosis of sarcopenia and its association with nutritional status and survival in patients with advanced cancer in palliative care. Nutrition; 2019.

18. Park S, ,Han B,,Won Cho J , Woo S, ,Kim S,Jin Kim S, Seog Kim W:. Effect of Nutritional Status on Survival Outcome of Diffuse Large B-Cell Lymphoma Patients Treated with Rituximab-CHOP. Nutrition and Cancer; 2015.

19. Ferrigno D, Buccheri G. Anthropometric measurements in non-small-cell lung cancer. Supportive Care in Cancer; 2001.

20. Anton Reepalu, Taye Tolera Balcha,,Sten Skogmar1,Nuray Güner,Erik Sturegård,Per Björkman. Factors Associated with Early Mortality in HIV-Positive Men and Women Investigated for Tuberculosis at Ethiopian Health Centers. Plus One; 2016.

21. Tiziana Montalcini, Marta Moraca,Yvelise Ferro,Stefano Romeo,Sebastiano Serra,Maria Girolama Raso,Francesco Rossi,Walter G. Sannita,Giuliano Dolce, Arturo Pujia. Nutritional parameters predicting pressure ulcers and short-term mortality in patients with minimal conscious state as a result of traumatic and non-traumatic acquired brain injury. J Transl Med; 2015.

22. Simpson F, Doig G. Early PN trial investigators Group. Phisical Assessment and Anthropometris Measures for Use in Clinical Research Conducted in Critically ill Patient Population: An analytic observational study. Journal of Parenteral and Enteral Nutrition ; 2013.

23. Ravasco P, Camilo ME,Gouveia-Oliveira A,Adam S, G., Brum. A critical approach to nutritional assessment in critically ill patients. Clinical Nutrition; 2002.

24. Supanum R, Atsawarungruangkit A. Nutritional Status and its Impact on Clinical Outcomes for Patients Admitted to Hospital with Cirrhosis. J Med Assoc Thai ; 2016.

25. Powell-Tuck J, Hennerssy EM. A comparison of mid upper arm circumference, body mass index and weight loss as indices of undernutrition in acutely hospitalized patients. Clinical NUtrition ; 2003.

26. Ordoñez AM, Madalozzo Schieferdecker ME, Cestonaro T, Cardoso Neto J,Ligocki Campos AC. Nutritional status influences the length of stay and clinical outcomes in hospitalized patients in internal medicine wards. Nutr Hosp; 2013.

27. Stephen B. Asiimwe, Conrad Muzoora,L. Anthony Wilson,Christopher C. Moore. Bedside measures of malnutrition and association with mortality in hospitalized adults. Clinical Nutrition; 2015.

28. Constans T, Bacq Y,Brecht,JF, Guilmot JL, COuchet P,Lamise F. Protein-energy malnutrition in elderly medical patients. J Am Geriatr Soc; 1992.

29. April Clugstona, Hugh M. Patersona,Kerry Yuillb,O. James Gardena,Rowan W. Parksa. Nutritional risk index predicts a high-risk population in patients with obstructive jaundice. clinical nutrition; 2006.

30. Sullivan DH, Bopp MM, Roberson PK. Protein-energy Undernutrition and Life-threatening Complications Among the Hospitalized Elderly. J GEN INTERN MED; 2002.

31. Jacob V, Marchant PR,Wild G,Brown CB,Moorhead PJ,El Nahas AM. Nutritional Profile of continuous ambulatory Peritoneal Dialysis Patients. Nephron ; 1995.

32. Valente da Silva, HG, Santos SO, Silva NO,Ribeiro FD,Josua LL,Moreira SB. Nutritional assessment associatedwithlengthof inpatients' hospital stay. Nutrición hospitalaria; 2012.

33. Almeida AI, Correia M,Camilo M,Ravasco P. Length of stay in surgical patients: nutritional predictive parameters revisited. British Journal of Nutrition; 2012.

34. Jeejeebhoy KN, Keller H,Gramlich L, Allard JP,Laporte M,Duerksen DR,Payette HP, Bernier P,Vesnaver E, Davidson B,Teterina A, Lou W. Nutritional assessment: comparison of clinical assessment and objective variables for the prediction of length of hospital stay and readmission. AJCN; 2015.

35. Tsaousi G, Panidis S,Stavrou G, Tsouskas J, Panagiotou D, Kotzampassi K. Prognostic Indices of Poor Nutritional Status and Their Impact on Prolonged Hospital Stay in a Greek University Hospital. BioMed Research International; 2014.

36. Real, Gustavo,Gonzales,¨hauf, Inara,Regina,Fru, Sedrez, Jose ́,,Henrique,Koth, Dall'Aqua, Eduarda,Jaine,Fachinello, Gonzalez, and,Maria,Cristina. Calf Circumference: A Marker of Muscle Mass as a Predictor of Hospital Readmission. Journal of Parenteral and Enteral Nutrition; 2018.

Topic 3: Biochemical analysis – Complication in surgical illness

Author(s):

Question: Complications during follow-up of surgical illnesses with biochemical alterations (albumin, prealbumin and C-reactive protein [CRP]).

Setting:

No. of studies	Certainty assessment						Impact	Certainty	Importance
	Study design	Risk of bias	Inconsistency	Indirectness	Imprecision	Other considerations			
Albumin and post-surgical complications in elderly patients with hip fracture surgery (follow-up: mean 3 years; evaluated with: Registry).									
1 [1]	observational studies	serious [a]	not serious	not serious	not serious		17,651 patients were included. Patients with hypoalbuminemia had higher rates of sepsis (RR 1.92; 95% CI 1.36–2.72, p<0.001), and higher rates of unscheduled intubation need (RR 1.51; 95% CI 1.21–1.88, p<0.001). Patients with hypoalbuminemia had longer hospital stays.	-	IMPORTANT
Albumin and early complications after hip fracture surgery (follow-up: mean 30 days; evaluated with: Registry).									
1 [2]	observational studies	serious [b]	not serious	not serious	not serious		12,373 patients were included. Hypoalbuminemia was an independent risk predictor for major complications (acute myocardial infarction, cardiac arrest, acute renal failure, sepsis, septic shock, stroke, pulmonary embolism, reintubation, prolonged intubation, and coma), with an OR for mild hypoalbuminemia of 1.21 (95% CI 1.01–1.46, p=0.042), moderate hypoalbuminemia OR 1.47 (95% CI 1.21–1.79, p<0.001) and severe hypoalbuminemia OR of 2.68 (95% CI 1.77–3.29, p<0.001). Hypoalbuminemia was also associated with a higher presence of minor complications (deep vein thrombosis, pneumonia, surgical wound complication and urinary tract infection), with OR of 1.05, 1.38 and 2.42 for mild, moderate and severe hypoalbuminemia, respectively.	-	IMPORTANT
Hypoalbuminemia and complications in patients with colon cancer surgery (follow-up: mean 30 days; evaluated with: Registry).									

No. of studies	Certainty assessment						Impact	Certainty	Importance
	Study design	Risk of bias	Inconsistency	Indirectness	Imprecision	Other considerations			
1 [3]	observational studies	serious [a]	not serious	not serious	not serious		5,143 patients included. Patients with preoperative hypoalbuminemia had more cardiac, pulmonary and septic events 30 days after surgery. Patients with hypoalbuminemia had longer duration of postoperative ileus and a longer hospital stay (especially patients undergoing open surgery), with OR between 1.43 and 1.94.	-	IMPORTANT
Albumin and postoperative complications after hip surgery (evaluated with: Meta-analysis).									
3 [4]	observational studies	serious [a]	not serious	not serious	not serious		10,942 patients. Hypoalbuminemia was significantly associated with an increased risk of postoperative complications, with a pooled OR of 1.89; 95% CI 1.06–3.38, p<0.001.	-	IMPORTANT
Perioperative albumin and suture failure in colorectal cancer (evaluated with: Registry).									
1 [5]	observational studies	serious [c]	not serious	not serious	not serious		200 patients who underwent curative laparoscopic surgery for colorectal cancer. Postoperative albumin in patients with anastomotic leak was lower than in patients without anastomotic leak. In multivariate analysis, day 1 and day 3 albumin were predictors of suture failure.	-	LIMITED IMPORTANCE
Preoperative albumin and complications after abdominal aortic aneurysm repair surgery (evaluated with: Registry).									
1 [6]	observational studies	serious [d]	not serious	not serious	not serious		15,002 patients. Both moderate and severe hypoalbuminemia were associated with longer hospital stay, higher pulmonary complications, and reoperation rates.	-	IMPORTANT
Hypoalbuminemia and complications after hip replacement surgery (follow-up: mean 30 days; evaluated with: Registry).									
1 [7]	observational studies	serious [a]	not serious	not serious	not serious		49,603 patients. Patients with hypoalbuminemia had a higher rate of complications, both in general and severe complications (sepsis, acute myocardial infarction, stroke, cardiac arrest, pulmonary embolism, coma, or death), higher rates of surgical site infection, higher rate of pneumonia, and longer hospital stay.	-	IMPORTANT

Explanations

a. Hypoalbuminemia was defined as albumin <3.5 g/dL.

b. Four albumin ranges were considered: normal: > 3.5 g/dL, mild hypoalbuminemia: 3.1–3.49 g/dL, moderate hypoalbuminemia: 2.4–3.1 g/dL, and severe hypoalbuminemia: albumin <2.4 g/dL.

c. Albumin on postoperative day 1 and 3 were considered.

d. Moderate hypoalbuminemia was defined as 2.8–3.5 g/dL; severe hypoalbuminemia was defined as <2.8 g/dL.

References

1. Bohl D, Shen M,Hannon C,Fillingham Y,Darrith B,Della Valle C. Serum albumin predicts survival and postoperative course following surgery for geriatric hip fracture. J Bone Joint Surg Am; 2017.

2. Chung A, Hustedt JW,Walker R,Jones C,Lowe J,Russell GV. Increasing severity of malnutrition is associated with poorer-30day outcomes in patients undergoing hip fracture surgery. J Orthop Trauma; 2018.

3. Haskins I, Baginsky M,Amdur RL,Agarwal S. Preoperative hypoalbuminemia is associated with worse outcomes in colon cancer patients. Clinical Nutrition; 2016.

4. Li S, Zhang J,Zheng H,Wang X,Liu Z,Sun T. Prognostic Role of Serum Albumin, Total Lymphocyte Count, and Mini Nutritional Assessment on Outcomes After Geriatric Hip Fracture Surgery: A Meta-Analysis and Systematic Review. J Arthroplasty; 2019.

5. Shimura T, Toiyama Y,Hiro J,Imaoka H,Fujikawa H,Kobayashi M et al. Monitoring preoperative serum albumin can identify anastomotic leakage in colorectal cancer patients with curative intent. Asian J Surg;

6. Inagaki E, Farber A,Eslami MH,Kalish J,Rybin DV,Doros G et al. Preoperative hypoalbuminemia is associated with poor clinical outcomes after open and endovascular abdominal aortic aneurysm repair. J Vasc Surg; 2016.

7. Bohl D, Shen M,Kayupov E,Della Valle CJ. Hypoalbuminemia independently predicts surgical site infection, pneumonia, length of stay and readmission after total joint arthroplasty. J Arthroplasty; 2016.

Topic 3: Biochemical analysis – Complication in medical illness

Author(s):

Question: Complications during follow-up of medical illnesses with biochemical alterations (albumin, prealbumin and CRP).

Setting:

No. of studies	Certainty assessment						Impact	Certainty	Importance
	Study design	Risk of bias	Inconsistency	Indirectness	Imprecision	Other considerations			
Prealbumin at admission and major cardiac events in patients with acute coronary syndrome (evaluated with: Registry).									
1 [1]	observational studies	serious [a]	not serious	not serious	not serious		610 consecutive patients admitted for acute coronary syndrome. Low prealbumin was an independent predictor of adverse cardiac events (death, acute heart failure, reinfarction, and cardiogenic shock).	-	IMPORTANT
Albumin and progression of chronic renal failure (follow-up: mean 2 years; evaluated with: Registry)									
1 [2]	observational studies	serious [b]	not serious	not serious	not serious		728 predialysis patients. The combination of low albumin. and low BMI was associated with an increased risk of chronic renal failure: OR 3.51 (95% CI 1.63–7.56). The largest differences were found between the low albumin + low BMI and high albumin + high BMI groups.	-	IMPORTANT
Albumin and severity of COVID-19 infection (evaluated with: Systematic literature review).									
11 [3]	observational studies	serious [c]	not serious	not serious	not serious		910 patients. Patients with hypoalbuminemia had an increased risk of severe COVID-19 infection (respiratory distress, need for ICU, and/or death): OR 12.6 (95% CI 7.5–21.1, $p<0.001$).	-	IMPORTANT
Serum CRP and infectious complications in bariatric surgery patients (evaluated with: Meta-analysis).									
6 [4]	observational studies	serious [d]	not serious	not serious	not serious		Elevated CRP levels can predict risk of postoperative infections in bariatric surgery. CRP on days 1, 3, and 5 has a high sensitivity and specificity and a negative prognostic value for postoperative infections.	-	IMPORTANT
Albumin and infections in the postoperative period of hip arthroplasty (evaluated with: Meta-analysis)									

No. of studies	Certainty assessment						Impact	Certainty	Importance
	Study design	Risk of bias	Inconsistency	Indirectness	Imprecision	Other considerations			
20 [5]	observational studies	serious [e]	not serious	not serious	not serious		18 studies demonstrated a correlation between preoperative hypoalbuminemia and worse postoperative outcome. In a meta-analysis of 8 studies, albumin <3.5 g/dL was independently associated with postoperative surgical wound infections (OR 2.176, 95% CI 1.9–2.4).	-	IMPORTANT
Albumin and surgical site infection in the postoperative period of orthopedic surgery (evaluated with: Meta-analysis).									
13 [6]	observational studies	serious [e]	not serious	not serious	not serious		112,183 patients included. Hypoalbuminemia (albumin <3.5 g/dL) was associated with a relative risk of infection of 2.39 (95% CI 1.57–3.64, p<0.0001). Surgical wound infection, deep tissue infection, and joint infection were considered.	-	IMPORTANT
Albumin and complications in elderly patients									
23 [7]	observational studies	serious [f]	not serious	not serious	not serious		In the community and in residential centers, albumin is positively associated with muscle strength measured by hand dynamometry and with autonomy for activities of daily living; albumin is negatively associated with the degree of disability. In hospitalized patients, higher albumin levels correlate with a shorter hospital stay. In patients with hip fracture, albumin <38 g/L is associated with a higher probability of post-surgical complications (cardiac, pulmonary, infections, hemorrhage, thromboembolic complications).	-	IMPORTANT

Explanations

a. The cut-off for prealbumin was <17 mg/dL.

b. Four albumin ranges combined with BMI were considered: albumin <4 g/dL + BMI <23.5; albumin > 4 g/dL + BMI <23.5; albumin <4 mg/dL + BMI > 23.5; albumin > 4 g/dL + BMI > 23.5. Progression of chronic renal failure was defined as a decrease in glomerular filtration rate of <30% or initiation of dialysis within 2 years.

c. Hypoalbuminemia was considered depending on the reference values of each laboratory.

d. The 6 studies used different times to measure postoperative CRP levels. 3 studies did them on day 1, 3 studies on day 3, and 3 studies on day 5.

e. Hypoalbuminemia was defined as albumin <3.5 g/dL.

f. In studies with hospitalized patients, mean albumin levels were 36.04 g/L (95% CI 34.81–37.28); in studies in the community mean albumin was 41.13 g/L (95% CI 40–26–42). Studies were very heterogenous.

References

1. Wang W, Wang C,Ren D,Li T,Yao H,Ma S. Low serum prealbumin levels on admission can independently predict in-hospital adverse cardiac events in patients with acute coronary syndrome. Medicine; 2018.

2. Kikuchi H, Kanda E,Mandai S,Akazawa M,Iimori S,Oi K,et al. Combination of low body mass index and serum albumin level is associated with chronic kidney disease progression: the chronic kidney disease-research of outcomes in treatment and epidemiology (CKD-ROUTE) study. Clin Exp Nephrol; 2016.

3. Aziz M, Fatima R,Lee-Smith W,Assaly R. The association of low serum albumin with severe COVID-19: a systematic review and meta-analysis. Critical Care; 2020.

4. Lee Y, McKechnie T,Doumouras A,Handler C,Eskicioglu C,Gmora S et al. Diagnostic Value of C-Reactive Protein Levels in Postoperative Infectious Complications After Bariatric Surgery: a Systematic Review and Meta-Analysis. Obes Surg; 2019.

5. gu A, Malahias M,Strigelli V,Nocon A,Sculco T,Sculco P. Preoperative Malnutrition Negatively Correlates With Postoperative Wound Complications and Infection After Total Joint Arthroplasty: A Systematic Review and Meta-Analysis. J Arthroplasty ; 2019.

6. Yuwen P, Chen W,Lv H,Feng C,Li Y,Zhang T,et al. Albumin and surgical site infection risk in orthopaedics: a meta-analysis. BMC Surgery; 2017.

7. Cabrerizo S, Cuadras D,Gomez-Busto F,Artaza-Artabe I,Marín-Ciancas F,Malafarina V. Serum albumin and health in older people: Review and meta analysis. Maturitas; 2015.

Topic 3: Biochemistry analysis – Mortality in medical illness

Author(s): Rosa Burgos

Question: Mortality during follow-up of medical illnesses with biochemical alterations (albumin, prealbumin and CRP).

Setting:

No. of studies	Certainty assessment						Effect			Certainty	Importance
	Study design	Risk of bias	Inconsistency	Indirectness	Imprecision	Other considerations	No. of events	No. of individuals	Rate (95% CI)		
Hypoalbuminemia and mortality during hospitalization and at discharge (follow-up: mean 1675 days; evaluated with: Registry).											
1 [1]	observational studies	serious [a]	not serious	not serious	not serious	strong dose-response gradient association	30,732 patients, 1875 died on admission and 12,201 during follow-up.<br>Albumin <2.5 mg/dL adjusted in-hospital mortality: OR 18.7 (95% CI 14.9–23.4); mortality during follow-up: OR 6.1 (95% CI 5.5–6.7). Albumin 2.5–3.5 mg/dL adjusted in-hospital mortality: OR 4.8 (95% CI 4.0–5.7); mortality during follow-up: OR 2.7 (95% CI 2.6–2.9)			⨁⨁⨁⨁ HIGH	CRITICAL
Albumin and mortality in hemodialysis patients (evaluated with: Meta-analysis of studies with mortality data).											
35 [2]	observational studies	serious [b]	not serious	not serious	serious [c]		Albumin was inversely associated with mortality (HR 0.7355, 95% CI 0.6775–0.7984), stronger in all-cause mortality than in cardiovascular mortality.<br>Prealbumin was not significantly associated with mortality in 4 studies.			-	IMPORTANT
Albumin and mortality in the elderly in the community (evaluated with: mortality registry).											
1 [3]	observational studies	serious [d]	not serious	not serious	not serious		77,531 patients > 65 years, mean follow-up 3.3 years. 3,840 persons died. Mortality risk gradually increased with decreasing albumin (all-cause mortality, cancer mortality, cardiovascular and respiratory death), HR for albumin <3.6 g/dL 2.84 (95% CI 2.4–3.27).			-	IMPORTANT
Hypoalbuminemia and 30-day mortality in patients hospitalized for acute medical illness (evaluated with: Registry).											
1 [4]	observational studies	serious [e]	not serious	not serious	not serious		5,894 patients included, 332 died within 30 days of admission.			-	IMPORTANT

No. of studies	Certainty assessment						Effect			Certainty	Importance
	Study design	Risk of bias	Inconsistency	Indirectness	Imprecision	Other considerations	No. of events	No. of individuals	Rate (95% CI)		
							Hypoalbuminemia (albumin <3.5 mg/dL) conferred a 30-day all-cause mortality OR of 1.95 (95% CI 1.31–2.9), adjusted for sex, age, and Charlson comorbidity index.				
Hypoalbuminemia and mortality in patients post-acute myocardial infarction (follow-up: mean 2240 days; evaluated with: Registry).											
1 [5]	observational studies	serious [f]	not serious	not serious	not serious		The risk of post-acute myocardial infarction mortality increased with decreasing albumin levels, with HR between 1.45 and 4.33 for 4 ranges of serum albumin.			-	IMPORTANT
Hypoalbuminemia and post-stroke mortality (follow-up: mean 3 months; evaluated with: Registry).											
1 [6]	observational studies	serious [g]	not serious	not serious	not serious		1,477 consecutive patients enrolled in 34 hospitals in the state of Georgia (USA). 10% (154 patients) died during hospitalization. Hypoalbuminemia (albumin <3.5 mg/dL) was an independent risk factor for mortality, with increasing mortality with decreasing album levels.			-	IMPORTANT
Hypoalbuminemia and long-term mortality in readmitted medical patients (follow-up: 3 years; evaluated with: Registry).											
1 [7]	observational studies	not serious	serious [h]	not serious	not serious		The long-term mortality rate in patients readmitted for medical illnesses is 4.6 times higher than in patients with normal albumin. Persistent hypoalbuminemia is associated with higher mortality.			-	IMPORTANT
Pretreatment hypoalbuminemia and mortality in cancer patients (evaluated with: Systematic literature review).											
59 [8]	observational studies	serious [i]	not serious	not serious	not serious		Gastrointestinal cancer: 26 of 29 studies found that higher albumin levels were associated with better survival in multivariate analysis. The same findings were reported by 9 of 10 studies in lung cancer, 6 of 6 studies in gynecologic or breast cancer, and 8 of 8 studies of cancer in other locations.			-	IMPORTANT
Hypoalbuminemia and mortality in peritoneal dialysis patients (follow-up: 4–80 months; evaluated with: Registry).											
1 [9]	observational studies	serious [j]	not serious	not serious	not serious		199 patients on peritoneal dialysis. Hypoalbuminemia, together with age > 65 years, presence of cardiovascular disease and diabetes			-	IMPORTANT

No. of studies	Certainty assessment						Effect			Certainty	Importance
	Study design	Risk of bias	Inconsistency	Indirectness	Imprecision	Other considerations	No. of events	No. of individuals	Rate (95% CI)		
							mellitus were independent predictors of mortality. Hypoalbuminemia: HR 2.3 (95% CI 1.1–5.0), p=0.03.				
Prealbumin and mortality in patients with systemic scleroderma (follow-up: median 48 months; evaluated with: Registry).											
1 [10]	observational studies	serious [k]	not serious	not serious	not serious		299 patients. Serum prealbumin was a predictor of mortality, independent of other disease-related risk factors (pulmonary, gastrointestinal or multiple organ involvement), with HR ranging from 2.58 (95% CI 1.21–5.49, p<0.001) to 4.73 (95% CI 2.3–9.74, p<0.001).			-	IMPORTANT
Prealbumin and mortality in patients with acute renal failure (follow-up: mean 90 days; evaluated with: Registry).											
1 [11]	observational studies	serious [l]	not serious	not serious	not serious		340 patients. Prealbumin <10 mg/dL at diagnosis of acute intestinal failure was associated with an increased risk of mortality (HR 2.55; 95% CI 1.18–5.49, p=0.02). Changes in prealbumin (decrease in more than 4 mg/dL of prealbumin) was associated with increased 90-day mortality (HR 1.79, 95% CI 1.06–3.03, p=0.03).			-	IMPORTANT
Albumin and complications in elderly patients. (evaluated with: Meta-analysis)											
23 [12]	observational studies	serious [m]	not serious	not serious	not serious		In patients in the community, there was a clear association between albumin levels and long-term mortality (between 3 and 12 years). In institutionalized patients, albumin was associated with short-term mortality (1 year) but not with long-term mortality. Considering hypoalbuminemia together with disability for activities of daily living and gait disturbances leads to mortality that can be 7.5 times higher in men and 12.5 times higher in women than that of patients with albumin >43 g/dL and without disability.			-	IMPORTANT

Explanations

a. Only patients with albumin determined at admission (24 hours) were included. Readmissions were not considered. Critical patients are not mentioned. There are more cancer patients in the severe hypoalbuminemia group.

b. Very heterogeneous sample size and follow-up time.

c. Includes 8 studies not adjusted for comorbidity.

d. Sample recruitment: volunteers who undergo a study of health in the elderly.

e. Only albumin at admission was considered, not albumin changes during hospitalization.

f. 2,982 patients with co-morbid illnesses and 610 patients without albumin evaluation were discarded.

g. Multicenter retrospective study including a multiracial sample (31% black, 65% white, 4% other).

h. Patients admitted to 5 medical units of a university hospital were included. Only admissions of more than one week were considered, and only if patients had more than one albumin evaluation during admission.

i. In some studies, albumin was analyzed as a categorical variable, with a cut-off of 3.5 g/dL being the most frequent; in other studies, it was used as a continuous variable.

j. Nutritional assessment and albumin determination was performed at a mean of 5 months after the start of peritoneal dialysis (range 1–12 months). Hypoalbuminemia was defined as albumin <3.8 g/dL.

k. The cut-off for prealbumin was <200 mg/L.

l. The cut-off for prealbumin was <10 mg/dL.

m. In the studies with hospitalized patients, mean albumin value was 36.04 g/L (95% CI 34.81–37.28); in studies in the community, mean albumin value was 41.13 g/L (95% CI 40.26–42). In both cases, there was high heterogeneity between studies.

References

1. Amit Akirov, Hiba Masri Iraqi,Alaa Atamna,Ilan Shimon. Low Albumin Levels are Associated with Mortality Risk in Hospitalized Patients. The American Journal of Medicine; 2017.

2. Herselman M, Esau N,Kruger JM,Labadarios D,Moosa MR. Relationship between serum protein and mortality in adults on long-term hemodialysis: Exhaustive review and meta-analysis. Nutrition; 2010.

3. Wu C, Hu H,Huang N,Chou Y,Li C,Chou Y. Albumin levels and cause-s`pecific mortality in community-dwelling older adults. Preventive Med; 2017.

4. Jellinge ME, Henriksen DP,Hallas P,Brabrand M. Hypoalbuminemia is a strong predictor of 30-Day all-cause mortality in acutelly admitted medical patients: a prospective, observational, cohort study. Plos One; 2014.

5. Plakht Y, Gilutz H,Shiyovich. Decreased admission serum albumin level is an independent predictor of long-term mortality in hospital survivors of acute myocardial infarction. Soroka Acute Myocardial Infarction II (SAMI-II) project. International Journal of Cardiology; 2016.

6. Famakin B, Weiss P,Hertzberg V,McClellan W,Presley R,Krompf K et al. Hypoalbuminemia predicts acute stroke mortality: Paul Coverdell Georgia Stroke Registry. J Stroke and Cerebrovasc Dis; 2010.

7. Touma E, Bisharat N. Trends in admission serum albumin and mortality in patients with hospital readmission. Int J Clin Pract; 2019.

8. Gupta D, Lis CG. Pretreatment serum albumin as a predictor of cancer survival: A systematic review of the epidemiological literature. Nutrition Jorunal; 2010.

9. Leinig CE, Moraes T,Ribeiro S,Riella MC,Olandoski M,Martins M et al. Predictive value of malnutrition markers for mortality in peritoneal dialysis patients. J Renal Nutrition; 2011.

10. Codullo V, Cereda E,Klersy C,Cavazzana I,Alpini C,Bonardi C et al. Serum prealbumin is an independent predictor of mortality in systemic sclerosis outpatients. Rheumatology; 2016.

11. Wang W, Tang X,Hao G,Xie Y,Ma S,Luo J,et al. Serum prealbumin and its chganges over time are associated with mortality in acute kidney injury. Nature. Scientific Reports; 2016.

12. Cabrerizo S, Cuadras D,Gomez-Busto F,Artaza-Artabe I,Marín-Ciancas F,Malafarina V. Serum albumin and health in older people: Review and meta analysis. Maturitas; 2015.

Topic 3: Biochemistry analysis – Mortality in surgical illness

Author(s): Rosa Burgos

Question: Mortality during follow-up of surgical illnesses with biochemical alterations (albumin, prealbumin and CRP).

Setting:

No. of studies	Certainty assessment						Impact	Certainty	Importance
	Study design	Risk of bias	Inconsistency	Indirectness	Imprecision	Other considerations			
Preoperative albumin and mortality in elderly patients (>65 years) (evaluated with: Systematic review of the literature).									
7 [1]	observational studies	serious [a]	not serious	not serious	not serious		In 5 studies, preoperative albumin was predictive of mortality during admission, at 6, and at 12 months, with odds ratios between 6.82 and 2.32. In 2 studies, no significant association was found with in-hospital mortality or overall mortality.	-	IMPORTANT
Pretreatment albumin/globulin and mortality in cancer patients (evaluated with: Meta-analysis).									
17 [2]	observational studies	serious [b]	not serious	not serious	not serious		11,123 patients evaluated preoperatively for digestive, respiratory, urinary and other neoplasms. Low preoperative albumin/globulin ratio was associated with lower survival after cancer surgery (HR 1.85, 95% CI 1.57–2.21).	-	IMPORTANT
Pretreatment albumin and survival in patients with head and neck cancer (evaluated with: Registry).									
1 [3]	observational studies	serious [c]	not serious		not serious		216 patients. Pretreatment albumin levels <3.5 g/dL were predictive of greater disease progression and worse survival, with a higher mortality hazard ratio than other strong prognostic factors such as TNM stage and tumor location.	-	IMPORTANT
Pre-surgery prealbumin and mortality after hepatectomy (evaluated with: Registry)									
1 [4]	observational studies	serious [d]	not serious	not serious	not serious		526 patients who underwent curative hepatectomy for hepatocarcinoma. Preoperative prealbumin was an independent prognostic factor for overall survival, adjusting for age, sex, tumor size, number of tumors, completeness of tumor capsule, cirrhosis, hepatitis B surface antigen, Child-Pugh stage,	-	IMPORTANT

No. of studies	Certainty assessment						Impact	Certainty	Importance
	Study design	Risk of bias	Inconsistency	Indirectness	Imprecision	Other considerations			
							and Barcelona Clinic Liver Cancer stage. HR for prealbumin <200 mg/L 1.603 (95% CI 1.246–2.063), HR for prealbumin <182 mg/L 1.683 (95% CI 1.268–2.115); both p<0.001.		
Postoperative albumin and mortality in gastric cancer (follow-up: median 10 years; evaluated with: Registry).									
1 [5]	observational studies	serious [e]	not serious	not serious	not serious		135 patients >75 years who underwent surgery for gastric cancer. Albumin levels one month after surgery was an independent prognostic factor for overall survival, together with lymphatic invasion, distant metastases, histology, and American Society of Anesthesiologists score.	-	LIMITED IMPORTANCE
Preoperative albumin and mortality after abdominal aortic aneurysm surgery (follow-up: median 7 years; assessed with: registry).									
1 [6]	observational studies	serious [f]	not serious	not serious	not serious		15,002 patients undergoing abdominal aortic aneurysm repair surgery (4,956 open abdominal surgery and 10,046 endovascular surgery). 30-day mortality was higher in patients undergoing abdominal surgery with moderate hypoalbuminemia (2.8–3.5 g/dL) (OR 1.32; 95% CI 1.02–1.7; p=0.035) and with severe hypoalbuminemia (OR 1.92; 95% CI 1.37–2.7; p<0.01).<br><br>In endovascular surgery, moderate and severe hypoalbuminemia were also independent predictors of 30-day mortality (OR 1.9; 95% CI 1.38–2.62 for moderate hypoalbuminemia; OR 2.98, 95% CI 1.96–4.53 for severe hypoalbuminemia; p<0.001 for both	-	IMPORTANT
Preoperative albumin and mortality after colon cancer surgery (follow-up: median 2 years; assessed with: registry).									
1 [7]	observational studies	not serious	not serious	not serious	not serious		5,143 patients with a primary indication for surgery for colon cancer. Mortality 30 days after surgery was higher in patients with hypoalbuminemia in a multivariate model controlled for age, sex, BMI, functional status, smoking, hypertension, heart failure, COPD, diabetes mellitus, and weight loss of ≥ 10% prior to surgery. This association was maintained the type of surgery: open (OR 1.76; 95% CI 1.37–2.26; p<0.0001) and laparoscopic (OR 2.31; 95% CI 1.81–	-	CRITICAL

No. of studies	Certainty assessment						Impact	Certainty	Importance
	Study design	Risk of bias	Inconsistency	Indirectness	Imprecision	Other considerations			
							2.94; p<0.0001). The optimal albumin cut-off for greater sensitivity, specificity and positive predictive value was 3.1 g/dL.		
Preoperative albumin and mortality in hip fracture (follow-up: median 1 year; evaluated with: Registry).									
3 [8,9,10]	observational studies	serious [g]	not serious	not serious	not serious		549, 12,373 patients and 17,651 patients, respectively, >65 years of age, who underwent surgery for hip fracture. Hypoalbuminemia (albumin <3.5g/dL), and especially albumin <2.5–2.8 g/dL was associated with higher mortality at 1 month in all 3 studies and at 4 months and 1 year in another study, with HR ranging from 1.31 to 3.46, p<0.01.	-	
Preoperative albumin and mortality in lung transplantation (follow-up: mean 1 year; evaluated with: Registry).									
1 [11]	observational studies	not serious	not serious	not serious	not serious		453 patients who underwent lung transplantation. Preoperative hypoalbuminemia, especially albumin <3 g/dL, was an independent risk factor for mortality 1 year after transplantation.	-	

Explanations

a. Differences in criteria for defining hypoalbuminemia; albumin <2.8 g/dL in 1 study, <3.0 g/dL in 3 studies, and <3.5 g/dL in 2 studies.

b. All studies were conducted in China or Japan, only one study was conducted in the USA. Follow-up periods were highly variable.

c. Patients underwent surgery and/or radiation therapy/chemoradiotherapy.

d. Large number of patients excluded due to lack of baseline data. Prealbumin was determined on the second day of hospitalization, without specifying whether it was post-surgery. Mean follow-up was 56 months, a high number of patients lost to follow-up.

e. Only patients > 75 years of age were included.

f. Data for patients who required emergency surgical intervention were not separated from those of scheduled surgeries.

g. Different definitions of hypoalbuminemia: ranges of 28–33 g/L and <28 g/L in one study, and ranges of 3.1–3.5, 2.4–3.1 and <2.5 g/dL in another.

References

1. Van Stijn M, Korkic-Halilovic I,Bakker M,Van der Ploeg T,Van Leeuwen M,Houdijk A. Preoperative nutrition status and postoperative outcome in elderly general surgery patients: a systematic review. JPEN; 2012.

2. Lv G, An L,Sun X,Hu Y,Sun D. Pretreatment albumin to globulin ratio can serve as a prognostic maker in human cancers: a meta-analysis. Clinical Chimica Acta; 2018.

3. Lim W, Roh J,Kim S,Choi S,Nam S,Kim S. Pretreatment albumin level predicts survival in head and neck squamous cell carcinoma. Laryngoscope; 2017.

4. Jia R, Zhong J,Huo R,Su Q,Xiang X,Zhao F,et al. Correlation between serum prealbumin and prognosis of patients with hepatocellular carcinoma after hepatectomy. J Surg Oncol ; 2019.

5. Saito H, Kono Y,Murakami Y,Shishido Y,Kuroda H,Matsunaga T et al. Postoperative serum albumin is a potential prognostic factor for older patients with gastric cancer. Yonago Acta Medica ; 2018.

6. Inagaki E, Farber A,Eslami MH,Kalish J,Rybin DV,Doros G et al. Preoperative hypoalbuminemia is associated with poor clinical outcomes after open and endovascular abdominal aortic aneurysm repair. J Vasc Surg; 2016.

7. Haskins I, Baginsky M,Amdur RL,Agarwal S. Preoperative hypoalbuminemia is associated with worse outcomes in colon cancer patients. Clinical Nutrition; 2016.

8. Helminen H, Luukkaala T,Saarnio J,Nuotio M. Comparison of the Mini-Nutritional Assessment short and long form and serum albumin as prognostic indicators of hip fracture outcomes. Injury; 2017.

9. Chung A, Hustedt JW,Walker R,Jones C,Lowe J,Russell GV. Increasing severity of malnutrition is associated with poorer-30day outcomes in patients undergoing hip fracture surgery. J Orthop Trauma; 2018.

10. Bohl D, Shen M,Hannon C,Fillingham Y,Darrith B,Della Valle C. Serum albumin predicts survival and postoperative course following surgery for geriatric hip fracture. J Bone Joint Surg Am; 2017.

11. Chamogeorgakis T, Mason D,Murthy S,Thuita L,Raymond D,Petterson G et al. Impact of nutritional state on lung transplant outcomes. J Heart Lung Transplant; 2013.

Topic 4: Hand grip strength

Author(s): Gabriel Olveira

Question: Prognostic value of altered dynamometry in patients with disease-related malnutrition (or at risk of malnutrition) during short- and medium-term follow-up.

Setting: General population is not included. Chronic or acute illnesses are included.

No. of studies	Certainty assessment						Effect			Certainty	Importance
	Study design	Risk of bias	Inconsistency	Indirectness	Imprecision	Other considerations	No. of events	No. of individuals	Rate (95% CI)		
All-cause mortality in patients (inpatient and outpatient clinical populations at risk for malnutrition) (assessed with: low vs high muscle strength)											
20 [1,a]	observational studies	serious [b]	serious [c]	serious [a,d]	serious [e]	strong association. All possible residual confounding factors could reduce the observed effect	4478	35,657	Event rate: 1.8 (1.54 to 2.1)	⊕⊕◯◯ LOW	CRITICAL [bc]
All-cause mortality in patients (clinical populations with diseases at risk of malnutrition or malnourished) (evaluated with: 5 kg increase in strength).											
20 [1]	observational studies	very serious [d,f,g]	very serious [h]	serious [d]	serious [e]	all possible residual confounding factors could reduce the observed effect			Event rate: 0.72 (0.59 to 0.89)	⊕◯◯◯ VERY LOW	CRITICAL [bd]
All-cause mortality in patients with chronic kidney disease (follow-up: 13–84 months; assessed with: low vs. high strength).											
9 [1]	observational studies	serious [a,d]	serious [i]	serious [d]	not serious [e]	all possible residual confounding factors could reduce the observed effect	609	2921	Event rate: 1.84 (1.37 to 2.47)	⊕⊕◯◯ LOW	CRITICAL [bc]
All-cause mortality in dialysis patients. Normal vs. low cut-off values (assessed with: Clinical mortality registry).											
6 [2,g]	observational studies	serious [f]	not serious	serious [d]	not serious	all possible residual confounding factors could reduce the	456	1996	Event rate: 1.88 (1.51	⊕⊕⊕◯ MODERATE	CRITICAL

No. of studies	Certainty assessment						Effect			Certainty	Importance
	Study design	Risk of bias	Inconsistency	Indirectness	Imprecision	Other considerations	No. of events	No. of individuals	Rate (95% CI)		
						observed effect			to 2.33)		
All-cause mortality in dialysis patients (1 kg increase in dynamometry and its association with mortality) (follow-up: 1–3.5 years)											
3 [2,f,g]	observational studies	serious [f]	not serious	serious [d]	not serious	all possible residual confounding factors could reduce the observed effect	133	779	Event rate: 1.89 (1.42 to 2.52)	⊕⊕⊕◯ MODERATE	CRITICAL
All-cause mortality in cancer patients (follow-up: 3–29 months; evaluated with: low vs high muscle strength).											
6 [1]	observational studies	serious [j]	not serious	serious [d]	serious [k]	all possible residual confounding factors could reduce the observed effect	501	1370	Event rate: 2.4 (1.57 to 3.59)	⊕⊕◯◯ LOW	CRITICAL
Mortality in cancer patients (gastrointestinal and breast) with adjuvant treatment (evaluated with: Clinical registry).[l]											
1 [3]	observational studies	serious [b]	serious [m]	serious [d]	serious [n]	all possible residual confounding factors could reduce the observed effect	85	228	Event rate: 1.7 (0.897 to 3.324)	⊕◯◯◯ VERY LOW	CRITICAL
Mortality in patients with hip fracture with one-year follow-up (evaluated with: Medical history).											
1 [4,o]	observational studies	not serious	not serious	serious [d]	serious [p]	none	118	509	Event rate: 2.088 (1.72 to 3.718)	⊕⊕◯◯ LOW	CRITICAL
Mortality after admission for hip fracture (evaluated with: medical history, telephone calls).											
1 [5,q]	observational studies	very serious [r]	not serious	serious [d]	very serious [r,s]	all possible residual confounding factors could reduce the	Dynamometry was not significant in terms of mortality (0.97–1.04) or risk of falls at three months (0.95–1.01). [q]			⊕◯◯◯ VERY LOW	CRITICAL

No. of studies	Certainty assessment						Effect			Certainty	Importance
	Study design	Risk of bias	Inconsistency	Indirectness	Imprecision	Other considerations	No. of events	No. of individuals	Rate (95% CI)		
						observed effect					
Mortality and/or readmission in elderly patients after hospitalization for pneumonia.											
2 [6,7,8]	observational studies	serious [t]	serious [u]	serious [d]	serious [v]	all possible residual confounding factors could reduce the observed effect	366 total elderly patients admitted for pneumonia in two studies. Dynamometry was assessed as a predictor of discharge or mortality and/or readmissions. In Vecchiarino et al, it was more strongly associated with discharge (4.6 (2.102–10.375) and death (0.370 (0.149–0.992). In Bohannon's study, it was associated with lower mortality and/or readmissions: HR 0.969 (0.948–0.987).			⊕◯◯◯ VERY LOW	CRITICAL
All-cause mortality in patients with COPD (follow-up: 24–73 months; evaluated with: low vs high strength).											
3 [1]	observational studies	serious [a]	not serious [i]	serious [d]	serious [e]	all possible residual confounding factors could reduce the observed effect	283	1640	Event rate: 1.36 (1.16 to 1.6)	⊕⊕◯◯ LOW	CRITICAL [bc]
All-cause mortality in critically ill patients (evaluated with: high vs. low strength).											
6 [1]	observational studies	serious [d,j]	very serious [w]	serious [d,f,g]	very serious [e]	all possible residual confounding factors could reduce the observed effect	313	1365	Event rate: 2.06 (1.33 to 3.21)	⊕◯◯◯ VERY LOW	CRITICAL [bc]
All-cause mortality in metabolic (diabetes, hypertension) and cardiovascular patients (follow-up: 12–219 months; evaluated with: low vs. high strength).											
6 [1]	observational studies	serious [a,d,f,g]	very serious [x]	serious [d,f]	serious [e]	strong association. All possible residual confounding factors could reduce the	2772	28361	Event rate: 1.64 (1.26 to 2.14)	⊕◯◯◯ VERY LOW	CRITICAL [bc]

No. of studies	Certainty assessment						Effect			Certainty	Importance
	Study design	Risk of bias	Inconsistency	Indirectness	Imprecision	Other considerations	No. of events	No. of individuals	Rate (95% CI)		
						observed effect					
Modifications of cancer treatment during neoadjuvant chemoradiotherapy in esophageal cancer (aggregate event: discontinuation, reduction, suspension of treatment, hospitalization, death) (evaluated with: Clinical registry).[y]											
1 [9,z]	observational studies	serious [aa]	not serious	serious [d]	serious [ab]	all possible residual confounding factors could reduce the observed effect	29	162	Event rate: 0.939 (0.882 to 1)	⨁⨁◯◯ LOW	IMPORTANT
Morbidity in surgical patients (multiple specialties and illnesses) (evaluated with: Medical history)											
14 [7,10,ac]	observational studies	serious [ad]	not serious [ad]	serious [d]	serious [ae]	all possible residual confounding factors could reduce the observed effect	Of the 17 articles included in the systematic review by Sultan et al, we selected 14 that measured complications in 1,543 patients. The heterogeneity in study design and the diversity of surgical procedures employed precluded a formal meta-analysis. Despite the moderate quality of these observational studies, hand dynamometry was associated with increased morbidity in the form of complications (10 studies), mortality (2/5 studies) and length of hospital stay (3/7 studies). [10,ad]			⨁⨁◯◯ LOW	IMPORTANT
Probability of admitted cancer patients (medical or surgical) of being discharged at 30 days (and mean length of stay) (evaluated with: Medical history).											
1 [11,af]	observational studies	very serious [ag]	not serious	serious [d]	serious [ah]	all possible residual confounding factors could reduce the observed effect		130	Event rate: 0.33 (0.19 to 0.55)	⨁◯◯◯ VERY LOW	IMPORTANT
Mean length of stay in geriatric inpatients (evaluated with: Medical history).											
1 [12,ai]	observational studies	serious [aj]	not serious	not serious [d]	very serious [ak]	all possible residual confounding factors could reduce the	634 patients. Patients with dynapenia (regardless of whether they were obese, malnourished or had normal weight) were hospitalized			⨁⨁◯◯ LOW	IMPORTANT

No. of studies	Certainty assessment						Effect			Certainty	Importance
	Study design	Risk of bias	Inconsistency	Indirectness	Imprecision	Other considerations	No. of events	No. of individuals	Rate (95% CI)		
						observed effect	approximately 4 more days than patients with normal weight/no dynapenia (from 4.7 to 4.05 days).				
Admission to ICU after hip fracture (complications) (evaluated with: Medical history).											
1 [13,al]	observational studies	very serious [am]	not serious	serious [an,d]	serious [s]	all possible residual confounding factors could reduce the observed effect	83 patients. Multivariate analysis revealed that dynamometric strength (Jamar dynamometer) was associated with decreased odds of ICU admission, assessed as a continuous variable: OR 0.95 (0.92–0.98) p 0.001.			⊕◯◯◯ VERY LOW	IMPORTANT
Hospitalization in patients with type 2 diabetes mellitus (per 1 kg of dynamometry) (follow-up: mean 2.36 years; evaluated with: Medical history).											
1 [14,ao,ap]	observational studies	serious [ap]	not serious	serious [d]	serious [aq]	all possible residual confounding factors could reduce the observed effect	556	1282	Event rate: 0.964 (0.951 to 0.977)	⊕⊕◯◯ LOW	IMPORTANT
Cardiovascular events in patients with type 2 diabetes (per 1 kg of dynamometry) (follow-up: mean 2.36 years; evaluated with: Medical history).											
1 [14,ao,ap,aq]	observational studies	serious [ar]	not serious	not serious [d]	serious [as]	all possible residual confounding factors could reduce the observed effect	14	1282	Event rate: 0.899 (0.819 to 0.9871)	⊕⊕⊕◯ MODERATE	IMPORTANT
Exacerbations in COPD patients (increase of 1 kg dynamometry) (follow-up: mean 2.6 years; evaluated with: medical history).											
1 [15,at]	observational studies	serious [au]	not serious	serious [d]	serious [av]	all possible residual confounding factors could reduce the observed effect	92	272	Event rate: 1.04 (1.01 to 1.071)	⊕⊕◯◯ LOW	IMPORTANT
Risk of hospital discharge in hospitalized elderly patients (mean age: 84 years).											

No. of studies	Certainty assessment						Effect			Certainty	Importance
	Study design	Risk of bias	Inconsistency	Indirectness	Imprecision	Other considerations	No. of events	No. of individuals	Rate (95% CI)		
1 [16]	observational studies	serious [aw]	serious [ax]	serious [d]	serious [ay]	all possible residual confounding factors could reduce the observed effect	1 kg increase in dynamometry was associated with a 3% increase in likelihood of discharge: HR 1.03 (CI 1.001–1.007).			⨁◯◯◯ VERY LOW	IMPORTANT
Readmission risk in patients with multiple myeloma after bone marrow transplantation.											
1 [17]	observational studies	serious [az]	not serious	serious [d]	serious [ba]	all possible residual confounding factors could reduce the observed effect	13	100	Event rate: 0.9 (0.82 to 0.98)	⨁⨁◯◯ LOW	IMPORTANT
Risk of serious complications in surgical cancer patients.											
1 [18]	observational studies	serious [bb]	serious [ax,bb]	serious [d]	serious [ax,bb]	all possible residual confounding factors could reduce the observed effect	19	60		⨁◯◯◯ VERY LOW	IMPORTANT

COPD, chronic obstructive pulmonary disease.

Explanations

a. Although the meta-analysis was mostly based on hand dynamometry studies, some studies included dynamometry with the lower limbs and other measures. Therefore, the calculated risk could include measures of strength other than with a hand dynamometer.

b. Heterogeneous sample; after adjusting, significance is lost.

c. High heterogeneity between populations that cannot be explained by subgroup analysis and meta-regression of possible confounders. I=88.85% p<0.001.

d. Indirect evidence in patients who were possibly malnourished. Applicability may not be the same in other groups of patients studied in the guideline. Populations may differ in varying degrees of disease severity.

e. Wide confidence intervals in some patient groups.

f. Different dynamometers and different cut-off values are used, not all at the same time. Cut-off points are not comparable to each other.

g. All studies compare low dynamometry with normal dynamometry with different dynamometers and different cut-off values (this information can only be found by reviewing the literature).

h. Very heterogeneous sample I=97%.

i. Moderately heterogeneous sample I=64%.

j. “Critical patients” is a heterogeneous sample.

k. Moderately heterogeneous sample I=42%.

l. Kaplan-Meier curves were significant with a difference of 22 vs 34 months in patients with low dynamometry (Jamar dynamometer); however, when performing Cox regression (multivariate) analysis, significance is lost. There may be selection biases and the sample is heterogeneous.

m. Heterogeneous sample; after adjustment, significance is lost.

n. Wide confidence interval, which reaches the value of 1.

o. Article with one-year follow-up of 509 patients. Mean age 85.6 years. Controlled for multiple variables: age, functional status, BMI, cognitive impairment, cardiac impairment, anemia, and vitamin D. Cut-off points: 23 kg H; 13 kg M. Jamar.

p. This study could be regarded as precise too. The confidence interval is not too wide 1.7 to 3.7.

q. Low quality study. 378 patients, mean age 79.6 years. Assessed mortality and risk of falls at discharge. Unlike muscle mass, dynamometry is not associated with mortality or risk of falls in univariate or multivariate analyses. Considerable bias due to very short follow-up.

r. Three-month follow-up only.

s. Narrow confidence interval.

t. Only 153 patients admitted for pneumonia in one center are evaluated. There may be bias in the selection for comparability purposes.

u. Only one study; inconsistency cannot be adequately evaluated.

v. Wide confidence intervals. Disparate criteria.

w. Very heterogeneous sample I=75%.

x. Very heterogeneous sample I=88%.

y. Dynamometry is measured as over or under the 10th percentile. No selection bias in comparability or in the recording of events are found.

z. In this case, it would be protective, given the comparison of malnourished vs normal.

aa. Relatively small sample size and the confidence interval reaches the value of 1. p0.05. Analysis is adjusted for age and sex but not for tumor stage.

ab. Sample size is not too large.

ac. Data are taken from Sultan et al. However, the article by Norman et al is included, since it evaluates 4 articles for evaluation of surgical patients, 3 of which are included in the article by Sultan et al.

ad. Systematic review evaluating postoperative complications in surgical patients. The heterogeneity in the studies precludes a meta-analysis. The classification of the studies according to the Newcastle-Ottawa tool is low for most (<7). Dynamometry deficit is generally defined as <85% of control or population values. Mortality, length of stay, and complications of different types are evaluated. All articles were published before 2010, most in the 80s and 90s.

ae. Great variability in results, difficulty harmonization. Different number of patients. Some studies are case controls, others prospective cohorts.

af. A mechanical dynamometer (Smedley Hand) was used in the first 48 hours of hospitalization. Results were presented by tertiles. The highest handgrip strength tertile by sex was used as reference.

ag. Heterogeneity of patient's characteristics. Medical and surgical patients, solid and hematologic cancers. Smalls sample size of 130 patients. Only adjusted for age and education.

ah. Imprecise due to it being a single study with a small sample size.

ai. Multicenter study with 634 patients, mean age 80.9 years. Jamar dynamometer with different cut-off values based on BMI. Controlled for confounding variables but do not show confidence intervals.

aj. Sample is heterogeneous. The length of stay is measured and may be highly dependent on the how care was organized.

ak. Confidence intervals are not included.

al. Low-quality study with only 83 patients. The complication considered is admission to the ICU. Multivariate regression analysis is conducted with Jamar as a continuous variable. The study concludes that red blood cell distribution together with dynamometry predict well complications.

am. ICU admission evaluated; only 93 patients, single-center study.

an. A surrogate is used for evaluating complications.

ao. Follow-up study in Japanese 1282 patients with diabetes, mean age 64 years, follow-up for 2.36 years. 556 patients were admitted, 20 died, and 14 had cardiovascular events. Smedley dynamometer cut-off values: 26 men and 18 women. Significant findings for hospitalization. Regression analysis is conducted for each 1-kg increase in dynamometry and not by cut-off values. Each increase has a protective effect.

ap. Japanese patients. Hospitalization events could be biased although they are frequent events. Few events of cardiovascular disease.

aq. Confidence interval is different for men and women.

ar. Only 14 patients had cardiovascular events.

as. Confidence interval almost reaches 1.

at. 272 patients with COPD followed for 2.6 years. Analysis is controlled for different variables. The risk of total and severe exacerbations is evaluated, both significant. Jamar: was low in 64% of patients. Multivariate regression analysis is conducted based on 1-kg increases in dynamometry.

au. 272 patients with COPD is a reasonable sample size but not too large. A strength of the study is its multicenter design.

av. Although the increase in risk per kg of dynamometry is measured, the confidence intervals almost reach 1.

aw. Small sample of only 120 patients. Possibility of selection bias. Single-center study.

ax. Single study.

ay. Narrow confidence interval.

az. Few events (13 out of 100) and also the event is not direct complication but readmission.

ba. Confidence interval close to the value of 1. Small sample size but multivariate study is adjusted for many confounding factors.

bb. Small sample size. Performed without adjusting for confounding factors.

References

1. Jochem C1, Leitzmann M1,Volaklis K2,Aune D3,Strasser B4. Association Between Muscular Strength and Mortality in Clinical Populations: A Systematic Review and Meta-Analysis. J Am Med Dir Assoc. 2019; 2019.

2. Hwang S-H, Lee DH Min J Jeon JY. Handgrip Strength as a Predictor of All-Cause Mortality in Patients With Chronic Kidney Disease Undergoing Dialysis: A Meta-Analysis of Prospective Cohort Studies. J Ren Nutr. 2019 Mar 28;; 2019.

3. Mauricio SF, Ribeiro HS Correia MITD. Nutritional Status Parameters as Risk Factors for Mortality in Cancer Patients. Nutr Cancer. 2016;68(6):949–57; 2016.

4. Menéndez-Colino R, Alarcon T,Gotor P,Queipo R,Ramírez-Martín R,Otero A,et al. Baseline and pre-operative 1-year mortality risk factors in a cohort of 509 hip fracture patients consecutively admitted to a co-managed orthogeriatric unit (FONDA Cohort). Injury. 2018(3):656–61. ; 2018.

5. Reijnierse EM, Verlaan S,Pham VK,Lim WK,Meskers CGM,Maier AB. Lower skeletal muscle mass at admission independently predicts falls and mortality three months post-discharge in hospitalised older patients. J Gerontol A Biol Sci Med Sci 2018. 2018; ; 2018.

6. Bohannon RW, Maljanian R,Ferullo J. Mortality and readmission of the elderly one year after hospitalization for pneumonia. Aging Clin Exp Res. 2004 Feb;16(1):22–5. ; 2004.

7. Norman K, Stobäus N Gonzalez MC Schulzke JD Pirlich M. Hand grip strength: Outcome predictor and marker of nutritional status. Clin Nutr. 2011;30(2):135–42; 2011.

8. Vecchiarino P, Bohannon RW,Ferullo J,Maljanian R. Short-term outcomes and their predictors for patients hospitalized with community-acquired pneumonia. Heart Lung. 2004. 33(5):301–7. ; 2004.

9. Lakenman P, Ottens-Oussoren K Witvliet-Van Nierop J Van Der Peet D De Van Der Schueren M. Handgrip Strength Is Associated with Treatment Modifications during Neoadjuvant Chemoradiation in Patients with Esophageal Cancer. Nutr Clin Pract. 2017;32(5):652–7.; 2017.

10. Sultan P, Hamilton MA,Ackland GL. Preoperative muscle weakness as defined by handgrip strength and postoperative outcomes: A systematic review. BMC Anesthesiol. 2012;12(1):1. ; 2012.

11. Mendes J, Alves P,Amaral TF. Comparison of nutritional status assessment parameters in predicting length of hospital stay in cancer patients. Clin Nutr. 2014;33(3):466–70. ; 2014.

12. Rossi AP, Fantin F,Abete P,Bellelli G,Bo M,Cherubini A,et al. Association between hospitalization-related outcomes, dynapenia and body mass index: The Glisten Study. Eur J Clin Nutr. 2018;1–8. ; 2018.

13. Ji HM, Han J,Bae HW,Won YY. Combination of measures of handgrip strength and red cell distribution width can predict in-hospital complications better than the ASA grade after hip fracture surgery in the elderly. BMC Musculoskelet Disord. ; 2017.

14. Hamasaki H, Kawashima Y,Katsuyama H,Sako A,Goto A,Yanai H. Association of handgrip strength with hospitalization, cardiovascular events, and mortality in Japanese patients with type 2 diabetes. Sci Rep. 2017;7(1):1–9. ; 2017.

15. Martinez CH, Diaz AA,Meldrum CA,McDonald MLN,Murray S,Kinney GL,et al. Handgrip strength in chronic obstructive pulmonary disease associations with acute exacerbations and body composition. Ann Am Thorac Soc. 2017;14(11):1638–45; 2017.

16. Sayer, A.,Kerr,,H.,E.,Syddall,,C.,Cooper,,G.,F.,Turner,,R.,S.,Briggs,,A.,Aihie. Does admission grip strength predict length of stay in hospitalised older patients ?. Age and Ageing, Volume 35, Issue 1, January 2006, Pages 82–84, 2016;(December):82–4.; 2006.

17. Rosko AE, Huang Y,Benson DM,Efebera YA,Hofmeister C,Jaglowski S,et al. Use of a comprehensive frailty assessment to predict morbidity in patients with multiple myeloma undergoing transplant. J Geriatr Oncol. 2018;; 2018.

18. Härter J, Orlandi SP,Gonzalez MC. Nutritional and functional factors as prognostic of surgical cancer patients. Support Care Cancer. 2017;25(8):2525–30. ; 2017.

Topic 5: Phase angle

Author(s): Virginia Bellido

Question: Changes in phase angle during follow-up in patients with disease-related malnutrition (or at risk of malnutrition).

Setting:

No. of studies	Certainty assessment						Effect			Certainty	Importance
	Study design	Risk of bias	Inconsistency	Indirectness	Imprecision	Other considerations	No. of events	No. of individuals	Rate (95% CI)		
All-cause mortality in clinical populations with malnutrition or at risk of malnutrition.											
48 [1]	observational studies	serious [a,b]	serious [c]	serious [d]	serious [e]	all possible residual confounding factors could reduce the observed effect	2100	7651		⊕◯◯◯ VERY LOW	IMPORTANT
All-cause mortality in cancer patients (follow-up: 4–70 months; evaluated with: phase angle cut-off <4.4° to <5.8°).											
16 [1,2,3,4,5,6,7,8,9,10,11,12,13,14,15,16,17]	observational studies	serious [a,b,f,g,h,i]	serious [j]	serious [d]	serious [e,k,l]	all possible residual confounding factors could reduce the observed effect	649	1386		⊕◯◯◯ VERY LOW	IMPORTANT
All-cause mortality in dialysis patients (follow-up: 13–57 months; evaluated with: phase angle cut-off 3.6° to <8°).											
12 [1,18,19,20,21,22,23,24,25,26,27,28,29]	observational studies	serious [a,b,f,g,m,n]	serious [j]	serious [d]	serious [e]	all possible residual confounding factors could reduce the observed effect	478	2220		⊕◯◯◯ VERY LOW	IMPORTANT
All-cause mortality in critically ill patients (follow-up: 8–28 days; evaluated with: phase angle cut-off 4.1° to <6°).											
5 [1,30,31,32,33,34]	observational studies	serious [a,b,o,p]	serious [j]	serious [d]	serious [q]	all possible residual confounding factors could reduce the observed effect	233	1172		⊕◯◯◯ VERY LOW	IMPORTANT
All-cause mortality in patients with liver disease (follow-up: 17–33 months; evaluated with: phase angle cut-off <4.4° to <5.2°).											

No. of studies	Certainty assessment						Effect			Certainty	Importance
	Study design	Risk of bias	Inconsistency	Indirectness	Imprecision	Other considerations	No. of events	No. of individuals	Rate (95% CI)		
4 [1,35,36,37,38]	observational studies	serious [a,o]	serious [j]	serious [d]	not serious	all possible residual confounding factors could reduce the observed effect	260	754		⊕⊕◯◯ LOW	IMPORTANT
Mortality in patients with hematopoietic cell transplantation (follow-up: mean 2 years; evaluated with: Mortality without relapse, and mortality with relapse. Phase angle standardized by age, sex and BMI)											
1 [39,r]	observational studies	serious [s]	not serious	serious [s]	not serious	all possible residual confounding factors could reduce the observed effect			event rate: 3.18% (1.23 to 8.27)	⊕⊕⊕◯ MODERATE	IMPORTANT
All-cause mortality in patients with heart failure (follow-up: 24–60 months; evaluated with: phase angle cut-off <4.2° to 5.5°).											
3 [40,41,42]	observational studies	serious [a,j]	serious [a,t]	serious [d]	not serious [u]	all possible residual confounding factors could reduce the observed effect	103	489		⊕⊕◯◯ LOW	IMPORTANT
All-cause mortality in COPD (follow-up: 4–23 months)											
1 [43]	observational studies	serious [v]	not serious	serious [d]	serious [w]	all possible residual confounding factors could reduce the observed effect	25	502		⊕⊕◯◯ LOW	IMPORTANT
All-cause mortality in HIV (follow-up: 20–33 months; assessed with: phase angle cut-off <5.3° to <5.6°)											
2 [44,45]	observational studies	serious [o,x]	not serious	serious [d]	serious [o,x]	all possible residual confounding factors could reduce the observed effect	39	332		⊕⊕◯◯ LOW	IMPORTANT
All-cause mortality in patients with sclerosis (follow-up: 16–35 months; assessed with: phase angle cut-off)											

No. of studies	Certainty assessment						Effect			Certainty	Importance
	Study design	Risk of bias	Inconsistency	Indirectness	Imprecision	Other considerations	No. of events	No. of individuals	Rate (95% CI)		
4 [46,47,48,49]	observational studies	serious [a,j,m,y]	serious [t]	serious [d]	serious [n]	all possible residual confounding factors could reduce the observed effect	200	371		⊕◯◯◯ VERY LOW	IMPORTANT
All-cause mortality in surgical patients (heart surgery) (evaluated with: phase angle cut-off 5.4°)											
1 [50]	observational studies	serious [z]	not serious	serious [d]	serious [aa]	all possible residual confounding factors could reduce the observed effect	9	325		⊕⊕◯◯ LOW	IMPORTANT
Mortality in elderly patients after hospital discharge (follow-up: mean 48 months; evaluated with: phase angle cut-off 4.6°).											
1 [51]	observational studies	serious [ab]	not serious	serious [d]	not serious	all possible residual confounding factors could reduce the observed effect		192	event rate: 0.78 % (0.64 to 0.95)	⊕⊕⊕◯ MODERATE	IMPORTANT
All-cause mortality in elderly patients (evaluated with: phase angle standardized by sex, age and BMI).											
1 [52]	observational studies	serious [ac]	not serious	serious [d]	serious [ad]	all possible residual confounding factors could reduce the observed effect	628	1307	event rate: 0.72 % (0.7 to 0.75)	⊕⊕◯◯ LOW	IMPORTANT
Mean length of stay in hospitalized patients (evaluated with: Length of stay [days]. Phase angle cut-off 5° in men and 4.6° in women).											
2 [53,54]	observational studies	serious [a]	not serious	serious [d]	not serious	all possible residual confounding factors could reduce the observed effect		1665		⊕⊕⊕◯ MODERATE	IMPORTANT
Mean length of stay in surgical patients (heart surgery) (evaluated with: Length of stay [days]. Phase angle standardized by sex and age)											

No. of studies	Certainty assessment						Effect			Certainty	Importance
	Study design	Risk of bias	Inconsistency	Indirectness	Imprecision	Other considerations	No. of events	No. of individuals	Rate (95% CI)		
1 [55]	observational studies	serious [ae]	not serious	serious [d]	serious [ae,af]	all possible residual confounding factors could reduce the observed effect		342		⊕⊕◯◯ LOW	IMPORTANT
Postsurgical complications in surgical patients (heart surgery) (follow-up: mean 1 month; assessed with: Postoperative risk, Society of Thoracic Surgeons postoperative risk evaluation model).											
1 [55]	observational studies	serious	not serious	serious [d]	serious	all possible residual confounding factors could reduce the observed effect		342		⊕⊕◯◯ LOW	IMPORTANT
Severity of disease in critically ill patients (evaluated with: APACHE II score. Phase angle cut-off: 5.1°).											
1 [56]	observational studies	serious [ag]	not serious	serious [d]	serious [ah]	all possible residual confounding factors could reduce the observed effect		95		⊕⊕◯◯ LOW	IMPORTANT
Probability of readmission in elderly patients after hospital discharge (follow-up: mean 48 months; evaluated with: phase angle cut-off 4.6°).											
1 [51]	observational studies	serious [ab]	not serious	serious [d]	not serious	all possible residual confounding factors could reduce the observed effect		192	event rate: 0.69 % (0.51 to 0.95)	⊕⊕⊕◯ MODERATE	IMPORTANT

BMI, body mass index.

Explanations

a. Heterogeneous populations and studies.

b. Small sample size in most studies.

c. Of the 48 studies included, only 42 confirm the correlation between phase angle and mortality.

d. Indirect evidence in patients who were possibly malnourished. Applicability may not be the same in other groups of patients studied in the guideline. Populations may differ in varying degrees of disease severity.

e. Wide confidence intervals in many studies, high variability between studies.

f. 3 studies do not describe how bioimpedance is conducted.

g. 3 studies do not describe how the phase angle is measured.

h. The phase angle cut-off is not stated in 5 studies.

i. The number of events is not indicated in 6 studies.

j. Methodological differences: different populations, different bioimpedance equipment, different phase angle cut-offs.

k. In one of the 15 studies, no significant association was found between phase angle and mortality.

l. The number of events is not indicated in 5 studies.

m. 2 studies do not describe how the phase angle is measured.

n. The number of events is not indicated in 1 study.

o. 1 study does not describe how the phase angle is measured.

p. 1 study does not state the phase angle cut-off.

q. In 2 of the 5 studies, no significant association was found between phase angle and mortality.

r. Phase angle was measured before transplantation.

s. The phase angle cut-off used (25th percentile) may enable comparison with other studies or populations.

t. In 1 of the studies, no association between phase angle and mortality was found.

u. In XXX 4 studies, no association between phase angle and mortality was found.

v. The phase angle cut-off is not stated.

w. The phase angle cut-off is not stated.

x. 1 of the studies does not describe how bioimpedance is used.

y. Two of the studies do not describe how bioimpedance is used.

z. The effect may have been underestimated by excluding elderly patients and patients with greater comorbidity.

aa. The association between phase angle and mortality loses significance in the multivariate analysis.

ab. Heterogeneous population.

ac. Heterogeneous population: outpatients and hospitalized patients.

ad. Continuous standardized phase angle is used. Sensitivity and specificity are lost when using a phase angle cut-off for predicting individual mortality.

ae. A low phase angle is associated with longer hospitalization time, but is not statistically significant.

af. Discrete differences in time spent hospitalized in the ward (median (IRQ): 14 (11–15) vs 12 (11–14) days, p=0.036), but no differences in time spent in the ICU (median (IRQ): 2 (2–4) vs. 2 (1–3) days, p=0.106).

ag. Phase angle has a modest correlation with the APACHE II score (r=-0.24). Phase angle values are affected by sepsis. The correlation between phase angle and APACHE II increases in patients without sepsis.

ah. The presence of sepsis affects the phase angle values. Small sample size of patients without sepsis (n=50), which may affect reproducibility of the results.

References

1. Garlini LM, Alves FD,Ceretta LB,Perry IS,Souza GC,Clausell NO. Phase angle and mortality: a systematic review. Eur J Clin Nutr; 2019 .

2. Büntzel J, Krauß T,Büntzel H,Küttner K,Fröhlich D,Oehler W,et al. Nutritional parameters for patients with head and neck cancer. Anticancer Res. ; 2012.

3. Davis MP, Yavuzsen T,Khoshknabi D,Kirkova J,Walsh D,Lasheen W,et al. Bioelectrical impedance phase angle changes during hydration and prognosis in advanced cancer. Am J Hosp Palliat Med. ; 2009.

4. Generoso SV, Rodrigues AM,Maia F,Armani B,Costa AC,Jansen AK,et al. 2014, 33:53., . Phase angle as prognostic indicator in surgical cancer patients. Nutr Cancer. ; 2014.

5. Gupta D, Lammersfeld CA,Burrows JL,Dahlk SL,Vashi PG,Grutsch JF,et al. 2004, 80:1634–8., . Bioelectrical impedance phase angle in clinical practice: implications for prognosis in advanced colorectal cancer. Am J Clin Nutr. ; 2004.

6. Gupta D, Lis CG,Dahlk SL,Vashi PG,Grutsch JF,Lammersfeld CA. 2004, 92:957–62., . Bioelectrical impedance phase angle as a prognostic indicator advanced pancreatic cancer. Br J Nutr. ; 2004.

7. Gupta D, Lammersfeld CA,Vashi PG,King J,Dahlk SL,Grutsch JF,et al. Bioelectrical impedance phase angle as a prognostic indicator in breast cancer. BMC Cancer. ; 2008.

8. Gupta D, Lammersfeld CA,Vashi PG,King J,Dahlk SL,Grutsch JF,et al. Bioelectrical impedance phase angle in clinical practice: implications for prognosis in stage IIIB and IV non-small cell lung cancer. BMC Cancer; 2009.

9. Hui D, Dev R,Pimental L,Park M,Cerana MA,Liu D,et al. Association between multi-frequency phase angle and survival in patients with advanced cancer. J Pain Symptom Manag. ; 2017.

10. Hui D, Bansal S,Morgado M,Dev R,Chisholm G,Bruera E. Cancer. Phase angle for prognostication of survival in patients with advanced cancer: preliminary findings. Cancer; 2014.

11. Lee SY, Lee YJ,Yang J,Kim CM,Choi WS. The association between phase angle of bioelectrical Impedance analysis and survival time in advanced cancer patients: preliminary study. Korean J Fam Med. ; 2014.

12. Santarpia L, Marra M,Montagnese C,Alfonsi L,Pasanisi F,Contaldo F. Prognostic significance of bioelectrical impedance phase angle in advanced cancer: preliminary observations. Nutrition. ; 2009.

13. Martin L, Lagergren J,Blomberg J,Johar A,Bosaeus I,Lagergren P. Phase angle as a prognostic marker after percutaneous endo- scopic gastrostomy (PEG) in a prospective cohort study. Scand J Gastroenterol. ; 2016.

14. Norman K, Wirth R,Neubauer M,Eckardt R,Stobäus N. 2015, 16:e17–22., . The bioimpedance phase angle predicts low muscle strength impaired quality of life, and increased mortality in old patients with cancer. J Am Med. ; 2015.

15. Sánchez-Lara K, Turcott JG,Juárez E,Guevara P,Núñez- Valencia C,Oñate-Ocaña LF,et al. Association of nutrition parameters including bioelectrical impedance and systemic inflammatory response with quality of life and prognosis in patients with advanced non-small-cell lung cancer: a prospective study. Nutr Cancer. ; 2012.

16. Skowronek P, Kuhberg M,Richter R,Chen F,Braicu EI,Sehouli J. Preoperative malnutrition as criteria for tumor resection com- pleteness and overall survival in patients with ovarian cancer: results of a prospective study. J Clin Oncol. ; 2014.

17. Toso S, Piccoli A,Gusella M,Menon D,Bononi A,Crepaldi G,et al. Altered tissue electric properties in lung cancer patients as detected by bioelectric impedance vector analysis. Nutrition. ; 2000.

18. Abad S, Sotomayor G,Vega A,Pérez de José A,Verdalles U,Jofré R,et al. The phase angle of the electrical impedance is a predictor of long-term survival in dialysis patients. Nefrologia.; 2011.

19. Beberashivili I, Azar A,Sinuani I,Shapiro G,Feldman L,Stav K,et al. Bioimpedance phase angle predicts muscle function, quality of life and clinical outcome in maintenance hemodialysis patients. Eur J Clin Nutr. ; 2014.

20. Caravaca F, Martínez del Viejo C,Villa J,Gallardo RM,Ferreira F. Hydration status assessment by multi-frequency bioimpedance in patients with advanced chronic kidney disease. Nefrologia.; 2011.

21. Chertow GM, Jacobs DO,Lazarus MJ,Lew NL,Lowrie EG. Phase angle predicts survival in hemodialysis patients. J Ren Nutr.; 1997.

22. Di Iorio B, Cillo N,Cirillo M,De Santo NG. Charlson comorbidity index is a predictor of outcomes in incident hemodialysis patients and correlates with phase angle and hospitalization. Int J Artif Organs. ; 2004.

23. A, Dumler,F. low bioimpedance phase angle predicts a higher mortality and lower nutritional status in chronic dialysis patients. J Phys Conf Ser. ; 2010.

24. Koh K, Wong H,Go K,Morad Z. 2011, 31:574–82., . Normalized bioimpedance indices are better predictors of outcome in perioneal dialysis patients. Perit Dial Int. ; 2011.

25. Maggiore Q, Nigrelli S,Ciccarelli C,Grimaldi C,Rossi GA,Michelassi C. Nutritional and prognostic correlates of bioimpedance indexes in hemodialysis patients. Kidney Int.; 1996.

26. Mushnick R, Fein PA,Mittman N,Goel N,Chattopadhyay J,Avram MM. 2003, 87:53–56., . Relationship of bioelectrical impedance parameters to nutrition and survival in peritoneal dialysis patients. Kidney Int Suppl. ; 2003.

27. Pupim LB, Caglar K,Hakim RM,Shyr Y,Ikizler TA. 2004, 66:2054–60., . Uremic malnutrition is a predictor of death independent of inflammatory status. Kidney Int.; 2004.

28. Rodrigues R, Oliveira B,Pedroso S,Azevedo JN,Azevedo P,Oliveira JP,et al. Predictive value of bioelectrical impedance analysis parameters in the mortality of patients on hemodialysis. Port J Nephrol Hypert. ; 2014.

29. Segall L, Mardare N,Ungureanu S,Busuioc M,Nistor I,Enache R,et al. Nutritional status evaluation and survival in Phase angle and mortality: a systematic review haemodialysis patients in one centre from Romania. Nephrol Dial Transplant. ; 2009.

30. Berbigier MC, Pasinato VF,Rubin BA,Moraes RB,Perry ID. Bioelectrical impedance phase angle in septic patients admitted to intensive care units. Rev Bras Ter Intens. ; 2013.

31. Da Silva TK, Berbigier MC,Rubin BA,Moraes RB,Souza GC,Perry ID. Phase angle as a prognostic marker in patients with critical illness. Nutr Clin Pract. ; 2015.

32. Díaz-De Los Santos M, Cieza J,Valenzuela R. Correlación entre índices de bioimpedancia elétrica y score Apache II en pacientes con shock séptico. Rev Med Hered. ; 2011.

33. Thibault R, Makhlouf A,Mulliez A,Gonzalez MC,Kekstas G,Kozjek N,et al. Fat-free mass at admission predicts 28-day mortality in intensive care unit patients: the international prospective observational study Phase Angle Project. Intensive Care Med. ; 2016.

34. Lee Y, Kwon O,Shin CS,Lee SM. Use of bioelectrical impedance analysis for the assessment of nutritional status in critically ill patients. Clin Nutr Res. ; 2015.

35. Belarmino G, Gonzalez MC,Torrinhas RS,Sala P,Andraus W,D'Albuquerque LA,et al. Phase angle obtained by bioelectrical impedance analysis independently predicts mortality in patients with cirrhosis. World J Hepatol. ; 2017.

36. Peres WAF, Lento DF,Baluz K,Ramalho A. Phase angle as a nutritional evaluation tool in all stages of chronic liver disease. Nutr Hosp; 2012.

37. Ruiz-Margáin A, Marcias-Rodrigues RU,Rios-Torres SL,Espinosa-Cuevas A,Duarte-Rojo A,Torre A. Phase angle as a nutritional marker related to prognosis in patients with liver cir- rhosis: a cut-off value for Mexican population. Gastroenterol. ; 2014.

38. Selberg O, Selberg D. Norms and correlates of bioimpedance phase angle in healthy human subjects, hospitalized patients, and patients with liver cirrhosis. Eur J Appl Physiol. ; 2002.

39. Urbain P, Birlinger J,Ihorst G,Biesalski H-K,Finke J,Bertz H. Body mass index and bioelectrical impedance phase angle as potentially modifiable nutritional markers are independent risk factors for outcome in allogeneic hematopoietic cell transplantation. Ann Hematol.; 2013.

40. Alves FD, Souza GC,Clausell N,Biolo A. Prognostic role of phase angle in hospitalized patients with acute decompensated heart failure. Nutrition; 2016.

41. Doesch C, Suselbeck T,Leweling H,Fluechter S,Haghi D,Schoenberg SO,et al. Obesity. 2010, 18:2326–32., . Bioimpedance analysis parameters and epicardial adipose tissue assessed by cardiac magnetic resonance imaging in patients with heart failure. Obesity; 2010.

42. Colín-Ramírez E, Castillo-Martínez L,Orea-Tejeda A,Vázquez- Durán M,Rodríguez AE,Keirns-Davis C. Bioelectrical impedance phase angle as a prognostic marker in chronic heart failure. Nutrition; 2012.

43. Maddocks M, Kon SS,Jones SE. Bioelectrical impedance phase angle relates to function, disease severity and prognosis in stable chronic obstructive pulmonary disease. Clin Nutr; 2015.

44. Schwenk A, Beisenherz A,Romer K,Kremer G,Salzberger B,Elia M. Phase angle from bioelectrical impedance analysis remains an independent predictive marker in HIV-infected patients in the era of highly active antiretroviral treatment. Am J Clin Nutr. ; 2000.

45. Ott M, Fischer H,Polat H,Helm EB,Frenz M,Caspary WF,et al. Bioelectrical impedance analysis as a predictor of survival in patients with human immunodeficiency virus infection. J Acquir Immune Defic Syndr Hum Retrovirol. ; 1995.

46. Robeau V, Blasco H,Maillot F,Corcia P,Praline J. Nutritional assessment of amyotrophic lateral sclerosis in routine practice: value of weighing and bioelectrical impedance analysis. Muscle Nerve. ; 2015.

47. Desport JC, Marin B,Funalot B,Preux PM,Couratier P. Phase angle is a prognostic factor for survival in amyotrophic lateral sclerosis. Amyotroph Lateral Scler. ; 2008.

48. Krause L, Becker MO,Brueckner CS,Bellinghausen CJ,Becker C,Schneider U,et al. Nutritional status as marker for disease activity and severity predicting mortality in patients with systemic sclerosis. Ann Rheum Dis. ; 2010.

49. Marin B, Desport JC,Kajeu P,Jesus P,Nicolaud B,Nicol M,et al. lteration of nutritional status at diagnosis is a prognostic factor for survival of ALS patients. AJ Neurol Psychiatry. ; 2011.

50. Visser M, van Venrooij LM,Wanders DC,de Vos R,Wisselink W,van Leeuwen PA,de Mol BA. Clin Nutr. 2012 Dec, 10.1016/j.clnu.2012.05.002., 31(6):981-6.,doi:. The bioelectrical impedance phase angle as an indicator of undernutrition and adverse clinical outcome in cardiac surgical patients. Clin Nutr; 2012.

51. Buscemi S, Batsis JA,Parrinello G,et al. Nutritional predictors of mortality after discharge in elderly patients on a medical ward. Eur J Clin Invest; 2016.

52. Genton L, Norman K,Spoerri A,et al. R. Bioimpedance-derived phase angle and mortality among older people. ejuvenation Res ; 2017.

53. Kyle UG, Genton L,Pichard C. Low phase angle determined by bioelectrical impedance analysis is associated with malnutrition and nutritional risk at hospital admission. Clin Nutr ; 2013.

54. Guerra RS, Fonseca I,Pichel F,et al. Usefulness of six diagnostic and screening measures for undernutrition in predicting length of hospital stay: a comparative analysis. J Acad Nutr Diet; 2015.

55. Ringaitiene D, Gineityte D,Vicka V,et al. Malnutrition assessed by phase angle determines outcomes in low-risk cardiac surgery patients. Clin Nutr ; 2016.

56. da Silva TK, Berbigier MC,Rubin Bde A,et al. Phase angle as a prognostic marker in patients with critical illness. Nutr Clin Pract; 2015.

Topic 6: Muscle imaging – Ultrasound

Author(s): Pilar Matía Martín

Question: Clinical progression patients with disease-related malnutrition (or at risk of malnutrition) with low quantity or quality of skeletal muscle mass measured by ultrasound during short- to medium-term follow-up.

Setting: General population is not included. Chronic or acute illnesses are included.

No. of studies	Certainty assessment						Impact	Certainty	Importance
	Study design	Risk of bias	Inconsistency	Indirectness	Imprecision	Other considerations			
Re-admission or death after hospitalization (follow-up: 3–12 months; evaluated with: right rectus femoris cross-sectional area/height^2 and quadriceps thickness)									
2 [1,2]	observational studies	serious [a,b,c,d]	serious [e]	serious [f]	serious [g]	all possible residual confounding factors could reduce the observed effect	1. Reason for admission: Exacerbation of respiratory pathology: adjusted OR for each unit increase in rectus femoris cross-sectional area/height^2 (cm^2/m^2): 0.46 (95% CI 0.22–0.95); p=0.035 (N=191). Follow-up: 12 months. No nutritional assessment data. No reliability data.<br><br>2. Various reasons for admission (mainly pneumonia): RR if quadriceps thickness ≤1.2 cm: 1.2; CI 95% 0.9–3.5; p=0.08 (model adjusted for age and sex). RR if quadriceps thickness ≤1.2 cm in bedridden patients: 1.34 (95% CI 1.02–1.75); p=0.04 (unadjusted model); N=100. 3-month follow-up. No nutritional assessment data except for CC and hand dynamometry. With reliability data.	⨁◯◯◯ VERY LOW	CRITICAL
Discharge to other center or in-hospital death in critical surgical patients (assessed with: Rectus femoris cross-sectional area (unadjusted for sex); sarcopenia if <5.2 cm^2 (ROC curve: frailty discrimination). Evaluated at admission to ICU).									
1 [3]	observational studies	serious [h]	not serious	serious [a,i]	serious [g]	all possible residual confounding factors could reduce the observed effect	Adjusted OR associated with sarcopenia 7.49; 95% CI 1.47–38.24; p=0.015 (N=102). Follow-up: hospital stay. Overall prevalence of malnutrition in the sample 37.3% (Short Nutritional Assessment). With reliability data.	⨁⨁◯◯ LOW	CRITICAL
In-hospital mortality in critically ill patients (assessed with: rectus femoris + vastus intermedius thickness (not adjusted for sex) in the first 48 hours/Percent change in muscle thickness in the mid-arm and mid-thigh [1 to 3 days ICU stay]).									
2 [4,5]	observational studies	very serious [a,d,j,k]	not serious	serious [a,i]	not serious	all possible residual confounding factors could reduce the observed effect	4. Adjusted OR (SOFA) by muscle thickness: 0.11, 95% CI 0.02–0.74; p=0.002 (N=59). Follow-up: hospital stay. No reliability data. High risk of malnutrition (NUTRIC score): 25.5% survived and 58.3% died. The accuracy of the model for predicting mortality did not improve with the inclusion of NUTRIC score; collinearity with SOFA.<br><br>5. Unadjusted HR of death for a decrease ≥ 6.59% of arm muscle at day 3 of ICU stay: 7.3; 95% CI 1.5–34.1; p=0.012. Unadjusted HR of death for a decrease ≥ 5.20% of thigh muscle at day 3 of ICU stay: 8.1; 95% CI 1.7–37.9; p=0.008 (N=70). Follow-up: hospital stay. No nutritional assessment. No reliability data.	⨁⨁◯◯ LOW	CRITICAL
Mortality in ICU (heterogeneous group of critically ill patients) (assessed with: Muscle echogenicity [anterior mid-forearm, mid-biceps and thigh], Heckmatt four-point visual score).									

No. of studies	Certainty assessment						Impact	Certainty	Importance
	Study design	Risk of bias	Inconsistency	Indirectness	Imprecision	Other considerations			
1 [6]	observational studies	very serious [d,l,m]	not serious	serious [a,i]	very serious [c,n]	all possible residual confounding factors could reduce the observed effect	Increased echogenicity was associated with higher mortality: 42% vs 12%, p=0.004 (unadjusted). In the adjusted model (not shown) statistical significance was lost (N=67). Follow-up: stay in ICU. No nutritional assessment data. No reliability data.	⊕◯◯◯ VERY LOW	CRITICAL
Mortality in patients with decompensated cirrhosis or with any episode of decompensation in the previous year (follow-up: 12 months; evaluated with: Psoas muscle diameter/height (mm/m) and psoas muscle index (pi*psoas radius^2/height^2 [cm^2/m^2]).									
1 [7]	observational studies	very serious [a,d,o]	not serious	serious [a,i]	serious [c]	all possible residual confounding factors could reduce the observed effect	Adjusted HR for death: psoas muscle diameter/height (mm/m) HR 0.825, 95% CI 0.701–0.973, p=0.022; and for psoas muscle index (cm^2/m^2) HR 0.930, 95% CI 0.876–0.987, p=0.017 (N=54). Median follow-up: 12 months. No nutritional assessment. No complete reliability data.	⊕◯◯◯ VERY LOW	CRITICAL
Probability of discharge in critically ill patients (heterogeneous group) (assessed with: Muscle echogenicity [mid-biceps, anterior mid-forearm, and mid-thigh], Heckmatt four-point visual score).									
1 [6]	observational studies	very serious [d,l,m]	not serious	serious [a,i]	serious [g]	strong association. All possible residual confounding factors could reduce the observed effect	Increased echogenicity was associated with a lower likelihood of discharge: adjusted OR 0.42; 95% CI 0.2–0.86), p=0.02 (N=67). Follow-up: hospital stay. No nutritional assessment data. No reliability data.	⊕⊕◯◯ LOW	IMPORTANT
Mean hospital stay due to exacerbation of respiratory illness (follow-up: 12 months; evaluated with: Right rectus femoris transverse area/height^2 [quartiles, Q]).									
1 [1]	observational studies	very serious [a,d]	not serious	serious [f]	very serious [c]	all possible residual confounding factors could reduce the observed effect	28.1 (SD 33.9) days in Q1, 11.9 (SD 19.0) in Q2, 12.9 (SD 23.1) in Q3 and 12.2 (SD 23.5) in Q4; p=0.007 (N=191). Follow-up: hospital stay. No nutritional assessment data. No reliability data.	⊕◯◯◯ VERY LOW	IMPORTANT
Mean hospital stay in critically ill surgical patients (assessed with: Rectus femoris cross-sectional area (adjusted for sex); sarcopenia if <5.2 cm^2 (ROC curve: frailty discrimination). Evaluated at admission to ICU.)									
1 [3]	observational studies	serious [h]	not serious	serious [a,i]	not serious	all possible residual confounding factors could reduce the observed effect	Hospital stay in the presence of sarcopenia: adjusted IRR 1.37; 95% CI 1.19–1.58; p<0.001. Hospital stay by sex-adjusted rectus femoris cross-sectional area: adjusted IRR 0.91; 95% CI 0.88–0.95; p<0.001 (N=102). Follow-up: hospital stay. Overall prevalence of malnutrition in the sample: 37.3% (Short Nutritional Assessment). With reliability data	⊕⊕⊕◯ MODERATE	IMPORTANT
Mean ICU stay (assessed with: Rectus femoris cross-sectional area (adjusted for sex); sarcopenia if <5.2 cm^2 (ROC curve: frailty discrimination). Evaluated at admission to ICU. Percent change in muscle thickness the mid-arm and mid-thigh (1 to 3 days of ICU stay)/Rectus femoris and vastus intermedius thickness. Different times during ICU stay)									
3 [3,5,8]	observational studies	very serious [a,d,h,k,l,p]	serious [e]	serious [a,i]	very serious [c,q,r]	all possible residual confounding factors could reduce the observed effect	3. ICU stay in the presence of sarcopenia: adjusted IRR 1.13, 95% CI 0.90–1.43; p=0.29 (N=102). Follow-up: hospital stay. Overall prevalence of malnutrition in the sample 37.3% (Short Nutritional Assessment). With reliability data.<br><br>5.Unadjusted OR of prolonged ICU stay (≥2 weeks) for a decrease ≥6.59% of upper arm muscle at day 3 of ICU stay: 3.8; 95% CI 0.90–16.1; p=0.067. Unadjusted OR of prolonged ICU stay (≥2 weeks) for a decrease ≥5.20% of thigh muscle at day 3 of ICU stay: 3.0; 95% CI 0.72–12.4); p=0.132 (N=70). Follow-up: ICU stay. No nutritional assessment. No reliability data.<br><br>8. Inverse correlation between right and left baseline muscle thickness and mean ICU stay	⊕◯◯◯ VERY LOW	IMPORTANT

No. of studies	Certainty assessment						Impact	Certainty	Importance
	Study design	Risk of bias	Inconsistency	Indirectness	Imprecision	Other considerations			
							(p<0.0001); the value of the coefficient is not stated (N=101). Follow-up: ICU stay. No nutritional assessment. Some reliability data.		
ICU-free days (heterogeneous group of critically ill patients) (assessed with: Muscle echogenicity (mid-biceps, anterior mid-forearm, and mid-thigh), Heckmatt four-point visual score).									
1 [6]	observational studies	very serious [d,l,m]	not serious	serious [a,i]	very serious [s,t]	all possible residual confounding factors could reduce the observed effect	Increased echogenicity was associated with fewer ICU-free days: 3 (IQR 0–15) vs 16 (IQR 9.3–19.3); p=0.0002 (unadjusted). In the adjusted model (not shown) statistical significance was lost (N=67). Follow-up: hospital stay. No nutritional assessment data. No reliability data.	⨁◯◯◯ VERY LOW	IMPORTANT
Duration of mechanical ventilation in ICU (critical patients with sepsis) (evaluated with: Percentage change of muscle thickness in the mid-arm and mid-thigh [1 to 3 days of ICU stay])									
1 [5]	observational studies	serious [a,d,k]	not serious	serious [a,i]	very serious [g,t]	all possible residual confounding factors could reduce the observed effect	Unadjusted OR of prolonged mechanical ventilation in ICU (≥2 weeks) for a decrease ≥6.59% of arm muscle at day 3 of ICU stay: 4.2; 95% CI 0.65–27.6); p=0.131. Unadjusted OR of prolonged mechanical ventilation in ICU (≥2 weeks) for a decrease ≥5.20% of thigh muscle at day 3 of ICU stay: 9.7; 95% CI 1.01–92.4); p=0.049 (N=70). Follow-up: ICU stay. No nutritional assessment. No reliability data.	⨁◯◯◯ VERY LOW	IMPORTANT
Physical activity after hospital discharge for COPD exacerbation (follow-up: 4 weeks; assessed with: Rectus femoris cross-sectional area in the first 48 hours after admission [curvilinear transducer]).									
1 [9]	observational studies	very serious [b,c,j]	not serious	serious [f]	very serious [u,v]	all possible residual confounding factors could reduce the observed effect	Correlation between rectus femoris cross-sectional area and physical activity r=0.75, p=0.006 (N=16). Follow-up: 4 weeks. Without nutritional assessment. With reliability data.	⨁◯◯◯ VERY LOW	IMPORTANT
Health-related quality of life (physical dimension) in patients with COPD (assessed with: rectus femoris echogenicity).									
1 [10]	observational studies	very serious [j,w]	not serious	serious [i]	serious [s,x]	all possible residual confounding factors could reduce the observed effect dose-response gradient	Adjusted beta coefficient (multiple linear regression) between echogenicity (undefined units) and the physical dimension of quality of life: -0.312; p=0.028 (N=50) (note: echogenicity was not associated with BMI). Follow-up: cross-sectional study. No nutritional assessment.	⨁⨁◯◯ LOW	IMPORTANT
Health-related quality of life (physical dimension) in critically ill patients with brain trauma (follow-up: 3 months; assessed with: Quadriceps muscle layer thickness [sum of rectus femoris and vastus intermedius thickness] at hospital discharge).									
1 [11]	observational studies	serious [d,j]	not serious	serious [a,i,p]	serious [c,u]	all possible residual confounding factors could reduce the observed effect	Correlation between muscle thickness at discharge and quality of life-physical dimension- (SF36vs): r=0.536; p=0.010 (N=22). Follow-up 3 months. Malnutrition at discharge (SGA B or C): 44%. No reliability data.	⨁⨁◯◯ LOW	IMPORTANT
Global functionality in critically ill patients with brain trauma (follow-up: 3 months; assessed with: Quadriceps muscle layer thickness [sum of rectus femoris and vastus intermedius thickness] at hospital discharge).									
1 [11]	observational studies	serious [d,j]	not serious	serious [a,i,p]	serious [c,u]	all possible residual confounding factors could reduce the observed effect	Correlation between muscle thickness at discharge and functionality (Extended Glasgow Outcome Scale [GOS-E])-: r=0.595; p=0.003 (N=23). Follow-up: 3 months. Malnutrition at discharge (SGA B or C): 44%. No reliability data.	⨁⨁◯◯ LOW	IMPORTANT

No. of studies	Certainty assessment						Impact	Certainty	Importance
	Study design	Risk of bias	Inconsistency	Indirectness	Imprecision	Other considerations			
Poor clinical prognosis (death, emergency visit, readmission) after ICU admission for sepsis (follow-up: 90 days; evaluated with: Percentage change in muscle thickness in the mid-arm and mid-thigh [1 to 3 days of ICU stay])									
1 [5]	observational studies	serious [a,d,k]	not serious	serious [a,i]	very serious [t,y]	all possible residual confounding factors could reduce the observed effect	Patients with poor prognosis 90 days after ICU admission presented greater thigh muscle thickness loss after 3 days in ICU: 5.7%; IQR 0.8–14.4 vs 2.1%; IQR -5.0–7.9; p<0.001 (N=70). Follow-up: 90 days. No nutritional assessment. No reliability data.	⊕◯◯◯ VERY LOW	CRITICAL
Admission for decompensation in patients with decompensated cirrhosis or with an episode of decompensation in the previous year (follow-up: 12 months; evaluated with: Psoas muscle diameter/height (mm/m) and psoas muscle index (pi*psoas radius^2/height^2 [cm^2/m^2]).									
1 [7]	observational studies	very serious [a,d,o]	not serious	serious [a,i]	serious [c]	all possible residual confounding factors could reduce the observed effect	Adjusted HR of admission for cirrhosis decompensation: Psoas muscle diameter/height (mm/m) HR 0.16; 95% CI 0.05–0.50; p=0.002; and of Psoas muscle index (cm^2/m^2) HR 0.58; 95% CI 0.42–0.81; p=0.002 (N=54). Median follow-up: 12 months. No nutritional assessment. No complete reliability data.	⊕◯◯◯ VERY LOW	IMPORTANT

ROC, receiver operating characteristic; ICU, intensive care unit; IRR, incidence rate ratio; RR, relative risk; SGA, subjective global assessment.

Explanations

a. Single-center study (in at least one of the studies).

b. Insufficient time in any study to observe the event.

c. Limited adjustment for relevant variables in the estimations.

d. Inter-observer reliability is not defined in at least one of the studies.

e. Variability in estimates.

f. One study with patients with respiratory illness.

g. Wide confidence intervals

h. The cut-off for defining sarcopenia would need to be validated.

i. Generalization of results cannot be guaranteed.

j. Very small sample (in at least one of the studies)

k. Unclear sample selection (in at least one of the studies).

l. It is not clearly defined how the measurement of the thickness of the muscles involved is carried out

m. Patients with and without ultrasound may not be comparable.

n. Adjusted mortality risk not shown.

o. Difficult measurement in more than 25% of the sample (high or low waist circumference).

p. Patients may not be comparable for sequential study.

q. Some studies do not provide adjusted data.

r. Different measures of strength of association are used.

s. Confidence intervals of the estimate are not shown.

t. Adjusted data are not shown

u. Correlation is only shown for exposure and clinical event.

v. Units of measurement of physical activity assessed by accelerometry are not defined.

w. Cross-sectional study.

x. Stable patients, probably at lower risk of malnutrition.

y. Only descriptive data are shown.

References

1. Greening NJ, Harvey-Dunstan TC,Chaplin EJ,Vincent EE,Morgan MD,Singh SJ,et al. Bedside assessment of quadriceps muscle by ultrasound after admission for acute exacerbations of chronic respiratory disease. Am J Respir Crit Care Med.; 2015.

2. Guerreiro AC, Tonelli AC,Orzechowski R,Dalla Corte RR,Moriguchi EH,de Mello RB. Bedside Ultrasound of Quadriceps to Predict Rehospitalization and Functional Decline in Hospitalized Elders. Front Med. ; 2017.

3. Mueller N, Murthy S,Tainter CR,Lee J,Riddell K,Fintelmann FJ,et al. Can sarcopenia quantified by ultrasound of the rectus femoris muscle predict adverse outcome of surgical intensive care unit patients as well as frailty? a prospective, observational cohort study. Ann Surg; 2016.

4. Galindo Martín CA, Ubeda Zelaya RDC,Monares Zepeda E,Lescas Méndez OA. ROUNDS Studies: Relation of OUtcomes with Nutrition Despite Severity-Round One: Ultrasound Muscle Measurements in Critically Ill Adult Patients. J Nutr Metab.; 2018.

5. Hadda V, Kumar R,Khilnani GC,Kalaivani M,Madan K,Tiwari P,et al. Trends of loss of peripheral muscle thickness on ultrasonography and its relationship with outcomes among patients with sepsis. J intensive care; 2018.

6. Kelmenson DA, Quan D,Moss M. What is the diagnostic accuracy of single nerve conduction studies and muscle ultrasound to identify critical illness polyneuromyopathy: a prospective cohort study. Crit Care.; 2018.

7. Hari A, Berzigotti A,Štabuc B,Caglevič N. Muscle psoas indices measured by ultrasound in cirrhosis - Preliminary evaluation of sarcopenia assessment and prediction of liver decompensation and mortality. Dig liver Dis Off J Ital Soc Gastroenterol Ital Assoc Study Liver. ; 2019.

8. Gruther W, Benesch T,Zorn C,Paternostro-Sluga T,Quittan M,Fialka-Moser V,et al. Muscle wasting in intensive care patients: ultrasound observation of the M. quadriceps femoris muscle layer. J Rehabil Med.; 2008.

9. Mandal S, Suh E,Thompson A,Connolly B,Ramsay M,Harding R,et al. Comparative study of linear and curvilinear ultrasound probes to assess quadriceps rectus femoris muscle mass in healthy subjects and in patients with chronic respiratory disease. BMJ open Respir Res.; 2016.

10. Ye X, Wang M,Xiao H. Echo intensity of the rectus femoris in stable COPD patients. Int J Chron Obstruct Pulmon Dis. 2017.

11. Chapple L-AS, Deane AM,Williams LT,Strickland R,Schultz C,Lange K,et al. Longitudinal changes in anthropometrics and impact on self-reported physical function after traumatic brain injury. Crit care Resusc J Australas Acad Crit Care Med. ; 2017.

Topic 6: Muscle imaging – Computed tomography

Question: Disease progression associated with low muscle mass measured by computed tomography

Setting: General population is not included.

Author(s):

No. of studies	Certainty assessment						Impact	Certainty	Importance
	Study design	Risk of bias	Inconsistency	Indirectness	Imprecision	Other considerations			
Mortality in patients with cytoreductive surgery and hyperthermic intraperitoneal chemotherapy based on low skeletal muscle mass (SMM) (evaluated with: L3 skeletal muscle index [SMI] [cm^2/m^2]).									
4 [1,2,3,4]	observational studies	serious [a]	not serious	serious [b]	serious [c]	all possible residual confounding factors could reduce the observed effect	1. Median survival 73.3 months without low SMM vs 57.2 months with low SMM (p=0.05) (N=115) pseudomyxoma (N=82) and peritoneal mesothelioma (N=33). Median follow-up: 18.1 months. Post-surgical mortality: 6.2% with low SMM vs 0% without low SMM (p=0.069).<br><br>2. Median survival: 50 months without low SMM, 59 months with low SMM (p=0.648); N=214, colon cancer. Median follow-up: 24 months.<br><br>3. Mortality at 30 days: 5% with low SMM vs 2% without low SMM (p=0.06); N=97, colorectal cancer. 30-day follow-up.<br><br>4. In-hospital mortality/30 days: 2.2% with low SMM vs 2.6% without low SMM (p=0.646); N=206, colorectal cancer. Follow-up: 30 days.	⨁⨁◯◯ LOW	CRITICAL
Mortality in patients with breast cancer based on low SMM (evaluated with: L3 SMI [cm^2/m^2]).									
3 [5,6,7,8]	observational studies	serious [d]	serious [e]	not serious	serious [f]	all possible residual confounding factors could reduce the observed effect	5. Mortality with normal vs low SMM: HR 0.3 (0.1–0.99; p=0.05); N=119, non-metastatic breast cancer with aggressive features before chemotherapy treatment. Median follow-up: 52.4 months.	⨁⨁◯◯ LOW	CRITICAL

No. of studies	Certainty assessment						Impact	Certainty	Importance
	Study design	Risk of bias	Inconsistency	Indirectness	Imprecision	Other considerations			
							6. HR 1.41 (1.18–1.69); N=3241, non-metastatic/stage II or III. Median follow-up 6 years.<br>7. aHR per unit increase in SMI 1.02 (1.00–1.04; p=0.0309); N=129, non-metastatic with neoadjuvant chemotherapy; responding cases/non-responding controls. Median follow-up: 7.74 years.<br>8. aHR 0.98 (0.60–1.58; p=0.923); N=166, metastatic; 1 line of palliative chemotherapy. Median follow-up: 22 months.		
Mortality in patients with head and neck cancer based on low SMM (evaluated with: L3 SMI [cm²/m²]).									
2 [9,10]	observational studies	serious [a,c]	serious [g]	serious [h]	serious [f]	all possible residual confounding factors could reduce the observed effect	9. HR 2.95 (1.4–6.0); p=0.003; N=113, epidermoid head and neck cancer in non-underweight patients. Median follow-up: not specified.<br>10. Median survival: 10.58 months without low SMM vs 13.34 months with low SMM (log-rank test p=0.29, unadjusted). N=85, head and neck cancer with curative treatment in patients over 69 years. MRI was used in some cases for the assessment of SMM (N not specified).	⨁◯◯◯ VERY LOW	CRITICAL
Mortality in patients with colorectal cancer based on low SMM (evaluated with: L3 SMI [cm²/m²]).									
15 [11,12,13,14,15,16,17,18,19,20,21,22,23,24,25]	observational studies	serious [d,i,j,k,l]	not serious [m]	serious [b]	serious [f,n]	all possible residual confounding factors could reduce the observed effect	11. aHR 1.45 (1.16–1.84; p=0.002); N=968, colorectal cancer stages I-III with preoperative CT. Median follow-up: 5.2 years.<br>12. aHR: 1.49 (1.04–2.15; p=0.03); N=217, advanced colorectal cancer refractory to chemotherapy; ECOG 0–1-	⨁⨁◯◯ LOW	CRITICAL

No. of studies	Certainty assessment						Impact	Certainty	Importance
	Study design	Risk of bias	Inconsistency	Indirectness	Imprecision	Other considerations			
							. Meidan follow-up not specified.<br><br>13. aHR 1.50 (1.04–2.18 ; p=0.031); N=650, colorectal cancer pre-surgery, no metastases, non-palliative treatment. Median follow-up: not specified.<br><br>14. Low SMM (Z score <0) was not associated with survival (data not shown). When the Fong prognostic score was excluded in the multivariate model: aHR 1.37 (1–1.89; p=0.0529). The combination of low SMM and high CRP was associated with increased mortality. N=97 pre-surgery: resection of hepatic metastases of colorectal cancer. Median follow-up:73.6 months.<br><br>15. aHR 6.059 (2.069–17.747); p=0.001; N=65 ≥65 years, colorectal cancer with chemoradiotherapy. Median follow-up: 106.8 months.<br><br>16. aHR 1.27 (1.09–1.48); N=3262 invasive colorectal cancer, stages I–III with surgery. Median follow-up: 6 years.<br><br>17. aHR 1.28 (1.10–1.53); N=2470 invasive colorectal cancer, stages I–III with surgery. Median follow-up: 6 years.<br><br>18. aHR 1.70 (1.25–2.31); p<0.001; N=805 colorectal cancer with elective surgery. Median follow-up: 47 months.<br><br>19. aHR 2270 (1.147–4.494); p=0.019; N=220 colorectal cancer, stages I-III with curative surgery. Median follow-up: 41.4 months.<br><br>20. aHR 1.74 (0.99–3.03); p=0.053; N=77, colorectal		

No. of studies	Certainty assessment						Impact	Certainty	Importance
	Study design	Risk of bias	Inconsistency	Indirectness	Imprecision	Other considerations			
							cancer stage IV. Follow-up: at least 2.5 years.<br><br>21. aHR 2.53 (1.60–4.01); p<0.001; N=196 surgical resection of hepatic metastases of colorectal cancer. Median follow-up: 29 months.<br><br>22. aOR 43.30 (2.74–685.2); p=0.007; N=310 intestinal resection. Follow-up: 30 days post-surgery.<br><br>23. aHR during disease progression and treatment 1.07 (0.84–1.35); 1.01 (0.87–1.36); 1.20 (0.96–1.54); N=450 systemic palliative treatment. Median follow-up not specified.<br><br>24. aHR 1.65 (0.85–3.18); p=0.138; N=67 advanced cancer before treatment with chemotherapy. Median survival: 17.5 months.<br><br>25. No association (p=0.917); N=182 advanced cancer before chemotherapy. Median follow-up: 23.2 months.		
Mortality in patients with biliopancreatic cancer based on low SMM (evaluated with: L3 SMI [cm^2/m^2]).									
10 [26,27,28,29,30]	observational studies	not serious [d,i,j,o]	serious [g]	serious [b]	serious [n]	all possible residual confounding factors could reduce the observed effect	26. aHR 1.37 (1.01–1.87); p=0.045; N=82 FOLFIRINOX. In locally advanced cancer, aHR 3.85 (1.34–11.04); p=0.012. In metastatic cancer, no association. Mean follow-up not specified.<br><br>27. aHR 1.79 (1.20–2.65); N=180 perioperative/curative. Median follow-up not specified.<br><br>28. aHR 2.04 (0.93–4.49); p=0.07; N=107 pre-surgery, 28% with benign disease. Median follow-up: 15 months.	⊕⊕◯◯ LOW	CRITICAL

No. of studies	Certainty assessment						Impact	Certainty	Importance
	Study design	Risk of bias	Inconsistency	Indirectness	Imprecision	Other considerations			
							29. (Lowest tertile of SMM): no relation; data not shown; N=199 pre-surgery. Median Follow-up: 57.7 months.<br>30. Meta-analysis: aHR 1.49 (1.27–1.74); p<0.00001; I2=5%, p=0.39; N=1239 over 60 years old. Follow-up not recorded in all studies.		
Mortality in patients with hepatocellular carcinoma based on low SMM (evaluated with: L3 SMI [cm^2/m^2]).									
12 [31,32,33,34,35,36,37,38,39,40,41,42]	observational studies	serious [i,j,k,l,p]	serious [e]	not serious	serious [f]	all possible residual confounding factors could reduce the observed effect	31. (Survival): 13.7 low SMM vs 18.5 months (p=0.16). Multivariate: no relationship if >2 prognostic factors. If <3 prognostic factors, low SMM mortality: aHR 1.6 (1.0–2.4); p=0.047; N=214 advanced, pre-treatment with sorafenib. Follow-up not specified.<br>32. aHR 2.37 (1.28–4.39); p=0.006; N=92 at diagnosis; cirrhosis. Follow-up not specified.<br>33. (Survival <70 years): log rank test p=ns. Low mortality when low SMM in patients ≥70 years: aHR 2,544 (1,206–5.5865); p=0.0199; SMM evaluated before curative surgery (N=296). Follow-up not specified.<br>34. aHR 1.52 (1.18–1.96); p=0.001; N=1257 different stages-. Follow-up not specified.<br>35. aHR 3.19 (1.28–7.96); p=0.013; N=109 before surgery. Median follow-up: 21.23 months.<br>36. aHR 2.94 (0.82–10.58); p=0.09; N=92-after curative treatment with surgery or radiofrequency. Median follow-up: 34 months.<br>37. aHR 3.756 (1.778–7.932); p=0.001; N=90 after curative treatment	⨁⨁◯◯ LOW	CRITICAL

No. of studies	Certainty assessment						Impact	Certainty	Importance
	Study design	Risk of bias	Inconsistency	Indirectness	Imprecision	Other considerations			
							with surgery or radiofrequency. Median follow-up: 22.5 months.<br><br>38. aHR 1.96 (1.06–3.74); p=0.031; N=190 before liver resection. Follow-up not specified.<br><br>39. aHR 0.90 (0.84–0.96); p=0.002; N=186 before liver resection. Follow-up not specified.<br><br>40. HR 1.20 (0.94–1.54); p=0.417; N=278 treatment with sorafenib. Median follow-up: 54.9 months.<br><br>41. aHR 1.153 (0.538–2.474); p=0.715; N=82 treatment with sorafenib. Follow-up not specified.<br><br>42. aHR 1.63 (1.05–2.53); p=0.03; N=96 treatment with sorafenib. Follow-up not specified.		
Mortality in patients with gastric cancer based on low SMM (evaluated with: L3 SMI [cm^2/m^2]).									
14 [43,44]	observational studies	serious [d,q,r]	not serious	serious [b,s]	not serious	publication bias is strongly suspected. Strong association. All possible residual confounding factors could reduce the observed effect[t]	43. Meta-analysis (9 studies): aHR 1.81 (1.52–2.14); I2=32%, N=2421. Tumor-specific HR 1.58 (1.36–1.84); I2=0%, N=1702. Follow-up: 30–64 months. Only 3 studies adjusted for BMI and 2 for albumin.<br><br>44. Meta-analysis (11 studies): aHR 1.89 (1.68–2.12); I2 36% -N=4005-. Follow-up: 12–62.3 months. No nutritional assessment data. No adjustment for variables related to nutritional status is stated.	⊕⊕⊕◯ MODERATE	CRITICAL
Mortality in patients with esophageal cancer based on low SMM (evaluated with: L3 SMI [cm^2/m^2]).									
6 [45]	observational studies	serious [d,i,q]	serious [u]	serious [v]	not serious	all possible residual confounding factors could reduce the observed effect	Meta-analysis: HR death: 1.70 (1.33–2.17); p<0.0001; I2=48.1%, N=1,273. Follow-up: 20–50 months. No association with early mortality (in-hospital, 30 days after surgery). 4 studies: OR	⊕⊕◯◯ LOW	CRITICAL

No. of studies	Certainty assessment						Impact	Certainty	Importance
	Study design	Risk of bias	Inconsistency	Indirectness	Imprecision	Other considerations			
							1.18 (0.47–2.96); p=0.718; I2=0.0%; N=912.		
Mortality in lung cancer patients based on low SMM (evaluated with: L3 total psoas index [TPI]–SMI [cm^2/m^2]).									
6 [46,47]	observational studies	serious [i,q,w]	serious [x]	serious [b,y]	not serious	all possible residual confounding factors could reduce the observed effect	46. Meta-analysis (3 studies): HR 2.31 (1.26–4.24); I2=69%, N=636. Follow-up: 35.5–59 months. No nutritional assessment data.<br><br>47. Meta-analysis (6 studies; surgically treated non-small cell lung cancer): RR 2.85 (1.67–4.86); p<0.001; I2=64.5%, N=1213. Follow-up: 26.3–61 months. No nutritional assessment data.	⊕⊕◯◯ LOW	CRITICAL
Mortality in patients with renal cancer based on low SMM (evaluated with: L3 SMI [cm^2/m^2]).									
6 [48]	observational studies	serious [a,q,z]	not serious	serious [b,y]	serious [d]	all possible residual confounding factors could reduce the observed effect	Meta-analysis (metastatic cancer; different cut-offs): HR 1.48 (1.08–2.03); I2=28%, N=559. Follow-up: 13–51 months. No nutritional assessment data.	⊕⊕◯◯ LOW	CRITICAL
Mortality in patients with hematologic cancer based on low SMM (evaluated with: L3 SMI–T4 pectoral muscle [cm^2/m^2]).									
7 [49]	observational studies	serious [aa,q,w]	serious [u]	serious [ab,b,y]	not serious	all possible residual confounding factors could reduce the observed effect	Meta-analysis: aHR 1.94 (1.30–2.90); p<0.001; I2=51%, N=828. Follow-up not specified. No data on nutritional assessment.	⊕⊕◯◯ LOW	CRITICAL
Mortality in ovarian cancer based on low SMM (evaluated with: L3 SMI [cm^2/m^2]).									
6 [50,51,52]	observational studies	not serious	serious [ac]	serious [ad]	serious [ae]	all possible residual confounding factors could reduce the observed effect	50. Meta-analysis: aHR 1.11 (1.03–1.20); p=0.02; I2=14%; p=0.31; N=456 (3 studies).<br><br>51. Meta-analysis: 3-year aOR 1.69 (0.82–3.47); p=0.15; I2=59%; p=0.08; N=467 (3 studies); 5-year aOR 1.76 (0.96–3.21); p=0.07; I2=56%; p=0.08; N=790 (4 studies).<br><br>52. Meta-analysis: aHR 1.10 (0.84–1.43); p=0.49; I2=51%; p=0.07; N=1226 (6 studies).	⊕⊕◯◯ LOW	CRITICAL

No. of studies	Certainty assessment						Impact	Certainty	Importance
	Study design	Risk of bias	Inconsistency	Indirectness	Imprecision	Other considerations			
Chemotherapy toxicity in patients with cytoreductive surgery and hyperthermic intraperitoneal chemotherapy based on low SMM (evaluated with: L3 SMI [cm^2/m^2]).									
1 [3]	observational studies	serious [af]	not serious	very serious [af,ag]	serious [f]	all possible residual confounding factors could reduce the observed effect	Chemotherapy toxicity: 57% with low SMM vs 26% without low SMM; p=0.004. aOR 3.97 (1.52–10.39); p=0.005; N=97 colorectal cancer. Follow-up 30 days. No differences when renal toxicity and neutropenia were analyzed independently.	⨁◯◯◯ VERY LOW	IMPORTANT
Chemotherapy toxicity in patients with colorectal cancer based on low SMM (evaluated with: L3 SMI [cm^2/m^2]).									
1 [24]	observational studies	serious [ah,j]	not serious	very serious [ag,ai]	serious [aj]	all possible residual confounding factors could reduce the observed effect	(Treatment delay, dose reduction or discontinuation): aOR 1.01 (0.35–2.91); p=0.99; N=67 advanced cancer before treatment with chemotherapy. Total follow-up: 3.5 years.	⨁◯◯◯ VERY LOW	IMPORTANT
Chemotherapy toxicity in patients with pancreatic cancer based on low SMM (evaluated with: L3 SMI [cm^2/m^2]).									
1 [26]	observational studies	serious [c]	not serious	very serious [ag]	serious [n]	all possible residual confounding factors could reduce the observed effect	No association with hematologic toxicity (p=0.060), non-hematologic toxicity (p=0.362) or intolerance (p=0.866); N=82 FOLFIRINOX. No mean follow-up data.	⨁◯◯◯ VERY LOW	IMPORTANT
Toxicity with sorafenib in patients with hepatocellular carcinoma based on low SMM (evaluated with: L3 SMI [cm^2/m^2]).									
3 [40,41,42]	observational studies	very serious [af,i,l]	serious [ak]	serious [al]	serious [n]	strong association. All possible residual confounding factors could reduce the observed effect	40. Only lack of association between SMM and toxicity is mentioned (no numerical data); N=278. Median follow-up: 54.9 months.<br>41. Treatment discontinuation due to adverse events: aHR 3.396 (1.731–6.664); p<0.001; N=82 treatment with sorafenib. Follow-up not specified.<br>42. Toxicity 62% with low SMM vs 40%; p=0.04; N=96 treatment with sorafenib. Follow-up not specified.	⨁◯◯◯ VERY LOW	IMPORTANT
Chemotherapy toxicity in patients with gastric cancer based on low SMM (evaluated with: L3 SMI [cm^2/m^2]).									

No. of studies	Certainty assessment						Impact	Certainty	Importance
	Study design	Risk of bias	Inconsistency	Indirectness	Imprecision	Other considerations			
1 [53]	observational studies	not serious	not serious	very serious [aj,b]	very serious [am]	all possible residual confounding factors could reduce the observed effect	Dose-limiting toxicity with low SMM: 64% vs 39%; p=0.180, N=48. Follow-up: 17 months. 10% of the sample with BMI <20 kg/m^2.	⊕◯◯◯ VERY LOW	IMPORTANT
Dose-limiting toxicity in patients with metastatic renal cancer based on low SMM (evaluated with: L3 SMI [cm^2/m^2]).									
4 [48]	observational studies	serious [a,z]	not serious	serious [b]	serious [c]	all possible residual confounding factors could reduce the observed effect	Meta-analysis (metastatic cancer on treatment with sunitinib or sorafenib): risk difference 16%; (2–31); p=0.03; I2=26%, N=242. Follow-up: not specified. Only in some studies BMI is taken into account.	⊕⊕◯◯ LOW	IMPORTANT
Post-surgical complications in patients with cytoreductive surgery and hyperthermic intraperitoneal chemotherapy based on low SMM (evaluated with: L3 SMI [cm^2/m^2]).									
4 [1,2,3,4]	observational studies	serious [c]	serious [e]	not serious	not serious	all possible residual confounding factors could reduce the observed effect	1. Major post-surgical complications: 56.2% with low SMM vs 52.9% without low SMM (p=0.723); N=115, pseudomyxoma (N=82) and peritoneal mesothelioma (N=33).<br><br>2. Post-surgical complications 42% with low SMM; no post-surgical complications 42.6% with low SMM (p=0.907); N=214 colon cancer.<br><br>3. Severe post-surgical complications: 51% with low SMM vs 44% without low SMM (p=0.464). OR 1.36 (0.60–3.10); p=0.465 (univariate); N=97 colorectal cancer.<br><br>4. SMM was lower in patients with severe complications: 43.3 v 47.0 cm^2/m^2 (p=0.005). L3 SMI was inversely correlated with the risk of severe postsurgical complications: aOR 0.93 (0.87–0.99); p=0.018; N=206 colorectal cancer.	⊕⊕⊕◯ MODERATE	IMPORTANT
Post-surgical complications in patients with colorectal cancer based on low SMM (evaluated with: L3 SMI–TPI [cm^2/m^2]).									

No. of studies	Certainty assessment						Impact	Certainty	Importance
	Study design	Risk of bias	Inconsistency	Indirectness	Imprecision	Other considerations			
7 [22,54,55]	observational studies	serious [an,l,q,w]	serious [ao,ap]	serious [b]	serious [f]	publication bias is strongly suspected. All possible residual confounding factors could reduce the demonstrated effect [aq]	22. Anastomotic leak OR 0.57 (0.28–1.19); p=0.13. Sepsis OR 1.49 (0.50–4.39); p=0.47; N=310 intestinal resection. Post-surgical follow-up.<br>54. Infection: aOR 4.6 (1.5–13.9); p=0.007. Need for rehabilitation: aOR 3.1(1.04–9.4); p=0.043; N=111 >64 years (no clear association in young people); intestinal resection. Follow-up: hospitalization.<br>55. Meta-analysis: Major complications RR 1.51; (0.63–3.63); I2=62%, N=1231. Post-surgical follow-up. Only 3 studies adjusted for other variables. No data on nutritional assessment.	⊕◯◯◯ VERY LOW	IMPORTANT
Post-surgical complications in patients with hepatocellular or pancreatic cancer surgery based on low SMM (evaluated with: L3 SMA, psoas muscle area normalized by height^2 or body area^2)									
25 [56]	observational studies	serious [aa,q]	not serious	serious [b,y]	not serious	all possible residual confounding factors could reduce the observed effect	Meta-analysis: RR associated with low total L3 SMM 1.36 (1.14–1.63); p=0.0008; I2=24%, N=3,501; 18 studies, median follow-up not specified. RR associated with low L3 SMM (psoas) 1.35 (1.15–1.58); p=0.0002; I2=0%, N=2,285; 7 studies, median follow-up not specified. No data on nutritional assessment.	⊕⊕⊕◯ MODERATE	IMPORTANT
Post-surgical complications in patients with gastric cancer based on low SMM (evaluated with: L3 SMA, psoas muscle area normalized by height^2 or body area^2)									
15 [43,44]	observational studies	serious [d,q,r]	serious [ar]	serious [b,s]	not serious	publication bias is strongly suspected. All possible residual confounding factors could reduce the demonstrated effect. [t]	43. Meta-analysis (12 studies): OR 2.09 (1.55–2.83); I2 53%, N=2100. OR severe complications 1.73 (1.14–2.63); I2=49%, N=1614. Follow-up: 30–64 months. Only 1 study adjusted for BMI.<br>44. Meta-analysis (8 studies): OR 1.76 (1.17–2.66); I2 77%, N=2913. OR severe complications 1.54 (1.03–2.29); I2=49%, N=2912. Follow-up: 1–60	⊕◯◯◯ VERY LOW	IMPORTANT

No. of studies	Certainty assessment						Impact	Certainty	Importance
	Study design	Risk of bias	Inconsistency	Indirectness	Imprecision	Other considerations			
							months. No data on nutritional assessment.		
Post-surgical complications in patients with esophageal cancer based on low SMM (evaluated with: L3 SMI [cm^2/m^2]).									
4 [45,55]	observational studies	serious [d,q]	not serious	serious [as,b]	not serious	publication bias is strongly suspected. All possible residual confounding factors could reduce the observed effect [aq]	45. Meta-analysis: OR 1.19 (0.78–1.81); p=0.431; I2=12.9%, N=571.<br><br>55. Meta-analysis: RR 1.15 (0.89–1.48); I2=0%, N=575. Follow-up: post-surgical. No data on nutritional assessment.	⨁⨁◯◯ LOW	IMPORTANT
Postoperative complications in patients with lung cancer based on low SMM (evaluated with: L3 SMI, T5 SMA, T12 SMI, L3 TPI).									
4 [46]	observational studies	serious [q]	not serious	serious [b,y]	not serious	all possible residual confounding factors could reduce the observed effect	Meta-analysis: OR 2.51 (1.55–4.08); I2=15%, N=836. Follow-up: perioperative. No data on nutritional assessment.	⨁⨁⨁◯ MODERATE	IMPORTANT
Post-surgical complications after total nephrectomy for renal cancer based on low SMM (evaluated with: L3 SMI [cm^2/m^2]).									
1 [57]	observational studies	very serious [at,au]	not serious	serious [ag]	very serious [c]	all possible residual confounding factors could reduce the observed effect	OR major complications: 4.2, (1.18–14.8); p=0.03; no association with minor complications; N=128. Median follow-up: 48.3 months.	⨁◯◯◯ VERY LOW	IMPORTANT
Mean hospital stay in patients with cytoreductive surgery and hyperthermic intraperitoneal chemotherapy based on low SMM (evaluated with: L3 SMI [cm^2/m^2]).									
1 [4]	observational studies	very serious [c]	not serious	serious [ag]	not serious	all possible residual confounding factors could reduce the observed effect	Mean hospital stay (days): 11.0 (8–19.5) with low SMM vs 11.5 (8–16) without low SMM (p=0.646); N=206 colorectal cancer.	⨁⨁◯◯ LOW	IMPORTANT
Mean hospital stay in patients with colorectal cancer surgery based on low SMM (evaluated with: L3 SMI [cm^2/m^2]).									
1 [54]	observational studies	very serious [l]	not serious	serious [ag]	not serious	all possible residual confounding factors could reduce the observed effect	12.3 with low SMM vs 15.9 days; p=0.038; N=234 intestinal resection.	⨁⨁◯◯ LOW	IMPORTANT
Mean hospital stay in patients with pancreatic cancer surgery based on low SMM (evaluated with: L3 SMI [cm^2/m^2]).									
1 [27]	observational studies	very serious [c]	not serious	not serious	serious [aj]	all possible residual confounding factors could	15.6 with low SMM vs 17.2 days; p=0.303; N=180 curative surgery.	⨁⨁◯◯ LOW	IMPORTANT

No. of studies	Certainty assessment						Impact	Certainty	Importance
	Study design	Risk of bias	Inconsistency	Indirectness	Imprecision	Other considerations			
						reduce the observed effect			
Mean hospital stay in patients with hepatocellular cancer based on low SMM (evaluated with: L3 SMI [cm^2/m^2]).									
1 [33]	observational studies	very serious [c]	not serious	not serious	serious [aj]	all possible residual confounding factors could reduce the observed effect	15 with low SMM vs 18 days; p=0.2330; SMM evaluated before curative surgery (N=139), ≥70 years.	⨁⨁◯◯ LOW	IMPORTANT
Mean hospital stay in patients with gastric cancer based on low SMM (evaluated with: L3 SMI–SMA/body area).									
8 [44]	observational studies	serious [q]	not serious	serious [b,y]	not serious	all possible residual confounding factors could reduce the observed effect	Meta-analysis: Mean difference 1.19; 95% CI 0.68–1.71; p<0.00001; I2=0%, N=3317. Follow-up: hospital stay. No data on nutritional status.	⨁⨁⨁◯ MODERATE	IMPORTANT
Cancer mortality based on myosteatosis (assessed with: L3 SMA/Psoas muscle area, skeletal muscle density [SMD] [Hounsfield units, HU], high intramuscular adipose tissue content [IMAC], value of paravertebral muscle attenuation, subcutaneous adipose tissue attenuation (4 studies) [HU])									
40 [58]	observational studies	serious [av,q]	serious [ap]	not serious	not serious	publication bias is strongly suspected. Strong association. All possible residual confounding factors could reduce the observed effect [aw]	Meta-analysis: HR 1.73 (1.58–1.90); p<0.00001; I2=62%, substantial heterogeneity; N=21,222. Follow-up: not specified for patients overall. Breast cancer: HR 1.30 (0.86–1.96); p=0.21, 2 studies. Gynecological cancer: HR 1.93 (1.28–2.91); p=0.002, I2=15%, 4 studies. Renal cancer: HR 1.83 (1.34–2.51); p=0.0002, I2=0%, 3 studies. Pancreatic cancer/periampullary cancer: HR 1.93 (1.60–2.33); p<0.0001; I2=39%, 8 studies**. Hepatocellular carcinoma: HR 1.88 (1.34–2.51); p<0.0001; I2=62%. 3 studies. Gastroesophageal cancer: HR 1.58 (1.16–2.15); p=0.004; I2=60%, 6 studies**. Colorectal cancer: HR 1.70 (1.49–1.94); p<0.00001; I2=56%, 11 studies**. Lymphoma: HR 3.65 (2.33–5.72); p<0.0001, I2=0%, 2 studies. **Classified by authors with high evidence.	⨁⨁⨁◯ MODERATE	CRITICAL
Mortality in breast cancer patients based on myosteatosis (assessed with: L3 IMAC index [cm^2/m^2]), SMD UH])									

No. of studies	Certainty assessment						Impact	Certainty	Importance
	Study design	Risk of bias	Inconsistency	Indirectness	Imprecision	Other considerations			
3 [5,6,8]	observational studies	serious [d]	serious [g]	not serious	serious [f]	all possible residual confounding factors could reduce the observed effect	5. HR 3.6 (1.2–10.8); p=0.02; N=119 non-metastatic breast cancer with aggressive features before chemotherapy. Median follow-up: 52.4 months.<br><br>6. HR 0.95 (0.78–1.16); N=3241 nonmetastatic/stage II-III. Median follow-up: 6 years.<br><br>8. aHR 2.04 (1.34–3.12); p=0.001; N=166 metastatic; 1 line of palliative chemotherapy. Median follow-up: 22 months.	⨁⨁◯◯ LOW	CRITICAL
Mortality in patients with colorectal cancer based on myosteatosis (assessed with: L3 SMD [UH])									
6 [11,12,13,14,18,24]	observational studies	serious [i,j]	not serious	not serious	serious [n]	all possible residual confounding factors could reduce the observed effect	11. aHR 1.54 (1.19–1.98); p=0.001; N=968 stage I-III colorectal cancer with preoperative CT. Median follow-up: 5.2 years.<br><br>12. aHR 1.80 (1.24–2.61); p=0.002; N=217 advanced colorectal cancer refractory to chemotherapy; ECOG 0–1. Median follow-up not specified.<br><br>13. aHR 1.42 (0.98–2.05); p=0;061; N=650 nonmetastatic colorectal cancer before surgery, no palliative treatment; regardless of mortality within 30 days post-surgery. Median follow-up not specified.<br><br>14. Not associated with survival (data not shown). The combination of low SMM and/or low visceral fat with high CRP was associated with higher mortality. N=97 before surgical resection of hepatic metastases of colorectal cancer. Mean follow-up: 73.6 months.<br><br>18. aHR 1.14 (0.67–1.93); p=0.622; N=805 colorectal cancer with elective	⨁⨁⨁◯ MODERATE	CRITICAL

No. of studies	Certainty assessment						Impact	Certainty	Importance
	Study design	Risk of bias	Inconsistency	Indirectness	Imprecision	Other considerations			
							surgical resection. Median follow-up: 47 months.<br><br>24. aHR 2.38 (1.16–4.87; p=0.018); N=67 advanced cancer before treatment with chemotherapy. Median survival: 17.5 months.		
Mortality in patients with pancreatic cancer based on myosteatosis (assessed with: L3 SMD [UH])									
5 [27,29,59,60,61]	observational studies	very serious [i,l,o]	not serious	not serious	serious [n]	all possible residual confounding factors could reduce the observed effect	27. Survival log rank test p=0.817; N=180 surgery/curative. Median follow-up not specified.<br><br>29. aHR 1.36 (0.92–2.00); p=0.120; N=228 unresectable. Median follow-up not specified.<br><br>59. Survival: 10.8 months with low muscle attenuation vs 15.9 months (p=0.046); N=47 periampullary cancer before surgery; objective was to compare with MRI. Median follow-up: 59.8 months.<br><br>60. aHR 1.57 (1.08–2.29); p=0.020; N=199 before. surgery. Median follow-up: 57.7 months.<br><br>61. Survival log rank test p=0.158. aHR 2.26 (1.08–4.64); p=0.030; N=83 resectable with neoadjuvant chemoradiotherapy. Median follow-up: 37.9 months.	⊕⊕◯◯ LOW	CRITICAL
Mortality in patients with hepatocellular cancer based on myosteatosis (assessed with: L3 SMD [UH])									
2 [34,40]	observational studies	very serious [j,l]	not serious	not serious	not serious	all possible residual confounding factors could reduce the observed effect	34. aHR 1.34 (1.05–1.71); p=0.001; N=1257 different stages. Follow-up not specified.<br><br>40. HR 0.97 (0.75–1.24); p=0.782; N=278 treatment with sorafenib. Median follow-up: 54.9 months.	⊕⊕⊕◯ MODERATE	CRITICAL
Mortality in patients with renal cancer as a function of the existence of myosteatosis (assessed with: L3 MDS (UH))									

No. of studies	Certainty assessment						Impact	Certainty	Importance
	Study design	Risk of bias	Inconsistency	Indirectness	Imprecision	Other considerations			
4 [48]	observational studies	serious [a,ax,z]	not serious	serious [b,y]	serious [d]	all possible residual confounding factors could reduce the observed effect	Meta-analysis: HR 1.56 (1.20–2.03); p=0.0008; I2=0%; N=429 metastatic cancer. Follow-up: 17–30.8 months. No nutritional assessment data.	⨁⨁◯◯ LOW	CRITICAL
Mortality in ovarian cancer based on myosteatosis (assessed with: L3 SMD [UH])									
4 [50,51,52]	observational studies	not serious	serious [u]	serious [ad]	not serious	all possible residual confounding factors could reduce the observed effect	50. Meta-analysis: aHR 1.13 (1.06–1.20); p=0.001; I2=62%; p=0.07; N=759 (3 studies).<br>51. Meta-analysis (with the same studies) aOR death at 3 years: 3.0 (2.02–4.45); p<0.001; I2=0%; p=0.65; N=620 (3 studies); aOR mortality at 5 years: 2.28 (1.55–3.36); p<0.001; I2=0%; p=0.71; N=620 (3 studies).<br>52. Meta-analysis: aHR 1.63 (1.28–2.07); p<0.001; I2=0%; p=0.44; N=679 (3 studies).	⨁⨁⨁◯ MODERATE	CRITICAL
Chemotherapy toxicity in patients with colorectal cancer based on myosteatosis (assessed with: L3 SMD [UH])									
1 [24]	observational studies	serious [ah,j]	not serious	very serious [ag,ai]	not serious	all possible residual confounding factors could reduce the observed effect	Treatment delay, dose reduction or discontinuation: aOR 1.43 (0.44–4.63; p=0.555); N=67 advanced cancer before treatment with chemotherapy. Total follow-up: 3.5 years.	⨁⨁◯◯ LOW	IMPORTANT
Chemotherapy toxicity in pancreatic cancer patients based on myosteatosis (assessed with: L3 SMD [UH])									
1 [61]	observational studies	very serious [c]	not serious	serious [b]	serious [ay]	all possible residual confounding factors could reduce the observed effect	Hematotoxicity: 60% vs 56%; p=0.928; N=83 resectable with neoadjuvant chemoradiotherapy. Follow-up: neoadjuvant period.	⨁◯◯◯ VERY LOW	IMPORTANT
Post-surgical complications in patients with hepatocellular carcinoma or pancreatic cancer surgery based on myosteatosis (assessed with: L3 SMD [UH], SMD, attenuation of intramuscular adipose tissue [IMAC, UH]).									
9 [56]	observational studies	serious [aa,ax]	not serious	serious [az,b]	not serious	all possible residual confounding factors could reduce the observed effect	Meta-analysis: RR associated with low muscle attenuation 1.40 (1.14–1.73); p=0.002; I2=4% N=885; 5 studies. Mean follow-up not	⨁⨁⨁◯ MODERATE	IMPORTANT

No. of studies	Certainty assessment						Impact	Certainty	Importance
	Study design	Risk of bias	Inconsistency	Indirectness	Imprecision	Other considerations			
							specified. RR associated with high IMAC 1.63 (1.28–2.09); p<0.0001; I2=0%, N=1,053; 4 studies. Mean follow-up not specified. No data on nutritional assessment.		
Median length of stay in patients with pancreatic cancer based on myosteatosis (assessed with: L3 SMD [UH])									
1 [61]	observational studies	very serious [c]	not serious	not serious	serious [ay]	all possible residual confounding factors could reduce the observed effect	(lowest quintile of muscle attenuation post-chemoradiotherapy): 42 vs 23 days; p=0.001; N=83 resectable cancer with neoadjuvant chemoradiotherapy. Follow-up: hospital stay.	⨁⨁◯◯ LOW	IMPORTANT
Mortality in patients with colorectal cancer based on SMM change during progression (evaluated with: SMI change [cm^2/m^2]/SMA change [cm^2]).									
3 [23,24,25]	observational studies	serious [ba,j]	serious [bb]	serious [b]	not serious	all possible residual confounders could reduce the observed dose-response gradient effect	23. (per lowest SD) aHR during disease progression and treatment 1.03 (0.90–1.18); 0.91 (0.78–1.04); 1.02 (0.88–1.15); 1.19 (1.09–1.35); 1.14 (0.99–1.30); 1.54 (1.31–1.79); N=450 systemic palliative treatment. Median follow-up not specified.<br><br>24. (>8% loss in 3 months): aHR 4.47 (2.21–9.05; p=0.001); N=63 advanced cancer on treatment with chemotherapy. Median survival: 17.5 months.<br><br>25. (SMM loss 5%): aHR 2.079 (1.194–3.619; p=0.010); N=148 advanced cancer before and after chemotherapy. Median follow-up: 23.2 months.	⨁⨁⨁◯ MODERATE	CRITICAL
Mortality in patients with biliopancreatic cancer based on SMM change during progression (evaluated with: SMI change [cm^2/m^2]/SMA change [cm^2]).									
2 [27,62]	observational studies	serious [bc,j]	not serious	serious [b]	not serious	all possible residual confounding factors could reduce the observed effect	27. (percent change in SMM/60 days): aHR 0.94 (0.92–0.96; p=0.001); N=180 before and after surgery. Median follow-up: 62 days.<br><br>62. (decrease vs. maintenance of SMM): aHR 1.390 (1.109–1.742;	⨁⨁⨁◯ MODERATE	CRITICAL

No. of studies	Certainty assessment						Impact	Certainty	Importance
	Study design	Risk of bias	Inconsistency	Indirectness	Imprecision	Other considerations			
							p=0.004); N=484 palliative chemotherapy. Median follow-up: 11 months.		
Mortality in patients with renal cancer based on SMM change during progression (evaluated with: SMI change [cm^2/m^2]).									
2 [63,64]	observational studies	serious [bd]	not serious	serious [ag]	not serious	all possible residual confounding factors could reduce the observed effect	63. aHR mortality change SMM (loss ≥ 5%): 2.367 (1.253–4.469); p=0.008; N=101 metastatic renal cancer. Median follow-up: 30.8 months.<br><br>64. aHR mortality change SMM (increase ≥ 5%): 0.02 (0.00–0.13); p<0.001; N=37 renal cancer after nephrectomy. Median follow-up: 61 months.	⨁⨁⨁◯ MODERATE	CRITICAL
Chemotherapy toxicity in patients with colorectal cancer based on SMM change during progression (evaluated with: SMA change [cm^2]).									
1 [24]	observational studies	serious [ah]	not serious	serious [ai]	not serious	all possible residual confounding factors could reduce the observed effect	(Treatment delay, dose reduction or discontinuation per 1% decrease in SMM): aOR 1.03 (0.95–1.12; p=0.441); N=63 advanced cancer on treatment with chemotherapy. Total follow-up: 3.5 years.	⨁⨁⨁◯ MODERATE	IMPORTANT
Need for surgery in patients with inflammatory bowel disease (IBD) based on low SMM (evaluated with: L3 SMI [cm^2/m^2]).									
3 [65,66,67]	observational studies	serious [be]	serious [ac]	serious [bf]	very serious [bg,bh]	all possible residual confounding factors could reduce the observed effect	65. Higher probability of surgery in patients with low SMM (2 SD below the norm for younger healthy adults): Kaplan-Meier curves; log-rank 0.003; n=99 ulcerative colitis. Median follow-up: 5 months.<br><br>66. aHR need for surgery high vs low SMM: 0.318 (0.126–0.802); p=0.015; log-rank test only significant for Crohn's disease; N=72 (Crohn's disease and ulcerative colitis requiring admission). Median follow-up not specified.<br><br>67. Low SMM: aOR colectomy 90 days 1.55 (0.31–7.71); aOR colectomy 1 year 1.54 (0.37–6.32); N=89 ulcerative colitis with	⨁◯◯◯ VERY LOW	IMPORTANT

No. of studies	Certainty assessment						Impact	Certainty	Importance
	Study design	Risk of bias	Inconsistency	Indirectness	Imprecision	Other considerations			
							intravenous steroids. Follow-up: 1 year.		
Post-surgical complications in patients with IBD based on low SMM (evaluated with: L3 SMI/TPI [cm^2/m^2]).									
2 [68,69]	observational studies	serious [be]	serious [bi,bj]	serious [bf]	not serious	all possible residual confounding factors could reduce the observed effect	68. Low SMM (SMI) (2 SD below the norm for younger healthy adults): aOR major complications, 1.11 (1.02–1.22; p=0.023); no difference in minor complications; N=114 Crohn's disease. Follow-up: postoperative; not specified.<br><br>69. Low SMM (TPI) in patients <40 years (lowest quartile by sex): aOR transfusion 1.49 (1.08–2.07); p=0.0159; no association patients ≥40 years nor for major complications, ICU admission, postoperative sepsis or deep vein thrombosis (DVT); N=178 in the complete sample (Crohn's disease and ulcerative colitis). Post-surgical follow-up.	⊕⊕◯◯ LOW	IMPORTANT
Need for rescue treatment during hospitalization (medical or surgical) in IBD patients based on low SMM (evaluated with: L3 SMI [cm^2/m^2]).									
1 [67]	observational studies	serious [bk]	not serious	serious [bl]	not serious	all possible residual confounding factors could reduce the observed effect	Low SMM: aOR 3.98 (1.12–14.1); p=0;033; N=89 ulcerative colitis with intravenous steroids. Follow-up: hospitalization.	⊕⊕⊕◯ MODERATE	IMPORTANT
Hospital stay in patients with IBD based on low SMM (evaluated with: L3 SMI [cm^2/m^2]).									
1 [68]	observational studies	serious [be]	not serious	serious [bm]	not serious	all possible residual confounding factors could reduce the observed effect	Low SMM (SMI) (2 SD below the norm for younger healthy adults): 10.86 ± 8.3 vs. 11.41 ± 10.61 days; p=0.552; N=114 Crohn's disease. Follow-up: hospitalization after surgery.	⊕⊕⊕◯ MODERATE	IMPORTANT
Post-surgical complications in patients with IBD based on low SMM (evaluated with: L3 SMD [UH]).									
1 [69]	observational studies	serious [be]	serious [bj]	serious [bf]	not serious	all possible residual confounding factors could	Low SMM density (L3) in patients <40 years (lowest quartile by sex): aOR transfusion 1.31 (1.056–1.625); p=0.014; aOR ICU admission 1.32 (1.053–	⊕⊕◯◯ LOW	IMPORTANT

No. of studies	Certainty assessment						Impact	Certainty	Importance
	Study design	Risk of bias	Inconsistency	Indirectness	Imprecision	Other considerations			
						reduce the observed effect	1.656); p=0.016; aOR sepsis 1.325 (1.072–1.636); p=0.0091; aOR DVT 1.265 (1.043–1.535); p=0.0173; aOR major complications: 1.329 (1.056–1.671); p=0.0052; no association in patients ≥40; N=178 in the complete sample (Crohn's disease and ulcerative colitis). Post-surgical follow-up.		
Short-term mortality (<30 days) in patients undergoing transcatheter aortic valve implantation (TAVI) based on low SMM (assessed with: L3 SMI-Psoas area/body surface area-Psoas area/BMI).									
5 [70]	observational studies	serious [bn,q]	not serious	serious [b]	serious [bo]	all possible residual confounding factors could reduce the observed effect	Meta-analysis: OR (high vs low SMM) 0.72; 95% CI 0.44–1.18; p=0.285; I2=20.36%, N=896.	⨁⨁◯◯ LOW	CRITICAL
Mid- to long-term mortality (>30 days) in patients undergoing TAVI based on low SMM (follow-up: 6–24 months; assessed with: L3 SMI-Psoas area/body surface area-Total psoas area).									
6 [70]	observational studies	serious [a,bn,q,w]	serious [u]	serious [b]	serious [bo]	all possible residual confounding factors could reduce the observed effect	Meta-analysis: OR (high vs low SMM) 0.49; 95% CI 0.28–0.83; p=0.049; I2=54.96%, N=1453. Follow-up: 6–24 months.	⨁◯◯◯ VERY LOW	CRITICAL
Mid-term major cardiovascular events in patients with predialysis advanced chronic kidney disease (ACKD) based on SMM (assessed with: L3 TPI [cm^2/m^2])									
1 [71]	observational studies	very serious [bp,bq]	not serious	serious [ag]	serious [br]	all possible residual confounding factors could reduce the observed effect	aHR MACE (low vs high SMM) 3.98 (1.65–9.63); P=0.0022; N=266. Median follow-up: 3.2 years.	⨁◯◯◯ VERY LOW	IMPORTANT
Mortality in patients with critical limb ischemia based on SMM (assessed with: L3 SMA [cm^2]/psoas area index [cm^2]/L4 vertebral body area [cm^2]).									
2 [72,73]	observational studies	very serious [at,bs]	serious [ac]	serious [bt]	very serious [bu,d]	all possible residual confounding factors could reduce the observed effect	72. aHR death (low vs. high SMM [lower quintile of healthy population]): 3.2 (1.24–9.11); p=0.02; N=64. Median follow-up: 3.5 years.<br>73. aHR mortality with SMM increase: 10.9 (1.7–72); P=0.013; N=188. Median follow-up: 1 year.	⨁◯◯◯ VERY LOW	CRITICAL
Cardiovascular events in patients with limb ischemia/peripheral vascular disease based on low SMM (assessed with: L3 SMA [cm^2]/Total psoas area [cm^2]).									

No. of studies	Certainty assessment						Impact	Certainty	Importance
	Study design	Risk of bias	Inconsistency	Indirectness	Imprecision	Other considerations			
2 [74,75]	observational studies	very serious [at,bv,l]	serious [ac]	serious [bw]	not serious	all possible residual confounding factors could reduce the observed effect	74. aHR new cardiovascular event (low vs high SMM [lower quintile of healthy population]): 3.07 (1.56–6.29); p<0.01; N=114 critical limb ischemia. Follow-up: 3 years.<br>75. HR new CV event (per 1 SD increase in SMM): 0.754 (0.568–1.007); p=0.057; N=327 peripheral vascular disease. Median follow-up: 909 days.	⊕◯◯◯ VERY LOW	IMPORTANT
Cardiovascular events in patients with PVD based on low SMM (assessed with: L3 SMM psoas muscle [UH])									
1 [75]	observational studies	serious [at]	not serious	serious [ag]	not serious	all possible residual confounding factors could reduce the observed effect	aHR CV event (per 1 SD increase in SMM density [UH]); two models: 0.784 (0.617–0.955); p=0.045 and 0.699 (0.548–0.889); p=0.003; N=327 PVD. Median follow-up: 909 days.	⊕⊕⊕◯ MODERATE	IMPORTANT
Major amputation in patients with critical ischemia and low SMM (evaluated with: Psoas area index [cm^2]/L4 vertebral body area [cm^2]).									
1 [73]	observational studies	very serious [at,bs]	not serious	serious [ag]	serious [bx]	all possible residual confounding factors could reduce the observed effect	aHR major amputation based on SMM: 2.6 (0.83–8.3); p=0.099; N=188. Median follow-up: 1 year.	⊕◯◯◯ VERY LOW	IMPORTANT
Mortality in critically ill patients based on low SMM (evaluated with: L3 SMI [cm^2/m^2]/L3 SMA [cm^2]/T7-T8 Pectoralis muscle area [cm^2]/L3 Total psoas area [cm^2]).									
7 [76,77,78,79,80,81,82]	observational studies	very serious [at,by]	not serious	not serious	not serious	all possible residual confounding factors could reduce the observed effect	76. Mortality aOR (per unit increase in SMI): 0.93 (0.875–0.997); p=0.025, adjusted for BMI and albumin; N=149 elderly with traumatic injuries. Follow-up: hospitalization.<br>77. Mortality aOR per 10 cm^2 increase in SMM: 0.82 (0.73–0.93); p=0.001; mortality aOR: 3.86 (1.80–8.26); p=0.001; N=240 mechanically ventilated critically ill adults. Follow-up: hospitalization.<br>78. 30-day mortality aOR after extubation due to SMI increase: 0.94 (0.890-0.995); p=0.033; N=231 critically ill adults after	⊕⊕⊕◯ MODERATE	CRITICAL

No. of studies	Certainty assessment						Impact	Certainty	Importance
	Study design	Risk of bias	Inconsistency	Indirectness	Imprecision	Other considerations			
							mechanical ventilation. Follow-up: hospitalization.<br><br>79. 30-day survival aHR with low SMM: 2.74 (1.02–7.35); N=99 critically ill oncology patients. Follow-up: 30 days.<br><br>80. Mortality aHR with low SMM: 2.1 (1.1–4.0), p=0.018; N=236 critically ill patients with intra-abdominal sepsis. Follow-up: 30 days.<br><br>81. 6-month mortality aOR per cm^2 increase in SMM: 0.98 (0.968–0.999); p=0.007; N=401 critically ill patients in the medical ICU.<br><br>82. ICU mortality aOR with increased psoas muscle area: 0.812 (0.741–0.890); p<0.001; N=362. Follow-up: hospitalization.		
Ventilator-free days in critically ill patients based on low SMM (assessed with: L3 SMI [cm^2/m^2])									
1 [76]	observational studies	very serious [at,bz]	very serious [ac]	serious [ag]	serious [ca]	all possible residual confounding factors could reduce the observed effect	Low vs high SMM: Median 19 days (IQR 0–28) vs 27 (IQR 18–28); p=0.004. Correlation coefficient ventilator-free days per SMI increase: 0.060 (-0.025, 0.126); p=0.19, adjusted for BMI and albumin N=149 elderly with traumatic injuries.	⨁◯◯◯ VERY LOW	IMPORTANT
ICU-free days in critically ill patients based on low SMM (assessed with: L3 SMI [cm^2/m^2])									
1 [76]	observational studies	very serious [at,bz]	very serious [ac]	serious [ag]	serious [ca]	all possible residual confounding factors could reduce the observed effect	Low vs high SMM: median 19 days (IQR 0–25) vs 16 (IQR 0–24); p=0.002). Correlation coefficient ventilator-free days per SMI increase: 0.032 (-0.037, 0.101); p=0.36 adjusted for BMI and albumin; N=149 elderly with traumatic injuries.	⨁◯◯◯ VERY LOW	IMPORTANT
Mean length of stay in critically ill patients based on low SMM (assessed with: L3 SMI [cm^2/m^2])									
1 [78]	observational studies	serious [at]	not serious	serious [ag]	not serious	all possible residual confounding	aIRR of mean stay after extubation per SMI increase: 0.99 (0.986-	⨁⨁⨁◯ MODERATE	IMPORTANT

No. of studies	Certainty assessment						Impact	Certainty	Importance
	Study design	Risk of bias	Inconsistency	Indirectness	Imprecision	Other considerations			
						factors could reduce the observed effect	1.000); p=0.048; aIRR total mean hospital stay per SMI increase 0.99 (0.987–1.000); p=0.053; aIRR total mean ICU stay per SMI increase 0.99 (0.986–1.000); p=0.065; N=231 critically ill adults after mechanical ventilation. Follow-up: hospitalization.		
Mortality in critically ill patients based on myosteatosis (assessed with: L3 SMD [HU] and IMAC [cm^2])									
1 [83]	observational studies	serious [at]	not serious	serious [ag]	not serious	all possible residual confounding factors could reduce the observed effect	6-month mortality aHR per 10 HU (SMD): 0.774 (0.643–0.931); p=0.006; 6-month mortality aHR per 10 cm^2 (IMAC): 1.092 (0.966–1.236; p=0.159; N=491 critically ill adults on mechanical ventilation. Follow-up 6 months.	⨁⨁⨁◯ MODERATE	CRITICAL
Mean length of stay in critically ill patients based on fat infiltration of SMM (assessed with: L3 SMD [UH] and IMAC [cm^2])									
1 [83]	observational studies	serious [at]	not serious	serious [ag]	not serious	all possible residual confounding factors could reduce the observed effect	Correlation coefficient mean hospital stay per 10 HU (SMD): -0.134 (-0.228 to -0.040); p=0.005. Correlation coefficient mean hospital stay per 10 cm^2 (IMAC): 0.064 (-0.012 to 0.141); p=0.100; Correlation coefficient mean ICU stay per 10 HU (SMD): -0.032 (-0.128 to 0.063) p=0.506; Correlation coefficient mean ICU stay per 10 cm^2 (IMAC): 0.041 (-0.036 to 0.119); p=0.292; N=491. Follow-up: 6 months.	⨁⨁⨁◯ MODERATE	IMPORTANT
Overall mortality in liver cirrhosis based on low SMI (assessed with: L3 SMI [cm^2/m^2]/total psoas area [cm^2]/ TPI [cm^2/m^2])									
15 [84,85]	observational studies	very serious [at,d,q]	serious [ar]	serious [cb]	not serious	publication bias is strongly suspected. All possible residual confounding factors could reduce the demonstrated effect [cc]	84. Meta-analysis: mortality aHR with low SMM 2.11 (1.50–2.95); I2=89%, p<0.001; N=2781 (no transplantation; regardless of waiting list). Follow-up not clearly defined.<br><br>85. Mortality aHR with low SMM 1.72 (1.27–2.32); p<0.001; I2=75.5%; N=1247 (transplanted and non-transplanted).	⨁◯◯◯ VERY LOW	CRITICAL

No. of studies	Certainty assessment						Impact	Certainty	Importance
	Study design	Risk of bias	Inconsistency	Indirectness	Imprecision	Other considerations			
Mortality on liver transplant waiting for patients with cirrhosis based on low SMI (evaluated with: L3 SMI [cm^2/m^2]/SMA [cm^2]/ psoas muscle area [cm^2]/TPI [cm^2/m^2]).									
10 [84,86]	observational studies	very serious [at,d,q]	serious [cd]	serious [cb]	serious [g]	publication bias is strongly suspected. All possible residual confounding factors could reduce the demonstrated effect. [ce]	84. Meta-analysis: mortality aHR death with low SMM 1.72 (0.99–3.00); p=0.05; I2=33%; p=0.22; N=583. Follow-up: up to 5 years in some studies (time used for HR not specified).<br>86. Mortality aHR with low SMM 1.86 (1.23–2.84); I2 not defined; N=1969. Follow-up not clearly specified.	⨁◯◯◯ VERY LOW	CRITICAL
Post-liver transplant mortality in patients with cirrhosis based on low SMI (evaluated with: L3 and L3-L4 SMI space [cm^2/m^2]/total psoas area [cm^2]).									
7 [86]	observational studies	very serious [at,d,q]	serious [cf]	not serious	not serious	all possible residual confounding factors could reduce the observed effect	Meta-analysis: mortality aHR with low SMM 1.84 (1.11–3.05); p=0.02; I2=60%; p=0.06; N=748. Global follow-up not specified. Mortality aHR per SMI increase 0.98 (0.96–1.00); p=0.03; I2=13%; p=0.32; N=835. Overall follow-up not specified.	⨁⨁◯◯ LOW	CRITICAL
Sepsis or severe infection after liver transplantation in patients with cirrhosis based on low SMM (evaluated with: L3 SMI [cm^2/m^2] and total psoas muscle area [cm^2]).									
2 [85]	observational studies	very serious [at,d]	serious [u]	serious [cg]	serious [br]	all possible residual confounding factors could reduce the observed effect	Meta-analysis: aHR sepsis/infection with low SMM 2.81 (1.15–6.87); p<0.05; I2=45%; N=452. Post-surgery follow-up.	⨁◯◯◯ VERY LOW	IMPORTANT

aHR, adjusted hazard ratio; aIRR, adjusted incidence rate ratio, IQR, interquartile range; HU, Hounsfield units; IMAC, high intramuscular adipose tissue content; SMD, skeletal muscle density; SMI, skeletal muscle index; SMM, skeletal muscle mass.

Explanations

a. Short follow-up time for the event of interest in some studies.

b. Evidence cannot be generalized to all patients with disease-related malnutrition. No data on nutritional status in the sample.

c. No adjustment for confounding factors

d. Poor adjustment for confounding factors; confounding factors not well defined in some studies.

e. The studies show different associations based on low SMM, mainly due to the statistical methods used and the method used to assess low SMM.

f. Wide confidence interval in multivariate analysis in some studies.

g. Different estimates of the effect

h. Patients with low weight were excluded from the analysis.

i. In some studies, the median follow-up is not defined.

j. Not all the studies explain how mortality was determined.

k. The variables used for statistical adjustment were different between studies.

l. In some studies, there was no adjustment for confounding factors.

m. Variability in point estimates (Park, 2018).

n. Small samples size in some studies.

o. Inappropriate eligibility criteria in some studies.

p. CT performed at different disease stages in some studies.

q. Non-standardized cut-off value or method for defining low muscle mass.

r. Some statistical errors were detected in the analysis of individual studies.

s. Most of the studies involved patients of Asian origin. Patients with carcinomas in situ or undergoing palliative treatment were excluded in one of the meta-analyses. Generalizability of results cannot be guaranteed.

t. Some degree of publication bias is observed in small studies (funnel-plot) in the first meta-analysis.

u. Moderate heterogeneity.

v. Cut-off points mainly from Canadian population. Several Asian articles. Generalizability of results cannot be guaranteed.

w. Many retrospective studies.

x. Substantial heterogeneity.

y. Most of the studies include Asian populations. Generalizability of results cannot be guaranteed.

z. Most of the studies included in the meta-analysis were classified as moderate quality.

aa. The meta-analysis leaves several points unspecified (also in supplementary material): mean follow-up, publication bias detected.

ab. Data cannot be generalized to all types of hematologic tumors.

ac. Different estimates for the event of interest.

ad. Case series of only 6 studies.

ae. In more than 50% of the studies, the null value is included in the confidence interval.

af. Toxicity not well defined.

ag. Single-center. Questionable generalizability of results.

ah. It is not clear if an adjusted OR is used.

ai. Toxicity criteria are not well defined.

aj. Small sample size.

ak. Difficult to assess differences in point estimates due to lack of numerical data in some studies.

al. Adverse events are mentioned, without clear toxicity criteria.

am. No adjusted OR is given for this event.

an. No adjusted data are shown for younger patients in one of the studies.

ao. Different events in studies with different measurement methods.

ap. Substantial heterogeneity.

aq. Small study effect in the complete meta-analysis.

ar. Substantial heterogeneity.

as. Similar cut-off values for different populations. Generalizability of results may not be guaranteed.

at. Comparability of the groups cannot be guaranteed.

au. Retrospective evaluation of the event.

av. Follow-up time is not specified.

aw. Funnel plot with high risk of publication bias is shown.

ax. Cut-off points to define low muscle attenuation or high intramuscular adipose tissue content, not standardized.

ay. 95% CI not shown.

az. All studies using IMAC have been conducted in Asian population. Generalizability of results cannot be guaranteed.

ba. Eligibility criteria to guarantee external validity, given it is a sub-analysis of a clinical trial (one of the studies).

bb. Differences related to different sample sizes and statistical adjustments.

bc. Some studies perform the second CT scan at different times during disease progression.

bd. Comparability of the groups in one of the studies cannot be guaranteed.

be. Patients with and without sarcopenia were not comparable in all previous interventions (no further statistical adjustment).

bf. Only patients with CT scan were included in the study (does not represent the overall IBD population).

bg. Few events of interest (surgery).

bh. Some studies do not provide numerical data on surgery-free time, only Kaplan-Meier curves.

bi. Contradictory results for major complications.

bj. Disparate data for <40 and >40 years of age.

bk. Limited statistical adjustment.

bl. Only refers to ulcerative colitis, not Crohn's disease.

bm. Only refers to Crohn's disease, not ulcerative colitis.

bn. Some data (quality of the studies, risk of bias) are not specified in the meta-analysis.

bo. Statistical adjustment data are not included (variables needed for adjustment were not considered in the meta-analysis).

bp. The groups were not comparable in age or BMI.

bq. Evaluation of the event is not well explained.

br. Wide confidence interval.

bs. Short follow-up time for the event of interest.

bt. Case series in only two centers, with heterogeneous comorbidity; generalization of results cannot be guaranteed.

bu. Wide confidence interval in one of the studies.

bv. Only one study defines events by hospitalization.

bw. Asian population only; generalizability of results cannot be guaranteed.

bx. Statistical adjustment data are not included.

by. No statistical adjustment by age and, in some studies, age is different between patients with and without sarcopenia.

bz. No adjustment for confounding factors in the estimate related to diagnosis of low SMM.

ca. Wide interquartile range in the unadjusted estimate.

cb. Several studies refer to data from the same center.

cc. Risk of publication bias in non-Asian population.

cd. Heterogeneity not defined in all meta-analyses.

ce. Not all events were described in all studies.

cf. Substantial heterogeneity in one of the meta-analyses.

cg. Only two studies included

References

1. Galan A, Rousset P,Mercier F,Képénékian V,Valette PJ,Glehen O,Passot G. Overall survival of pseudomyxoma peritonei and peritoneal mesothelioma patients after cytoreductive surgery and hyperthermic intraperitoneal chemotherapy can be predicted by computed tomography quantified sarcopenia. Eur J Surg Oncol. ; 2018.

2. Banaste N, Rousset P,Mercier F,Rieussec C,Valette PJ,Glehen O,Passot G. Preoperative nutritional risk assessment in patients undergoing cytoreductive surgery plus hyperthermic intraperitoneal chemotherapy for colorectal carcinomatosis. Int J Hyperthermia; 2018.

3. Chemama S, Bayar MA,Lanoy E,Ammari S,Stoclin A,Goéré D,Elias D,Raynard B,Antoun S. Sarcopenia is Associated with Chemotherapy Toxicity in Patients Undergoing Cytoreductive Surgery with Hyperthermic Intraperitoneal Chemotherapy for Peritoneal Carcinomatosis from Colorectal Cancer. Ann Surg Oncol.; 2016.

4. van Vugt JL, Braam HJ,van Oudheusden TR,Vestering A,Bollen TL,Wiezer MJ,de Hingh IH,van Ramshorst B,Boerma D. Skeletal Muscle Depletion is Associated with Severe Postoperative Complications in Patients Undergoing Cytoreductive Surgery with Hyperthermic Intraperitoneal Chemotherapy for Peritoneal Carcinomatosis of Colorectal Cancer. Ann Surg Oncol; 2015.

5. Deluche E, Leobon S,Desport JC,Venat-Bouvet L,Usseglio J,Tubiana-Mathieu N. Impact of body composition on outcome in patients with early breast cancer. Support Care Cancer; 2018.

6. Caan BJ, Cespedes Feliciano EM,Prado CM,Alexeeff S,Kroenke CH,Bradshaw P, Quesenberry CP,Weltzien EK,Castillo AL,Olobatuyi TA,Chen WY. Association of Muscle and Adiposity Measured by Computed Tomography With Survival in Patients With Nonmetastatic Breast Cancer. JAMA Oncol.; 2018.

7. Del Fabbro E, Parsons H,Warneke CL,Pulivarthi K,Litton JK,Dev R,Palla SL,Brewster A,Bruera E. The relationship between body composition and response to neoadjuvant chemotherapy in women with operable breast cancer. Oncologist.; 2012.

8. Rier HN, Jager A,Sleijfer S,van Rosmalen J,Kock MCJM,Levin MD. Low muscle attenuation is a prognostic factor for survival in metastatic breast cancer patients treated with first line palliative chemotherapy. Breast. ; 2017.

9. Fattouh M, Chang GY,Ow TJ,Shifteh K,Rosenblatt G,Patel VM,Smith RV,Prystowsky MB,Schlecht NF. Association between pretreatment obesity, sarcopenia, and survival in patients with head and neck cancer. Head Neck; 2019.

10. Chargi N, Bril SI,Emmelot-Vonk MH,de Bree R. Sarcopenia is a prognostic factor for overall survival in elderly patients with head-and-neck cancer. Eur Arch Otorhinolaryngol.; 2019.

11. Hopkins JJ, Reif RL,Bigam DL,Baracos VE,Eurich DT,Sawyer MB. The Impact of Muscle and Adipose Tissue on Long-term Survival in Patients With Stage I to III Colorectal Cancer. Dis Colon Rectum; 2019.

12. Charette N, Vandeputte C,Ameye L,Bogaert CV,Krygier J,Guiot T,Deleporte A,Delaunoit T,Geboes K,Van Laethem JL,Peeters M,Demolin G,Holbrechts S,Flamen P,Paesmans M,Hendlisz A. Prognostic value of adipose tissue and muscle mass in advanced colorectal cancer: a post hoc analysis of two non-randomized phase II trials. BMC Cancer; 2019.

13. Dolan RD, Almasaudi AS,Dieu LB,Horgan PG,McSorley ST,McMillan DC. The relationship between computed tomography-derived body composition, systemic inflammatory response, and survival in patients undergoing surgery for colorectal cancer. J Cachexia Sarcopenia Muscle; 2019.

14. van Dijk DPJ, Krill M,Farshidfar F,Li T,Rensen SS,Olde Damink SWM,Dixon E,Sutherland FR,Ball CG,Mazurak VC,Baracos VE,Bathe OF. Host phenotype is associated with reduced survival independent of tumour biology in patients with colorectal liver metastases. J Cachexia Sarcopenia Muscle; 2019.

15. Park SE, Hwang IG,Choi CH,Kang H,Kim BG,Park BK,Cha SJ,Jang JS,Choi JH. Sarcopenia is poor prognostic factor in older patients with locally advanced rectal cancer who received preoperative or postoperative chemoradiotherapy. Medicine (Baltimore); 2018.

16. Caan BJ, Meyerhardt JA,Kroenke CH,Alexeeff S,Xiao J,Weltzien E,Feliciano EC,Castillo AL,Quesenberry CP,Kwan ML,Prado CM. Explaining the Obesity Paradox: The Association between Body Composition and Colorectal Cancer Survival (C-SCANS Study). Cancer Epidemiol Biomarkers Prev.; 2017.

17. Feliciano EMC, Kroenke CH,Meyerhardt JA,Prado CM,Bradshaw PT,Kwan ML,Xiao J,Alexeeff S,Corley D,Weltzien E,Castillo AL,Caan BJ. Association of Systemic Inflammation and sarcopenia with survival in nonmetastatic colorectal cancer. JAMA Oncol.; 2017.

18. Malietzis G, Currie AC,Athanasiou T,Johns N,Anyamene N,Glynne-Jones R,Kennedy RH,Fearon KC,Jenkins JT. Influence of body composition profile on outcomes following colorectal cancer surgery. Br J Surg.; 2016.

19. Miyamoto Y, Baba Y,Sakamoto Y,Ohuchi M,Tokunaga R,Kurashige J,Hiyoshi Y, Iwagami S,Yoshida N,Yoshida M,Watanabe M,Baba H. Sarcopenia is a Negative Prognostic Factor After Curative Resection of Colorectal Cancer. Ann Surg Oncol.; 2015.

20. Thoresen L, Frykholm G,Lydersen S,Ulveland H,Baracos V,Prado CM,Birdsell L,Falkmer U. Nutritional status, cachexia and survival in patients with advanced colorectal carcinoma. Different assessment criteria for nutritional status provide unequal results. Clin Nutr.; 2013.

21. van Vledder MG, Levolger S,Ayez N,Verhoef C,Tran TC,Ijzermans JN. Body composition and outcome in patients undergoing resection of colorectal liver metastases. Br J Surg; 2012.

22. Reisinger KW, van Vugt JL,Tegels JJ,Snijders C,Hulsewé KW,Hoofwijk AG,Stoot JH,Von Meyenfeldt MF,Beets GL,Derikx JP,Poeze M. Functional compromise reflected by sarcopenia, frailty, and nutritional depletion predicts adverse postoperative outcome after colorectal cancer surgery. Ann Surg.; 2015.

23. Kurk SA, Peeters PHM,Dorresteijn B,de Jong PA,Jourdan M,Creemers GM,Erdkamp FLG,de Jongh FE,Kint PAM,Poppema BJ,Radema SA,Simkens LHJ,Tanis BC,Tjin-A-Ton MLR,Van Der Velden A,Punt CJA,Koopman M,May AM. Loss of skeletal muscle index and survival in patients with metastatic colorectal cancer: Secondary analysis of the phase 3 CAIRO3 trial. Cancer Med.; 2020.

24. Blauwhoff-Buskermolen S, Versteeg KS,de van der Schueren MA,den Braver NR,Berkhof J,Langius JA,Verheul HM. Loss of Muscle Mass During Chemotherapy Is Predictive for Poor Survival of Patients With Metastatic Colorectal Cancer. J Clin Oncol.; 2016.

25. Miyamoto Y, Baba Y,Sakamoto Y,Ohuchi M,Tokunaga R,Kurashige J,Hiyoshi Y, Iwagami S,Yoshida N,Watanabe M,Baba H. Negative Impact of Skeletal Muscle Loss after Systemic Chemotherapy in Patients with Unresectable Colorectal Cancer. PLoS One.; 2015.

26. Kurita Y, Kobayashi N,Tokuhisa M,Goto A,Kubota K,Endo I,Nakajima A,Ichikawa Y. Sarcopenia is a reliable prognostic factor in patients with advanced pancreatic cancer receiving FOLFIRINOX chemotherapy. Pancreatology.; 2019.

27. Choi MH, Yoon SB,Lee K,Song M,Lee IS,Lee MA,Hong TH,Choi MG. Preoperative sarcopenia and post-operative accelerated muscle loss negatively impact survival after resection of pancreatic cancer. J Cachexia Sarcopenia Muscle.; 2018.

28. El Amrani M, Vermersch M,Fulbert M,Prodeau M,Lecolle K,Hebbar M,Ernst O, Pruvot FR,Truant S. Impact of sarcopenia on outcomes of patients undergoing pancreatectomy: A retrospective analysis of 107 patients. Medicine (Baltimore).; 2018.

29. van Dijk DP, Bakens MJ,Coolsen MM,Rensen SS,van Dam RM,Bours MJ,Weijenberg MP,Dejong CH,Olde Damink SW. Low skeletal muscle radiation attenuation and visceral adiposity are associated with overall survival and surgical site infections in patients with pancreatic cancer. J Cachexia Sarcopenia Muscle.; 2017.

30. Mintziras I, Miligkos M,Wächter S,Manoharan J,Maurer E,Bartsch DK. Sarcopenia and sarcopenic obesity are significantly associated with poorer overall survival in patients with pancreatic cancer: Systematic review and meta-analysis. Int J Surg. ; 2018.

31. Takada H, Kurosaki M,Nakanishi H,Takahashi Y,Itakura J,Tsuchiya K,Yasui Y,Tamaki N,Takaura K,Komiyama Y,Higuchi M,Kubota Y,Wang W,Okada M,Enomoto N,Izumi N. Impact of pre-sarcopenia in sorafenib treatment for advanced hepatocellular carcinoma. PLoS One; 2018.

32. Begini P, Gigante E,Antonelli G,Carbonetti F,Iannicelli E,Anania G,Imperatrice B,Pellicelli AM,Fave GD,Marignani M. Sarcopenia predicts reduced survival in patients with hepatocellular carcinoma at first diagnosis. Ann Hepatol.; 2017.

33. Harimoto N, Yoshizumi T,Shimokawa M,Sakata K,Kimura K,Itoh S,Ikegami T,Ikeda T,Shirabe K,Maehara Y. Sarcopenia is a poor prognostic factor following hepatic resection in patients aged 70 years and older with hepatocellular carcinoma. Hepatol Res.; 2016.

34. Fujiwara N, Nakagawa H,Kudo Y,Tateishi R,Taguri M,Watadani T,Nakagomi R, Kondo M,Nakatsuka T,Minami T,Sato M,Uchino K,Enooku K,Kondo Y,Asaoka Y,Tanaka Y,Ohtomo K,Shiina S,Koike K. Sarcopenia, intramuscular fat deposition, and visceral adiposity independently predict the outcomes of hepatocellular carcinoma. J Hepatol.; 2015.

35. Voron T, Tselikas L,Pietrasz D,Pigneur F,Laurent A,Compagnon P,Salloum C,Luciani A,Azoulay D. Sarcopenia Impacts on Short- and Long-term Results of Hepatectomy for Hepatocellular Carcinoma. Ann Surg.; 2015.

36. Kamachi S, Mizuta T,Otsuka T,Nakashita S,Ide Y,Miyoshi A,Kitahara K,Eguchi Y,Ozaki I,Anzai K. Sarcopenia is a risk factor for the recurrence of hepatocellular carcinoma after curative treatment. Hepatol Res. ; 2016.

37. Levolger S, van Vledder MG,Muslem R,Koek M,Niessen WJ,de Man RA,de Bruin RW,Ijzermans JN. Sarcopenia impairs survival in patients with potentially curable hepatocellular carcinoma. J Surg Oncol.; 2015.

38. Itoh S, Shirabe K,Matsumoto Y,Yoshiya S,Muto J,Harimoto N,Yamashita Y,Ikegami T,Yoshizumi T,Nishie A,Maehara Y. Effect of body composition on outcomes after hepatic resection for hepatocellular carcinoma. Ann Surg Oncol.; 2014.

39. Harimoto N, Shirabe K,Yamashita YI,Ikegami T,Yoshizumi T,Soejima Y,Ikeda T,Maehara Y,Nishie A,Yamanaka T. Sarcopenia as a predictor of prognosis in patients following hepatectomy for hepatocellular carcinoma. Br J Surg.; 2013.

40. Labeur TA, van Vugt JLA,Ten Cate DWG,Takkenberg RB,IJzermans JNM,Groot Koerkamp B,de Man RA,van Delden OM,Eskens FALM,Klümpen HJ. Body Composition Is an Independent Predictor of Outcome in Patients with Hepatocellular Carcinoma Treated with Sorafenib. Liver Cancer.; 2019.

41. Sawada K, Saitho Y,Hayashi H,Hasebe T,Nakajima S,Ikuta K,Fujiya M,Okumura T. Skeletal muscle mass is associated with toxicity, treatment tolerability, and additional or subsequent therapies in patients with hepatocellular carcinoma receiving sorafenib treatment. JGH Open.; 2019.

42. Antonelli G, Gigante E,Iavarone M,Begini P,Sangiovanni A,Iannicelli E,Biondetti P,Pellicelli AM,Miglioresi L,Marchetti P,Lampertico P,Marignani M. Sarcopenia is associated with reduced survival in patients with advanced hepatocellular carcinoma undergoing sorafenib treatment. United European Gastroenterol J.; 2018.

43. Borggreve AS, den Boer RB,van Boxel GI,et al. The Predictive Value of Low Muscle Mass as Measured on CT Scans for Postoperative Complications and Mortality in Gastric Cancer Patients: A Systematic Review and Meta-Analysis. Clin Med. ; 2020.

44. Rinninella E, Cintoni M,Raoul P,et al. Muscle mass, assessed at diagnosis by L3-CT scan as a prognostic marker of clinical outcomes in patients with gastric cancer: A systematic review and meta-analysis. Clin Nutr. ; 2020.

45. Boshier PR, Heneghan R,Markar SR,Baracos VE,Low DE. Assessment of body composition and sarcopenia in patients with esophageal cancer: a systematic review and meta-analysis. Dis Esophagus. ; 2018.

46. Nishimura JM, Ansari AZ,D'Souza DM,Moffatt-Bruce SD,Merritt RE,Kneuertz PJ. Computed Tomography-Assessed Skeletal Muscle Mass as a Predictor of Outcomes in Lung Cancer Surgery. Ann Thorac Surg; 2019.

47. Deng HY, Hou L,Zha P,Huang KL,Peng L. Sarcopenia is an independent unfavorable prognostic factor of non-small cell lung cancer after surgical resection: a comprehensive systematic review and meta-analysis. Eur J Surg Oncol. ; 2019.

48. Vrieling A, Kampman E,Knijnenburg NC,et al. Body Composition in Relation to Clinical Outcomes in Renal Cell Cancer: A Systematic Review and Meta-analysis. Eur Urol Focus. ; 2018.

49. Surov A, Wienke A. Sarcopenia predicts overall survival in patients with malignant hematological diseases: A meta-analysis . Clin Nutr. ; 2020.

50. Ubachs J, Ziemons J,Minis-Rutten IJG,Kruitwagen RFPM,Kleijnen J,Lambrechts S,Olde Damink SWM,Rensen SS,Van Gorp T. Sarcopenia and ovarian cancer survival: a systematic review and meta-analysis. J Cachexia Sarcopenia Muscle; 2019.

51. McSharry V, Mullee A,McCann L,Rogers AC,McKiernan M,Brennan DJ. The Impact of Sarcopenia and Low Muscle Attenuation on Overall Survival in Epithelial Ovarian Cancer: A Systematic Review and Meta-analysis. Ann Surg Oncol; 2020.

52. Rinninella E, Fagotti A,Cintoni M,Raoul P,Scaletta G,Scambia G,Gasbarrini A,Mele MC. Skeletal muscle mass as a prognostic indicator of outcomes in ovarian cancer: a systematic review and meta-analysis. Gynecol Cancer; 2020.

53. Palmela C, Velho S,Agostinho L,Branco F,Santos M,Santos MPC,et al. Body composition as a prognostic factor of neoadjuvant chemotherapy toxicity and outcome in patients with locally advanced gastric cancer. Journal of Gastric Cancer; 2017.

54. Lieffers JR, Bathe OF,Fassbender K,Winget M,Baracos VE. Sarcopenia is associated with postoperative infection and delayed recovery from colorectal cancer resection surgery. Br J Cancer.; 2012.

55. Simonsen C, de Heer P,Bjerre ED,et al. Sarcopenia and Postoperative Complication Risk in Gastrointestinal Surgical Oncology: A Meta-analysis. Ann Surg. ; 2018.

56. Cao Q, Xiong Y,Zhong Z,Ye Q. Computed Tomography-Assessed Sarcopenia Indexes Predict Major Complications following Surgery for Hepatopancreatobiliary Malignancy: A Meta-Analysis. Ann Nutr Metab. ; 2019.

57. Peyton CC, Heavner MG,Rague JT,Krane LS,Hemal AK. Does Sarcopenia Impact Complications and Overall Survival in Patients Undergoing Radical Nephrectomy for Stage III and IV Kidney Cancer. J Endourol; 2016.

58. Aleixo GFP, Shachar SS,Nyrop KA,Muss HB,Malpica L,Williams GR. Myosteatosis and prognosis in cancer: Systematic review and meta-analysis. Crit Rev Oncol Hematol. ; 2020.

59. Rollins KE, Tewari N,Ackner A,Awwad A,Madhusudan S,Macdonald IA,Fearon KC,Lobo DN. The impact of sarcopenia and myosteatosis on outcomes of unresectable pancreatic cancer or distal cholangiocarcinoma. Clin Nutr.; 2016.

60. van Dijk DPJ, Bakers FCH,Sanduleanu S,Vaes RDW,Rensen SS,Dejong CHC,Beets-Tan RGH,Olde Damink SWM. Myosteatosis predicts survival after surgery for periampullary cancer: a novel method using MRI. HPB (Oxford).; 2018.

61. Akahori T, Sho M,Kinoshita S,Nagai M,Nishiwada S,Tanaka T,Tamamoto T,Ohbayashi C,Hasegawa M,Kichikawa K,Nakajima Y. Prognostic Significance of Muscle Attenuation in Pancreatic Cancer Patients Treated with Neoadjuvant Chemoradiotherapy. World J Surg.; 2015.

62. Choi Y, Oh DY,Kim TY,Lee KH,Han SW,Im SA,Kim TY,Bang YJ. Skeletal Muscle Depletion Predicts the Prognosis of Patients with Advanced Pancreatic Cancer Undergoing Palliative Chemotherapy, Independent of Body Mass Index. PLoS One.; 2015.

63. Gu W, Wu J,Liu X,Zhang H,Shi G,Zhu Y,Ye D. Early skeletal muscle loss during target therapy is a prognostic biomarker in metastatic renal cell carcinoma patients. Sci Rep ; 2017.

64. Fukushima H, Nakanishi Y,Kataoka M,Tobisu KI,Koga F. Postoperative Changes in Skeletal Muscle Mass Predict Survival of Patients With Metastatic Renal Cell Carcinoma Undergoing Cytoreductive Nephrectomy. Clin Genitourin Cancer; 2017.

65. Zhang T, Ding C,Xie T,Yang J,Dai X,Lv T,Li Y,Gu L,Wei Y,Gong J,Zhu W,Li N,Li J. Skeletal muscle depletion correlates with disease activity in ulcerative colitis and is reversed after colectomy. Clin Nutr.; 2017.

66. Bamba S, Sasaki M,Takaoka A,Takahashi K,Imaeda H,Nishida A,Inatomi O,Sugimoto M,Andoh A. Sarcopenia is a predictive factor for intestinal resection in admitted patients with Crohn's disease. PLoS One.; 2017.

67. Cushing KC, Kordbacheh H,Gee MS,Kambadakone A,Ananthakrishnan AN. Sarcopenia is a Novel Predictor of the Need for Rescue Therapy in Hospitalized Ulcerative Colitis Patients. J Crohns Colitis; 2018.

68. Zhang T, Cao L,Cao T,Yang J,Gong J,Zhu W,Li N,Li J. Prevalence of Sarcopenia and Its Impact on Postoperative Outcome in Patients With Crohn's Disease Undergoing Bowel Resection. JPEN J Parenter Enteral Nutr. ; 2017.

69. Pedersen M, Cromwell J,Nau P. Sarcopenia is a Predictor of Surgical Morbidity in Inflammatory Bowel Disease. Inflamm Bowel Dis.; 2017.

70. Soud M, Alahdab F,Ho G,et al. Usefulness of skeletal muscle area detected by computed tomography to predict mortality in patients undergoing transcatheter aortic valve replacement: a meta-analysis study. Int J Cardiovasc Imaging. ; 2019.

71. Harada K, Suzuki S,Ishii H,Aoki T,Hirayama K,Shibata Y,Negishi Y,Sumi T,Kawashima K,Kunimura A,Shimbo Y,Tatami Y,Kawamiya T,Yamamoto D,Morimoto R,Yasuda Y,Murohara. Impact of Skeletal Muscle Mass on Long-Term Adverse Cardiovascular Outcomes in Patients With Chronic Kidney Disease. Am J Cardiol; 2017.

72. Matsubara Y, Matsumoto T,Aoyagi Y,Tanaka S,Okadome J,Morisaki K,Shirabe K,Maehara Y. Sarcopenia is a prognostic factor for overall survival in patients with critical limb ischemia. J Vasc Surg; 2015.

73. Nyers ES, Brothers TE. Perioperative psoas to lumbar vertebral index does not successfully predict amputation-free survival after lower extremity revascularization. J Vasc Surg; 2017.

74. Matsubara Y, Matsumoto T,Inoue K,Matsuda D,Yoshiga R,Yoshiya K,Furuyama T,Maehara Y. Sarcopenia is a risk factor for cardiovascular events experienced by patients with critical limb ischemia. J Vasc Surg; 2017.

75. Sugai T, Watanabe T,Otaki Y,Goto J,Watanabe K,Toshima T,Takahashi T,Yokoyama M,Tamura H,Nishiyama S,Arimoto T,Takahashi H,Shishido T,Watanabe M. Decreased Psoas Muscle Computed Tomography Value Predicts Poor Outcome in Peripheral Artery Disease. Circ J; 2018.

76. Moisey LL, Mourtzakis M,Cotton BA,Premji T,Heyland DK,Wade CE,Bulger E,Kozar RA, (NUTRIC), Nutrition,and,Rehabilitation,Investigators,Consortium. Skeletal muscle predicts ventilator-free days, ICU-free days, and mortality in elderly ICU patients. Crit Care; 2013.

77. Weijs PJ, Looijaard WG,Dekker IM,Stapel SN,Girbes AR,Oudemans-van Straaten HM,Beishuizen A. Low skeletal muscle area is a risk factor for mortality in mechanically ventilated critically ill patients. Crit Care. ; 2014.

78. Fuchs G, Thevathasan T,Chretien YR,Mario J,Piriyapatsom A,Schmidt U,Eikermann M,Fintelmann FJ. Lumbar skeletal muscle index derived from routine computed tomography exams predict adverse post-extubation outcomes in critically ill patients. J Crit Care; 2018.

79. Toledo DO, Carvalho AM,Oliveira AMRR,Toloi JM,Silva AC,Francisco de Mattos Farah J,Prado CM,Silva JM Jr. The use of computed tomography images as a prognostic marker in critically ill cancer patients. Clin Nutr ESPEN; 2018.

80. Ji Y, Cheng B,Xu Z,Ye H,Lu W,Luo X,Fu S,Fang X. Impact of sarcopenic obesity on 30-day mortality in critically ill patients with intra-abdominal sepsis. J Crit Care; 2018.

81. Jaitovich A, Khan MMHS,Itty R,Chieng HC,Dumas CL,Nadendla P,Fantauzzi JP,Yucel RM,Feustel PJ,Judson MA. ICU Admission Muscle and Fat Mass, Survival, and Disability at Discharge: A Prospective Cohort Study. Chest; 2019.

82. Toptas M, Yalcin M,Akkoc İ,Demir E,Metin C,Savas Y,Kalyoncuoglu M,Can MM. The Relation between Sarcopenia and Mortality in Patients at Intensive Care Unit. Biomed Res Int; 2018.

83. Looijaard WG, Dekker IM,Stapel SN,Girbes AR,Twisk JW,Oudemans-van Straaten HM,Weijs PJ. Skeletal muscle quality as assessed by CT-derived skeletal muscle density is associated with 6-month mortality in mechanically ventilated critically ill patients. Crit Care; 2016.

84. Chang KV, Chen JD,Wu WT,Huang KC,Han DS. Association of loss of muscle mass with mortality in liver cirrhosis without or before liver transplantation: A systematic review and meta-analysis. Medicine (Baltimore); 2019.

85. Kim G, Kang SH,Kim MY,Baik SK. Prognostic value of sarcopenia in patients with liver cirrhosis: A systematic review and meta-analysis. PLoS On; 2017.

86. van Vugt JL, Levolger S,de Bruin RW,van Rosmalen J,Metselaar HJ,IJzermans JN. Systematic Review and Meta-Analysis of the Impact of Computed Tomography-Assessed Skeletal Muscle Mass on Outcome in Patients Awaiting or Undergoing Liver Transplantation. Am J Transplant. ; 2016.

Topic 7: Functional status and health-related quality of life

Author: Alejandro Sanz-Paris

Question: Prognostic value of altered functional and quality of life tests in patients with disease-related malnutrition (or at risk of malnutrition) during short- and medium-term follow-up.

Setting: General population is not included. Chronic or acute illnesses are included.

No. of studies	Certainty assessment						Effect			Certainty	Importance
	Study design	Risk of bias	Inconsistency	Indirectness	Imprecision	Other considerations	No. of events	No. of individuals	Rate (95% CI)		
Overall long-term (808 days of follow-up) and short-term (in-hospital) mortality (hospitalized elderly) (assessed with: 6-minute walk test [6MWT])											
1[1]	observational study	not serious [a]	not serious	Not serious [b]	Serious [c]	all possible residual confounding factors could reduce the observed effect	41 (in-hospital deaths).<br><br>173 (total deaths).	310	6MWT inability to perform:<br><br>Short-term (logistic regression): OR: 3.26 (95% CI 1.38–7.69) (P<0.05)<br><br>Long term (Cox regression): HR: 2.15 (95% CI 1.35–3.42) P<0.05)	⊕⊕⊕◯ MODERATE	IMPORTANT
Long-term overall mortality (989 days of follow-up) (hospitalized elderly) (evaluated with: Short Physical Performance Battery [SPPB] test)											
1[2]	observational study	Not serious	not serious	Not serious [d]	Not serious	all possible residual confounding factors could reduce the observed effect	109	298	SPPB: 0<br><br>Cox regression: HR 1.81 (95% CI 1.20–2.73) P<0.005	⊕⊕⊕◯ MODERATE	IMPORTANT
Long-term mortality (24 months) (elderly in long-term care facilities) (evaluated with: Barthel Index)											
1[3]	observational study	Not serious	not serious	serious[e]	serious[f]	all possible residual confounding factors could reduce the observed effect	94	276	K-Barthel Index (score from 0 to 20)<br><br>(Univariate logistic regression): OR: 0.93 (95% CI 0.89–0.97)[g]	⊕⊕◯◯ LOW[h]	IMPORTANT
Long-term (12.8 months) overall mortality in oncology patients (elderly outpatient) (assessed with: ECOG and Karnofsky Performance Scale [KPS])											
1[4]	observational study	Not serious	Not serious	Not serious[i]	Not serious	all possible residual confounding factors could	152	455	Univariate regression.<br><br>ECOG: (1 unit increase). HR: 1.75 (95% CI	⊕⊕⊕◯ MODERATE[j]	IMPORTANT

No. of studies	Certainty assessment						Effect			Certainty	Importance
	Study design	Risk of bias	Inconsistency	Indirectness	Imprecision	Other considerations	No. of events	No. of individuals	Rate (95% CI)		
						reduce the observed effect			1.42–2.15) (P<0.01).<br>KPS: (10 unit increase). HR: 0.79 (95% CI 0.70–0.89) (P<0.01)<br>In 311 solid tumors, multivariate model. ECOG: (1 unit increase). HR: 2.05 (95% CI 1.56–2.71) (P<0.01)		
Long-term overall mortality at 12 months in oncologic patients (evaluated with: ECOG-PS, timed up and go test [TUG], activities of daily living [Katz index]).											
1[5]	observational study	Not serious	Not serious	Not serious[k]	Not serious	all possible residual confounding factors could reduce the observed effect	379	993	multivariate Cox regression.<br>ECOG 3–4 vs 0–1. HR: 3.33 (95% CI 2.42–4.58) (P<0.01).<br>Katz Index <5/6. HR: 1.73 (95% CI 1.31–3.00) (P<0.01).<br>TUG > 20 s. HR: 2.39 (95% CI 1.84–3.10) (P<0.01).	⊕⊕⊕◯ MODERATE	IMPORTANT
Long-term overall mortality at 12 months in oncology patients (advanced small cell lung cancer) (evaluated with: ECOG).											
1[6]	observational study	Not serious	Not serious	Not serious[l]	Serious[m]	all possible residual confounding factors could reduce the observed effect	(not indicated)	119	multivariate regression analysis. ECOG: 0–1 vs 2; RR: 1.75 (95% CI 1.42–2.15) (P<0.01).	⊕◯◯◯ VERY LOW	LIMITED IMPORTANCE
One-year overall mortality (clinical populations with hip fracture and at risk of malnutrition) (assessed by Barthel index)											
1[7]	observational study	Not serious	not serious	Serious[n,o]	very serious[p]	all possible residual confounding factors could reduce the observed effect	197	850	univariate analysis. Preoperative Barthel index OR: 0.89 (95% CI 0.86–0.92), P<0.001.<br>Multivariate analysis. Barthel index OR: 0.96 (95%	⊕◯◯◯ VERY LOW	IMPORTANT

No. of studies	Certainty assessment					Effect			Certainty	Importance
	Study design	Risk of bias	Inconsistency	Indirectness	Imprecision	Other considerations	No. of events	No. of individuals	Rate (95% CI)		
									CI 0.92–1.01) (P=0.091)[q]		
Mortality with a mean follow-up of 589 days (peritoneal dialysis patients) (evaluated with: 10-meter walk test).											
1[8]	observational study	not serious	not serious	Serious[r,s]	Serious[t]	none	7	119	10-meter walk test. HR: 19.3 (95% CI 0.82–454.1) (P=0.066).[u,v]	⊕◯◯◯ VERY LOW	IMPORTANT
Overall mortality at 24 months in patients with end-stage renal disease without replacement therapy (outpatient) (assessed with: Barthel index and SF-36).											
1[9]	observational study	Not serious[e]	not serious	Serious[w]	Serious[x]	all possible residual confounding factors could reduce the observed effect	38	82	univariate regression analysis.<br>Barthel index HR: 0.98 (95% CI 0.97–0.99) (p=0.005).<br>SF-36 (physical component) HR: 0.92 (95% CI 0.89–1.00) (P=0.05).<br>Statistical significance for both is lost in the multivariate analysis. The mental component of SF-36 had no statistical significance.	⊕◯◯◯ VERY LOW	IMPORTANT
5-year mortality in chronic hemodialysis patients (outpatient) (evaluated with: SF-36).											
1[10]	observational study	not serious	not serious	Serious[y]	not serious	all possible residual confounding factors could reduce the observed effect	127	420	multivariate regression analysis.<br>**SF-36** (physical component for each one-point decrease), RR: 1.02 (95% CI 1.01–1.03) (P=0.018).	⊕⊕◯◯ LOW	IMPORTANT
2-year mortality in chronic hemodialysis patients (outpatient) (evaluated with: Kidney Disease Quality of Life-Short Form (KDQOL-SF)).											

No. of studies	Certainty assessment						Effect			Certainty	Importance
	Study design	Risk of bias	Inconsistency	Indirectness	Imprecision	Other considerations	No. of events	No. of individuals	Rate (95% CI)		
1[11]	observational studies	Not serious	not serious	Serious[z]	Not serious	all possible residual confounding factors could reduce the observed effect	86	714	multivariate regression analysis.<br>KDQOL-SF. Physical functioning, HR: 1.72 (95% CI 1.02–2.73) (P=0.02).<br>Emotional health, HR: 1.85 (95% CI 1.30–2.63) (P=0.001).<br>Social functioning, HR: 1.59 (95% CI 1.12–2.26) (P=0.01).	⊕◯◯◯ VERY LOW	IMPORTANT
2-year mortality in patients with end-stage renal disease (EGFR=25.8 ml/min/1.73 m^2) (outpatient) (assessed with: EQ-5D index score [EQ-5D-QL], which ranges -0.594 to 1 [1=complete health and lower values indicate worse health-related quality of life]).											
1[12]	observational studies	Not serious	not serious	Very serious[aa]	Not serious	all possible residual confounding factors could reduce the observed effect	46	745	multivariate regression analysis.<br>EQ-5D-QL HR: 0.28 3 (95% CI 0.099–0.810) (P=0.019)	⊕⊕◯◯ LOW	IMPORTANT
Mortality at 2 years (heart failure) (evaluated with: Barthel index)											
1[13]	observational studies	Not serious	not serious	Serious[ab]	Serious[ac]	all possible residual confounding factors could reduce the observed effect	21	151	Multivariate Cox regression model. Barthel index with a logarithmic transformation, HR: 4.35 (95% CI 1.06–17.9) (P=0.004).	⊕⊕◯◯ LOW	IMPORTANT
12-month mortality (heart failure) (evaluated with: 6MWT at hospital discharge)											
1[14]	observational studies	Not serious	not serious	Serious[ae]	Not serious	all possible residual confounding factors could reduce the observed effect	36	430	Multivariate regression model.<br>6MWT (one unit decrease in meters walked) HR: 1.005 (95% CI 1.003–1.008) (P<0.001)	⊕⊕◯◯ LOW	IMPORTANT

No. of studies	Certainty assessment						Effect			Certainty	Importance
	Study design	Risk of bias	Inconsistency	Indirectness	Imprecision	Other considerations	No. of events	No. of individuals	Rate (95% CI)		
Long-term overall mortality (288 days) in patients with acute coronary syndrome (ACS) (hospitalized elderly) (evaluated with: SPPB).											
1[15]	observational study	Not serious	Not serious	not serious[ae]	Not serious[af]	all possible residual confounding factors could reduce the observed effect	94	908	Multiple regression model.<br>SPPB (for single change unit) HR: 0.88 (95% CI 0.82–0.5) (P=0.001)	⊕⊕⊕⊕ HIGH	IMPORTANT
Overall 5-year mortality in patients with cardiovascular disease (elderly outpatient) (evaluated with: one-leg standing time).											
1[16]	observational study	Not serious	Not serious	Serious[ag]	Not serious	all possible residual confounding factors could reduce the observed effect	165	908	Multivariate Cox regression model. Lowest tertile (<3.03 sec) HR: 1.68 (95% CI 1.06–2.67) (P=0.03), compared to highest tertile ≥ 9.71 sec.	⊕⊕⊕◯ MODERATE	IMPORTANT
Overall long-term mortality (688 days) after percutaneous endoscopic gastrostomy (elderly with neurological disease) (evaluated with: Karnofsky performance status [KPS])											
1[17]	observational study	Not serious	Not serious	It is serious[ah]	It is not serious	all possible residual confounding factors could reduce the observed effect	38	110	multivariate regression analysis.<br>KPS <60% OR: 9.78 (95% CI 3.26–29.3) (P<0.0001)	⊕⊕◯◯ LOW	IMPORTANT
12-month mortality in lung transplant patients (evaluated with: SPPB).											
1[18]	observational study	Not serious	Not serious	Serious[ai]	Not serious	all possible residual confounding factors could reduce the observed effect	46	259	Cox multiple regression model, adjusted for age, sex, race/ethnicity, diagnosis and BMI, and FEV_1.<br>SPPB at 12 months (1 point worsening), HR: 1.15 (95% CI 1.05–1.25).	⊕⊕⊕◯ MODERATE	CRITICAL
Overall 12-month mortality in cirrhotic patients (MELD >12) awaiting liver transplantation (evaluated with: SPPB).											

No. of studies	Certainty assessment						Effect			Certainty	Importance
	Study design	Risk of bias	Inconsistency	Indirectness	Imprecision	Other considerations	No. of events	No. of individuals	Rate (95% CI)		
1[19]	observational studies	Not serious	Not serious	Serious[aj,ak]	Not serious	all possible residual confounding factors could reduce the observed effect	36	294	Bivariate regression model<br><br>SPPB (per 1 point decrease). Adjusted by MELD and age, HR: 1.16 (95% CI 1.04–1.30) (P<0.01).<br><br>SPPB adjusted by MELD and albumin, HR: 1.18 (95% CI 1.06–1.31) (P<0.01).	⊕⊕⊕◯ MODERATE	IMPORTANT
Overall mortality at 24 months in cirrhotic patients awaiting liver transplantation (assessed with: Gait speed on the 6MWT [GS-6MWT]).											
1[20.]	observational study	Not serious	Not serious	Not serious[al,am]	serious[an]	all possible residual confounding factors could reduce the observed effect	12	73	Multivariate regression model.<br><br>GS <1m/s and 6MWT <400 m.<br><br>HR: 3.21 (95% CI 1.03–9.96) (P<0.044).	⊕⊕⊕◯ MODERATE	IMPORTANT
6-month mortality in HIV patients with and without associated tuberculosis (outpatients) (assessed with: KPS)											
1[21]	observational study	Not serious	not serious	Serious[ao]	Serious[ap]	all possible residual confounding factors could reduce the observed effect	37	812	Multivariate regression analysis.<br><br>KPS <80% aHR: 4.3 (95% CI 1.84–10.08), P<0.05.<br><br>In patients with tuberculosis (n=158) HR: 8.30 (95% CI 1.06–65.14) (P<0.05).	⊕⊕◯◯ LOW	IMPORTANT
In-hospital mortality (hospitalized elderly) (evaluated with: Katz index)											
1[22]	observational study	Not serious	Not serious	Serious[aq]	serious[ar]	all possible residual confounding factors could reduce the observed effect	15	105	Multiple logistic regression model. OR: 6.1 (1.4–26.3), P=0.015.	⊕⊕◯◯ LOW	IMPORTANT
In-hospital mortality in patients with heart failure (adults >20 years) (assessed with: Barthel index at hospital admission).											

No. of studies	Certainty assessment						Effect			Certainty	Importance
	Study design	Risk of bias	Inconsistency	Indirectness	Imprecision	Other considerations	No. of events	No. of individuals	Rate (95% CI)		
1[23]	observational study	not serious	not serious	Not serious[as]	not serious	strong association. All possible residual confounding factors could reduce the observed effect	101	11301	Multivariate logistic regression analysis model OR: 0.989 (95% CI 0.983–0.995), P<0.00.	⊕⊕⊕◯ MODERATE	IMPORTANT
In-hospital mortality in frail elderly patients with percutaneous coronary intervention (evaluated with: Barthel index).											
1[24.]	observational study	Not serious	Not serious	Serious[at]	Not serious	all possible residual confounding factors could reduce the observed effect	18	259	Regression model OR: 0.98 (95%CI 0.95–0.99), P=0.05.	⊕◯◯◯ VERY LOW	IMPORTANT
In-hospital mortality in critically ill patients over 65 years of age (evaluated with: activities of daily living with Katz index).											
1[25]	observational study	Not serious	Not serious	Serious[au]	Not serious	all possible residual confounding factors could reduce the observed effect	130	249	Katz index <6.<br><br>Adjusted logistic regression model OR: 2.47 (95% CI 1.21–5.06), P<0.01.	⊕⊕◯◯ LOW	IMPORTANT
In-hospital mortality (hospitalized clinical populations at risk of malnutrition) (evaluated with: SPPB)											
1[26]	observational study	Not serious	not serious	Serious[av,aw]	Serious[ax]	all possible residual confounding factors could reduce the demonstrated effect	221	1621	SPPB (per point decline) OR: 0.4359 [d] (P=0.0507	⊕◯◯◯ VERY LOW	IMPORTANT
3-month mortality (hospitalized elderly) (evaluated with: Katz index)											
1[27]	observational study	not serious	not serious	Serious[ay]	not serious	all possible residual confounding factors could reduce the observed effect	37	291	Univariate analysis, OR: 1.16 (95% CI 0.98–1.37), P=0.093; multivariate analysis, OR: 1.00 (95% CI 0.82–1.23) P=0.986.	⊕◯◯◯ VERY LOW.	LIMITED IMPORTANCE
3-month mortality in cancer patients (elderly outpatient) (evaluated with: ECOG and EORTC Quality of Life Questionnaire-Core 30 global health status scale).											
1[28]	observational studies	Not serious	Not serious	Not serious[az]	Not serious	all possible residual confounding factors could reduce the observed effect	1259	6769	multivariate regression analysis.<br><br>ECOG (2–4) OR: 2.25 (95% CI	⊕⊕⊕⊕ HIGH	CRITICAL

No. of studies	Certainty assessment						Effect			Certainty	Importance
	Study design	Risk of bias	Inconsistency	Indirectness	Imprecision	Other considerations	No. of events	No. of individuals	Rate (95% CI)		
									1.89–2.69), P<0.001. EORTC (based on a 10-point difference) scale OR: 0.98 (95% CI 0.98–0.99) p<0.001.		
Overall 30-day mortality after palliative procedure in advanced cancer patients (evaluated with: ECOG).											
1[29]	observational studies	Not serious	Not serious	Serious[aaa]	Not serious	all possible residual confounding factors could reduce the observed effect	92	823	multivariate regression analysis. ECOG: ≥2 HR: 2.2 (1.6–3), P<0.01.	⨁◯◯◯ VERY LOW	LIMITED IMPORTANCE
Complications after admission for stroke: discharge to home/residence (evaluated with: Barthel index)											
1[30]	observational studies	not serious	not serious	Serious[aab]	not serious	all possible residual confounding factors could reduce the observed effect	151	205	OR: 1.05 (95% CI 1.03–1.07), P<0.001.	⨁⨁◯◯ LOW	IMPORTANT
Complication: onset of dysphagia 2 months after hospital admission (hospitalized elderly) (assessed with: Barthel index).											
1[31]	observational study	Not serious	not serious	Serious[aac]	Serious[aad]	all possible residual confounding factors could reduce the observed effect	21	95	OR 12.9 (95% CI 2.1–78.4), P=0.005. Barthel index cut-off value: 50/55	⨁◯◯◯ VERY LOW	LIMITED IMPORTANCE
Complications of bladder post-void residual volume in patients with hip fracture (elderly hospitalized) (assessed with: TUG)											
1[32]	observational studies	not serious	not serious	Serious[aae]	not serious	all possible residual confounding factors could reduce the observed effect	44	345	Age-adjusted linear regression model, OR: 1.02 (95% CI 1.004-1.03), P<0.05. Patients with markedly abnormal TUG results have an OR of 4.27 (95% CI 1.93 to 9.44), P<0.05. *Vesical residual volume (≥160 mL) is	⨁⨁◯◯ LOW	LIMITED IMPORTANCE

No. of studies	Certainty assessment						Effect			Certainty	Importance
	Study design	Risk of bias	Inconsistency	Indirectness	Imprecision	Other considerations	No. of events	No. of individuals	Rate (95% CI)		
									associated with an increased risk of mortality (OR: 4.50, 95% CI 1.5-13.5), P<0.05.		
Complication after abdominal surgery (hospitalized elderly) (assessed with: ECOG)											
1[33]	observational studies	not serious	not serious	Serious[aaf]	not serious	all possible residual confounding factors could reduce the observed effect	100	718	Multivariate regression analysis.<br><br>ECOG (2–4) OR: 3.23 (95% CI 1.75-1.95), P<0.001.	⨁⨁◯◯ LOW	IMPORTANT
In-hospital development of pneumonia after major digestive surgery (assessed with: ECOG-PS)											
1[34]	observational studies	not serious	not serious	Serious[aag]	not serious	all possible residual confounding factors could reduce the observed effect	67	1016	Multivariate regression analysis.<br><br>ECOG-PS OR: 2.64 (95% CI 1.21-5.72), P=0.014.	⨁⨁◯◯ LOW	IMPORTANT
Major postoperative complications at 30 days in cancer patients undergoing colon surgery (assessed with: activities of daily living)											
1[35,36,37]	observational studies	not serious	not serious	Serious[aah,aai]	not serious	all possible residual confounding factors could reduce the observed effect	99 (Lee); 83 (Kristjansson)	240 (Lee); 178 (Kristjansson)	Multivariate regression analysis.<br><br>(Xue) ADL >3 OR: 1.69 (95% CI 1.20-2.38) in the meta-analysis.<br><br>(Lee) ADL OR: 3.37 (95% CI 1.1 to 10.35), P<0.05.<br><br>(Kristjansson) Activities of daily living OR: 1.57 (95% CI 1.1-2.26), P<0.05.<br><br>ECOG-PS: OR: 2.64 (95% CI 1.21-5.72), P=0.014.	⨁⨁⨁◯ MODERATE	IMPORTANT

6MWT, 6-minute walk test; ASA, American Society of Anesthesiologists; ECOG, Eastern Cooperative Oncology Group; EORTC, European Organization for Research and Treatment of Cancer; KPS, Karnofsky Performance Scale; MNA-SF, mini nutritional assessment short form; SF-36, MELD, model for end-stage liver disease; MUST, malnutrition universal screening tool; PNI, Prognostic Nutritional Index; SGA, subjective global assessment; SPPB, short physical performance battery; TUG, timed up and go test.

Explanations

a. Observational study with a good sample size. The primary objective is the prognostic value of functional tests.

b. The population is limited to elderly and hospitalized patients. However, the malnutrition rate is 63.8%, which represents patients with a diagnosis of nutritional risk, supporting the generalizability of the results.

c. The functional test could not be per formed in 135 patients and a value of 0 m was assigned, which may condition the results. There are no analysis data excluding these patients.

d. Although the population is hospitalized elderly, 67% were malnourished.

e. Population had a mean age of 80.4 years and lived in a long-term care facility. The results cannot be extrapolated to the overall population. 90% had malnutrition.

f. The risk of dependence in activities of daily living is analyzed in the univariate regression model but is not included in the final multivariate regression model.

g. Multivariate Logistic Regression Models for Death: No significance.

h. The quality was downgraded due to imprecision in the results.

i. Elderly oncology patients with a mean age of 77.8 years. 41.9% of patients are malnourished, per MNA.

j. Applicable only to solid tumors.

k. Elderly oncology patients with a mean age of 80.2 years. 54.4% were malnourished per MNA.

l. Oncologic population with a mean age of 60.5 years. 60% of patients were malnourished per SGA (B+C).

m. Mortality rate is not described.

n. Population limited to the elderly (mean age: 83 years), a single center, and with limitations in the diagnosis of malnutrition (18.9% malnourished per Hospital Safety Management frailty scoring system).

o. Other factors influence the mortality event, such as age, sex, comorbidities (Charlson Comorbidity Index) and surgical risk (ASA) that affect functionality.

p. Very wide confidence intervals in multivariate analysis.

q. Statistical significance is lost in multivariate analysis.

r. No data on percentage of malnourished patients. Sarcopenia is analyzed and there is no direct analysis of functional tests. The population is very specific (patients on peritoneal dialysis), with malnutrition-inflammation-atherosclerosis syndrome.

s. Age is missing.

t. Very wide confidence interval, which limits the interpretation of the results.

u. Missing information on how it is measured.

v. Speed (m/s): 1.68 ± 0.5 in patients without sarcopenia vs 1.18 ± 0.64 in sarcopenic patients (P=0.007). Speed was 1.67 ± 0.51 in non-frail patients vs 0.79 ± 0.30 in frail patients (P=0.001).

w. Mean population age was 84 years.

x. Small sample size but considerable mortality and evidence limited to univariate analysis. Confidence interval at the limit of significance.

y. Mean population age was 48.7 years. Rate of malnutrition is not stated, but BMI (mean 23.1) and albumin levels (4.15 in survivors vs 3.98 in non-survivors) were measured.

z. Mean population age was 64 years. It does not indicate the rate of malnutrition although BMI and albumin levels were measured.

aa. Mean population age was 64 years. The rate of malnutrition is not stated, but albumin levels (mean 4.3 mg/dl) and BMI (mean 28.6) were measured, which indicate a low nutritional risk in the population.

ab. Malnutrition was 15.9% per SGA and 25.1% per MNA. Low prevalence of malnutrition in the sample.

ac. The confidence interval is very wide and the variable is presented transformed (Log[n]).

ad. Mean population age was 61.3 years. 54% of patients were malnourished per CONUT (analytical screening tool with no direct malnutrition data).

ae. Elderly population (mean age: 86 years) in the context of hospital admission for acute coronary syndrome, which limits the generalizability of the results. 44% of patients were at nutritional risk per MNA.

af. Data from 2 populations with ACS were used: Fraser Study (4 hospitals) in Italy and Longevo Study (44 hospitals) in Spain.

ag. Elderly population with a mean age of 80.3 years. The Geriatric Nutritional Risk Index was used. Nutritional assessment data are not available.

ah. Elderly population with a mean age of 82.4 years. Nutritional status was not assessed directly but patients had albumin <3 mg/dL (65%), lymphocytes <1,500 cells/µl (37%) and a mean BMI of 23.4. 52% of patients had cardiovascular disease and 18% have a neurodegenerative illness. Cancer patients were excluded.

ai. Mean population age was 55.9 years; patients underwent lung transplantation. Nutritional risk is not assessed.

aj. Mean population age was 60 years; patients with advanced liver disease. The proportion of patients with Child-Pugh Class A, B and C were 9%, 61% and 30%, respectively.

ak. Nutritional status is not assessed but a considerable weight loss is reported in 40–60% of patients, which suggests they were at nutritional risk.

al. Mean population age was 52 years; patients with advanced liver disease.

am. 59% of patients had inadequate nutritional status per SGA.

an. The small sample size and the wide confidence interval may limit the precision of the study.

ao. Mean population age was 32 years. No precise diagnostic criteria for malnutrition were used. Mean BMI is 18.9 with mid-upper arm circumference of 23 cm. 64% of patients had weight loss and 50% had appetite loss.

ap. Wide confidence intervals, which may limit the accuracy of results.

aq. Elderly population with a mean age of 83 years, with hospital admissions (infections and heart failure); not completely applicable to the general population. 57.1% of patients were diagnosed with malnutrition.

ar. Very wide confidence interval.

as. Large sample size; 52.6% of patients had sarcopenia, 15% were underweight (BMI <18.5), and 38% were over 85 years old.

at. Very elderly population with a mean age of 82.6 years and with 31.7% at risk of malnutrition, which limits the extrapolation of the results to the general population.

au. The study population consists of patients in the ICU over 65 years of age, which limits the extrapolation of the results to the general population. 56% of patients were at risk of malnutrition.

av. Heterogeneous population (multiple reasons for hospital admission), elderly (mean age 82 years), single center.

aw. Although functional impairment is important, in the case of hospitalized patients, the severity of the disease resulting in admission was a strong determinant of mortality.

ax. Significance limit P=0.0507.

ay. Elderly population; data on prevalence of malnutrition (SNAQ score) are not stated. Median is 0, as the IQR ranges from 0 to 1. Median age is missing.

az. Elderly cancer patients aged ≥70 years. Malnutrition is present in 59% of cases, assessed by MNA-SF.

aaa. The clinical population consist of palliative cancer patients with a mean age of 60 years. Assessment of nutritional status is not described, but 32% of patients had weight loss.

aab. Patient population with acute stroke in the hospital setting with a 42% malnutrition rate at admission.

aac. The population is elderly, with a mean age of 83.2 years, which limits the extrapolation of results to the overall population. The study concerns general admissions for different illnesses (mainly respiratory and gastrointestinal) with a considerable malnutrition rate (median MNA-SF of 9 and 77% of patients with sarcopenia).

aad. Very wide confidence interval, which may limit the quality of the evidence.

aae. The population consists of hospitalized women over 65 years of age. Increased bladder residual volume is an indirect parameter associated with increased risk of complications and mortality.

aaf. The population is elderly, with a mean age of 71 years. Nutritional risk is analyzed with CONUT. Clinical criteria for malnutrition are not evaluated. 36% of major abdominal surgery is conducted in the lower digestive tract and 64% in the upper digestive tract. 50% of surgeries are laparoscopic.

aag. The population is elderly with a mean of 70 years. Malnutrition was assessed using CONUT and PNI. Malnutrition rates are not reported, but nutritional risk was high, given the population had undergone major abdominal surgery.

aah. The clinical population consists of elderly cancer patients with a mean age of 79.6 years (Kristjansson) and 76 (Lee) who underwent colon surgery. Surgery per site was: 71% of patients in colon and 29% in rectum (Kristjansson); 62.5% (colon) and 37.5% (rectum) (Lee).

aai. Nutritional status was not assessed.

References

1. Martín-Ponce E, Hernández-Betancor I, González-Reimers E, Hernández-Luis R, Martínez-Riera A, Santolaria F. Prognostic value of physical function tests: hand grip strength and six-minute walking test in elderly hospitalized patients. Sci Rep. 2014;4:7530.

2. Hernández-Luis R, Martín-Ponce E, Monereo-Muñoz M, Quintero-Platt G, Odeh-Santana S, González-Reimers E, et al. Prognostic value of physical function tests and muscle mass in elderly hospitalized patients. A prospective observational study. GeriatrGerontol Int. enero de 2018;18(1):57-64.

3. Moon S, Hong G-RS. Predictive Factors of Mortality in Older Adult Residents of Long-Term Care Facilities. J Nurs Res JNR. abril de 2020;28(2):82.

4. Edwards BJ, Zhang X, Sun M, Song H, Khalil P, Sri Laruturi M et al. Overall survival in older patients with cancer. BMJ Supportive & Palliative Care. 2018; 10(1):25-35.

5. Ferrat E, Paillaud E, Laurent M, et al. Predictors of 1-Year Mortality in a Prospective Cohort of Elderly Patients With Cancer. J Gerontol A BiolSciMedSci. 2015;70(9):1148-1155.

6. Sánchez-Lara K, Turcott JG, Juárez E, et al. Association of nutrition parameters including bioelectrical impedance and systemic inflammatory response with quality of life and prognosis in patients with advanced non-small-cell lung cancer: a prospective study. NutrCancer. 2012;64(4):526-534.

7. Folbert EC, Hegeman JH, Vermeer M, Regtuijt EM, van der Velde D, Ten Duis HJ, et al. Improved 1-year mortality in elderly patients with a hip fracture following integrated orthogeriatric treatment. Osteoporos Int J Establ Result Coop Eur Found Osteoporos Natl Osteoporos Found USA. 2017;28(1):269-77.

8. Kamijo Y, Kanda E, Ishibashi Y, Yoshida M. Sarcopenia and Frailty in PD: Impact on Mortality, Malnutrition, and Inflammation. Perit Dial Int J Int Soc Perit Dial. diciembre de 2018;38(6):447-54.

9. Rubio Rubio MV, Lou Arnal LM, Gimeno Orna JA, et al. Survival and quality of life in elderly patients in conservative management. Supervivencia y calidad de vida en pacientes ancianos en tratamiento renal conservador. Nefrologia. 2019;39(2):141-150.

10. Guney I, Atalay H, Solak Y, Altintepe L, Tonbul HZ, Turk S. Poor quality of life is associated with increased mortality in maintenance hemodialysis patients: a prospective cohort study. Saudi J KidneyDisTranspl. 2012;23(3):493-499.

11. Van Loon IN, Bots ML, Boereboom FTJ, et al. Quality of life as indicator of poor outcome in hemodialysis: relation with mortality in different age groups. BMC Nephrol. 2017;18(1):217.

12. Jesky MD, Dutton M, Dasgupta I, et al. Health-Related Quality of Life Impacts Mortality but Not Progression to End-Stage Renal Disease in Pre-Dialysis Chronic Kidney Disease: A Prospective Observational Study. PLoS One. 2016;11(11):e0165675.

13. Joaquín C, Puig R, Gastelurrutia P, et al. Mini nutritional assessment is a better predictor of mortality than subjective global assessment in heart failure out-patients. Clin Nutr. 2019;38(6):2740-2746.

14. La Rovere MT, Maestri R, Olmetti F, et al. Additional predictive value of nutritional status in the prognostic assessment of heart failure patients. Nutr Metab Cardiovasc Dis. 2017;27(3):274-280.

15. Tonet E, Campo G, Maietti E, et al. Nutritional status and all-cause mortality in older adults with acute coronary syndrome. ClinNutr. 2020;39(5):1572-1579.

16. Matsuzawa R, Kamiya K, Hamazaki N, et al. Office-Based Physical Assessment in Patients Aged 75 Years and Older with Cardiovascular Disease. Gerontology. 2019;65(2):128-135.

17. Cortés-Flores AO, Álvarez-Villaseñor A del S, Fuentes-Orozco C, et al. Long-term outcome after percutaneous endoscopic gastrostomy in geriatric Mexican patients. Geriatr Gerontol Int. 2015;15(1):19-26.

18. Venado A, Kolaitis NA, Huang CY, et al. Frailty after lung transplantation is associated with impaired health-related quality of life and mortality. Thorax. 2020;75(8):669-678.

19. Lai JC, Feng S, Terrault NA, Lizaola B, Hayssen H, Covinsky K. Frailty predicts waitlist mortality in liver transplant candidates. Am J Transplant. 2014;14(8):1870-1879.

20. Ribeiro HS, Maurício SF, Antônio da Silva T, de Vasconcelos Generoso S, Lima AS, Toulson Davisson Correia MI. Combined nutritional assessment methods to predict clinical outcomes in patients on the waiting list for liver transplantation. Nutrition. 2018;47:21-26.

21. Reepalu A, Balcha TT, Skogmar S, Güner N, Sturegård E, Björkman P. Factors Associated with Early Mortality in HIV-Positive Men and Women Investigated for Tuberculosis at Ethiopian Health Centers. PLoSOne. 2016;11(6):e0156602. Published 2016 Jun 7.

22. Asensio A, Ramos A, Núñez S. Factores pronósticos de mortalidad relacionados con el estado nutricional en ancianos hospitalizados [Prognostic factors for mortality related to nutritional status in the hospitalized elderly]. Med Clin (Barc). 2004;123(10):370-373.

23. Wakabayashi H, Maeda K, Nishioka S, Shamoto H, Momosaki R. Impact of Body Mass Index on Activities of Daily Living in Inpatients with Acute Heart Failure. J Nutr Health Aging. 2019;23(2):151-6.

24. Calvo E, Teruel L, Rosenfeld L, et al. Frailty in elderly patients undergoing primary percutaneous coronary intervention. Eur J Cardiovasc Nurs. 2019;18(2):132-139.

25. Giannasi SE, Venuti MS, Midley AD, Roux N, Kecskes C, San Román E. Mortality risk factors in elderly patients in intensive care without limitation of therapeutic effort. Factores de riesgo de mortalidad de los pacientes ancianos en cuidados intensivos sin limitación del esfuerzo de tratamiento. Med Intensiva. 2018;42(8):482-489.

26. Comba M, Fonte G, Isaia G, Pricop L, Sciarrillo I, Michelis G, et al. Cardiac and inflammatory biomarkers and in-hospital mortality in older medical patients. J Am Med Dir Assoc.2014;15(1):68-72.

27. Reijnierse EM, Verlaan S, Pham VK, Lim WK, Meskers CGM, Maier AB. Lower Skeletal Muscle Mass at Admission Independently Predicts Falls and Mortality 3 Months Post-discharge in Hospitalized Older Patients. J Gerontol A Biol Sci Med Sci. 2019;74(10):1650-6.

28. Quinten C, Kenis C, Decoster L, et al. The prognostic value of patient-reported Health-Related Quality of Life and Geriatric Assessment in predicting early death in 6769 older (≥70 years) patients with different cancer tumors. J Geriatr Oncol. 2020;11(6):926-936.

29. Miner TJ, Brennan MF, Jaques DP. A prospective, symptom related, outcomes analysis of 1022 palliative procedures for advanced cancer. Ann Surg. 2004;240(4):719-727.

30. Sato M, Ido Y, Yoshimura Y, Mutai H. Relationship of Malnutrition During Hospitalization With Functional Recovery and Postdischarge Destination in Elderly Stroke Patients. J Stroke Cerebrovasc Dis Off J Natl Stroke Assoc.2019;28(7):1866-72.

31. Maeda K, Takaki M, Akagi J. Decreased Skeletal Muscle Mass and Risk Factors of Sarcopenic Dysphagia: A Prospective Observational Cohort Study. J Gerontol A Biol Sci Med Sci. 2017;72(9):1290-1294.

32. Nuotio MS, Luukkaala T, Tammela T. Elevated post-void residual volume in a geriatric post-hip fracture assessment in women-associated factors and risk of mortality. Aging Clin Exp Res. 2019;31(1):75-83.

33. Nishijima M, Baba H, Murotani K, et al. Early ambulation after general and digestive surgery: a retrospective single-center study. Langenbecks Arch Surg. 2020;405(5):613-622.

34. Baba H, Tokai R, Hirano K, et al. Risk factors for postoperative pneumonia after general and digestive surgery: a retrospective single-center study. Surg Today. 2020;50(5):460-468.

35. Xue DD, Cheng Y, Wu M, Zhang Y. Comprehensive geriatric assessment prediction of postoperative complications in gastrointestinal cancer patients: a meta-analysis. Clin Interv Aging. 2018;13:723-736.

36. Lee YH, Oh HK, Kim DW, et al. Use of a comprehensive geriatric assessment to predict short-term postoperative outcome in elderly patients with colorectal cancer. Ann Coloproctol. 2016;32(5):161–169.

37. Kristjansson SR, Jordhøy MS, Nesbakken A, et al. Which elements of a comprehensive geriatric assessment (CGA) predict post-operative complications and early mortality after colorectal cancer surgery? J Geriatr Oncol. 2010;1(2):57–65.
