# Supplementary material for: Expert Consensus on Morphofunctional Assessment in Disease-Related Malnutrition. Grade Review and Delphi Study
Source: Nutrients. 2023 Jan 25;15(3):612. doi: 10.3390/nu15030612 (PMC9920608; doi:10.3390/nu15030612)
Supplement: Supplementary file 1 [file nutrients-15-00612-s001.zip › SUPPLEMENTARY INFORMATION S3.pdf]

SUPPLEMENTARY INFORMATION S3

List of Delphi participants

Ángel Luis Abad González, Mirian Alejo Ramos, José Joaquín Alfaro Martínez, Jara Altemir Trallero, Julia Álvarez Hernández, María Álvarez Marín, Meneses Amparo Lomas, Ortega Antonio Martínez, Carmen Aragón Valera, María Argente Pla, Rosa Argueso Armesto, Elena Arjonilla Sampedro, Claudia Arnás León, Marta Arráez Monllor, Carmen Arraiza Irigoyen, Ana Artero Fullana, Raúl Ballester Sajardo, M. Dolores Ballesteros Pomar, David Barajas Global, Ricardo Batanero Maguregui, Virginia Bellido Castañeda, Diego Bellido Guerrero, María Berrío Miranda, Diana Boj Carceller, José Ignacio Botella Carretero, Francisco Botella Romero, Irene Bretón Lesmes, Alfonso Calañas Continente, Alicia Calleja Fernández, Laura Araceli Calles Romero, Miguel Camblor Álvarez, Cristina Campos Martín, Javier Campuzano Jara, Emilia Cancer Minchot, Bárbara Cánovas Gaillemin, Soralla Civantos Modino, Miguel Civera Andrés, Natalia Colomo, Victoria Contreras Bolivar, Ana Alejandra Cordero Vaquero, M. Isabel Cornejo Pareja, Laura Cotovad Bellas, Cristina De La Cuerda Compés, Antonio Daniel De Luis Román, Esther Delgado García, Patricia Díaz Guardiola, Margarita Díez Muñiz, Martín Eider Etxeberria, Ana Elbusto Cabello, Navarro Emilio Sánchez, Isabel Ester Escuer Núñez, Beatriz Fernández Medina, Natalia Fernández Romero, María Luisa Fernández Soto, Gracia Fernando Calvo, Pablo Ferreira Ocampo, Mercedes Ferrer Gómez, Luna Florencio Ojeda, Ángel Luis Fraile Sáez, José Manuel García Almeida, Lourdes García Blasco, Yaiza García Delgado, María García Domínguez, Pedro Pablo García Luna, Katerine García Malpartida, Ignacio García Puente, Francisca García Torres, M. Victoria García Zafra, Álvaro García-Manzanares Vázquez, Francisco Javier Gómez Alonso, Carmen Gómez Candela, Emilia Gómez Hoyos, Elena González Arnáiz, Margarita

González Boillos, Paloma González Lázaro, Inmaculada González Molero, Irene González Navarro, Beatriz González Rosa, Víctor Luis González Sánchez, Irene Gonzalo Montesinos, Juan Manuel Guardia Baena, Ana Hernández Moreno, María Elena Hernández Nieto, Irene Hoyas Rodriguez, Natacia Covadonga Iglesias Hernández, Rosa María Iglesias López, María José Jiménez Jiménez, Ana Jiménez Portilla, Laura Kanaan, María Lainez López, Beatríz Lardies Sánchez, Miguel León Sanz, Arturo Lisbona Catalán, Juan José López Gómez, Antonio López Guzmán, José Antonio López Medina, Mario López Merseguer, Ana López Navia, Luis López Penabad, Rafael López Urdiales, Francisco Losfablos Callau, Luis Miguel Luengo Pérez, María Maíz Jiménez, Cristina Maldonado Araque, Gonzalo Maldonado Castro, Cristian Marco Alacid, Amelia Marí Sanchis, Gonzalo Marín Montserrat, M. Ángeles Martín Almendra, Tomás Martín Folgueras, M. Ángeles Martín Palmero, Ceferino Martínez Faedo, Miguel Ángel Martínez Olmos, M. José Martínez Ramírez, Pilar Matía Martín, María Merino Viveros, Laura Mola Reyes, Begoña Molina Baena, M. José Molina Puerta, Jorge Monroy Sánchez, Inmaculada Moraga Guerrero, Francisco Moreno Baró, María Teresa Mories Álvarez, Marta Motilla de la Cámara, Concepción Muñóz Jiménez, Luisa Muñoz Salvador, M. Julia Ocón Bretón, Juana Olivar Roldán, Josefina Olivares Alcolea, Gabriel Olveira Fuster, Carlos Higinio Ortega Sánchez, Jorge Ortez Toro, José María Palacio Mures, Samara Palma Milla, Mercedes Palma Moya, Clara Palomares Avilés, Juan Parra Barona, Elena Parreño Caparrós, Inmaculada Peiró Martínez, José Luis Pereira Cunill, Eva Perelló Camacho, José Pérez Rodríguez, Carlos Miguel Peteiro Miranda, Estrella Petrina Jáuregui, María Picallo Pérez, Francisco Pita Gutiérrez, Joaquín Puerma Ruiz, Pedro Pujante Alarcón, Antelm Pujol Calafat, Roser Querol Ripoll, Rosa Quílez Taboso, Araceli Ramos Carrasco, Henry Rendon Barragán, María Riestra Fernández, Juan Manuel Romerosa Vico, Ignacio Ruiz

García, Lourdes Salinero González, Carlos Sánchez Juan, Ana Isabel Sánchez Marcos, Myriam Sánchez Pacheco, Olga Sánchez-Vilar Burdiel, Alejandro Sanz París, Clara Serrano Moreno, Esther Solano Fraile, Ana Gloria Soler Beunza, Lorena Suárez Gutiérrez, José Pablo Suárez Llanos, María Soledad Talavera Marín, M. José Tapia Guerrero, Cristina Tejera Pérez, Mónica Tomé García, Sara Torrejón Jaramillo, Nuria Valdés Gallego, M. Rosario Vallejo Mora, Luis Alberto Vázquez Salvi, Belén Vega Piñero, Alfonso Vidal Casariego, Concepción Vidal Peracho, Juan Francisco Vigueras Pérez, Francisco Villazón González, Viyey Doulatram, Beatriz Voltas Arribas, Ana Zugasti Murillo.
