# Supplementary material for: Expert Consensus on Morphofunctional Assessment in Disease-Related Malnutrition. Grade Review and Delphi Study
Source: Nutrients. 2023 Jan 25;15(3):612. doi: 10.3390/nu15030612 (PMC9920608; doi:10.3390/nu15030612)
Supplement: Supplementary file 1 [file nutrients-15-00612-s001.zip › SUPPLEMENTARY TABLE S1.pdf]

SUPPLEMENTARY TABLE S1. Analysis of quantitative and qualitative agreement between surveys by topic.

	Spearman correlation	p	Kappa index	p
TOTAL (37 statements)	0.846	<0.001	0.580	<0.001
Food intake and nutrient assimilation (5 statements)	0.545	<0.001	0.260	<0.001
Anthropometry (6 statements)	0.788	<0.001	0.597	<0.001
Biochemistry (9 statements)	0.922	<0.001	0.660	<0.001
Dynamometry (2 statements)	0.737	<0.001	0.462	<0.001
Body composition (2 statements)	0.787	<0.001	0.540	<0.001
Imaging (6 statements)	0.729	<0.001	0.517	<0.001
Functional status and quality of life (7 statements)	0.763	<0.001	0.447	<0.001
